# Electrochemical Nickel-Catalyzed C(sp^3^)–C(sp^3^) Cross-Coupling of Alkyl Halides with Alkyl Tosylates

**DOI:** 10.1021/jacs.3c07313

**Published:** 2023-07-26

**Authors:** Malek Y. S. Ibrahim, Graham R. Cumming, Raquel Gonzalez de Vega, Pablo Garcia-Losada, Oscar de Frutos, C. Oliver Kappe, David Cantillo

**Affiliations:** † Institute of Chemistry, University of Graz, NAWI Graz, Graz 8010, Austria; ‡ Center for Continuous Flow Synthesis and Processing (CCFLOW), Research Center Pharmaceutical Engineering GmbH (RCPE), Graz 8010, Austria; § Centro de Investigación Lilly S.A., Avda. de la Industria 30, 28108 Alcobendas-Madrid, Spain; ∥ TESLA-Analytical Chemistry, University of Graz, NAWI Graz, Graz 8010, Austria

## Abstract

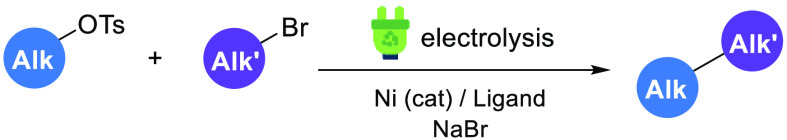

Formation of new C(sp^3^)–C(sp^3^) bonds is a powerful synthetic tool to increase molecular diversity, which is highly sought after in medicinal chemistry. Traditional generation of carbon nucleophiles and more modern cross-electrophile-coupling methods typically lack sufficient selectivity when cross-coupling of analogous C(sp^3^)-containing reactants is attempted. Herein, we present a nickel-catalyzed, electrochemically driven method for the coupling of alkyl bromides with alkyl tosylates. Selective cross-coupling transformations were achieved even between C(sp^3^)-secondary bromides and tosylates. Key to achieve high selectivity was the combination of the tosylates with sodium bromide as the supporting electrolyte, gradually generating small amounts of the more reactive bromide by substitution and ensuring that one of the reaction partners in the nickel-catalyzed electroreductive process is maintained in excess during a large part of the process. The method has been demonstrated for a wide range of substrates (>30 compounds) in moderate to good yields. Further expanding the scope of electroorganic synthesis to C(sp^3^)–C(sp^3^) cross-coupling reactions is anticipated to facilitate the switch to green organic synthesis and encourage future innovative electrochemical transformations.

Over the past years, the development of novel methodologies for the selective formation of alkyl–alkyl bonds has become an important area of research.^1^ Cross-coupling of sp^3^-hybridized carbons enables the rapid diversification of molecular structures, which is very relevant in the discovery and preparation of drug candidates. Traditional nickel- and palladium-catalyzed couplings using Grignard reagents (Scheme 1a) typically suffer from poor functional group tolerance due to the high reactivity of organometallic compounds.^2^ Important efforts to circumvent the use of organometallic reagents have been made in the past years. Gong and co-workers coupled bromides using an excess amount of Zn or bis(pinacolato)diboron as reductant.^3^ Successful cross-electrophile coupling between alkyl halides and tosylates was reported by Komeyama et al.,^4^ although the method required excess amounts of a metal reductant and cobalamin as cocatalyst (Scheme 1b). A similar strategy was described by Fu and Liu using a copper catalyst and stoichiometric amounts of magnesium and lithium methoxide.^5^ An iridium/nickel dual photocatalyst system has been recently shown by MacMillan and co-workers to enable alcohol–bromide couplings.^6^ Despite the recent advances, while metal-catalyzed cross-electrophile coupling reactions involving sp^2^ carbons are well established, formation of alkyl–alkyl bonds has remained a challenge.^7^ Nickel-catalyzed methods are usually preferred over other metals due to their lower cost and ability to undergo oxidative addition with alkyl halides under mild conditions. Moreover, nickel species in solution can be reduced with ease using metal reductants such as Zn^8^ or Mn.^9^ Although this strategy is successful in driving the catalytic cycle, it often suffers from low selectivity due to the formation of homocoupling products. Additionally, the effective activation of the metal reductant might become complex in large-scale preparations.

**Scheme 1:**
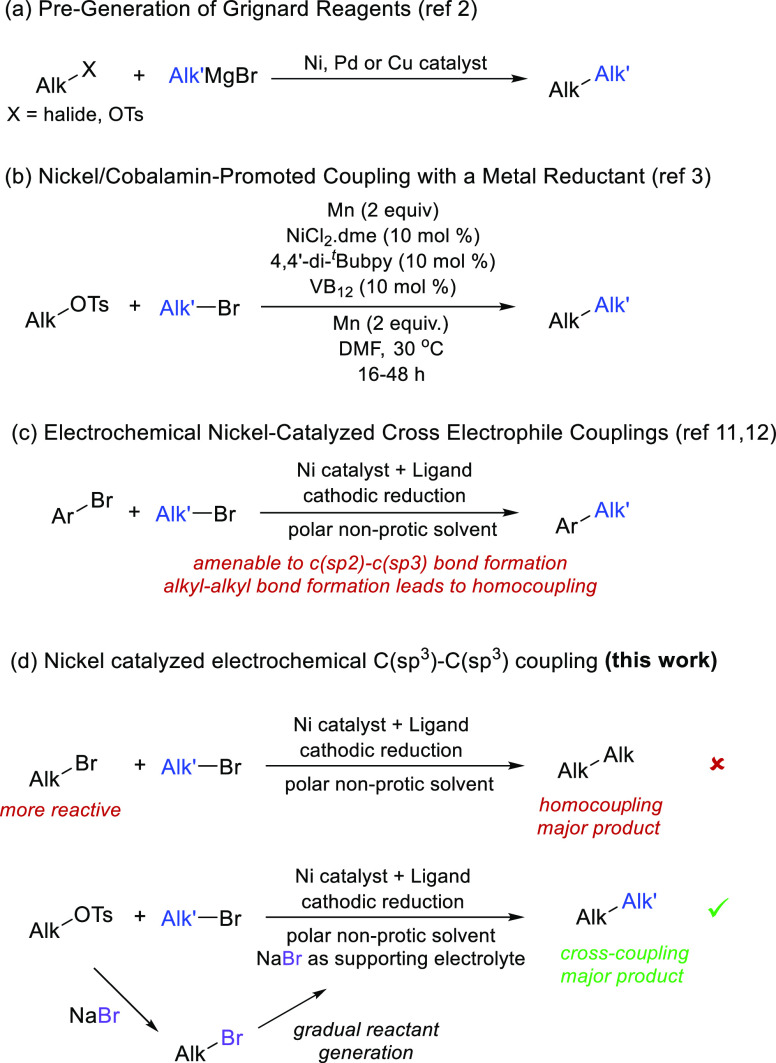
Challenges in C(sp^3^)–C(sp^3^) Cross-Electrophile Coupling

Electroorganic synthesis is rapidly growing as a safe and sustainable synthetic technology for the preparation of organic compounds,^10^ including pharmaceutical ingredients.^11^ Among the many redox transformations achieved electrochemically over the past years,^12^ metal-catalyzed strategies for the formation of carbon–carbon bonds have been studied extensively.^13^ Electrochemical cross-electrophile coupling of organic halides has been reported by using nickel catalysts. Yet, reports have mainly focused on C(sp^2^)–C(sp^3^) couplings,^14,15^ as alkyl–alkyl bond-forming reactions lack selectivity versus undesired homocoupling (Scheme 1c). Lin and co-workers have recently achieved electroreductive C(sp^3^)–C(sp^3^) cross-coupling of alkyl halides by taking advantage of the fact that more substituted alkyl halides are more easily reduced to the corresponding carbanions.^16^

We envisaged that instead of selecting the thermodynamic properties of the reactants involved in the cross-coupling, altering the reaction kinetics by gradually generating one of the reactants within the reaction mixture during electrolysis could also be used to achieve high selectivity. In particular, cross-couplings between alkyl bromides that are not selective could be turned selective by generating the more reactive bromide from the corresponding tosylate by tosylate/bromide exchange with an inexpensive bromide source that also serves as supporting electrolyte (Scheme 1d).

To test our hypothesis, we initiated the investigation by studying the coupling of alkyl tosylate **1a** with cyclohexyl bromide (**1b**) as a model reaction (Table 1). In a typical experiment, the reaction mixture was electrolyzed under an argon atmosphere under a constant current of 4 mA (ca. 2.7 mA cm^–2^), until 3.0 F mol^–1^ of charge had been passed, using a glassy-carbon cathode and an aluminum anode. Gratifyingly, the electrochemical reaction resulted in the formation of target cross-coupling product **1c** with 79% yield (calibrated GC-FID) (Table 1, entry 1). Analysis of the reaction mixture revealed the presence of styrene and 1,4-diphenylbutane as the main side products formed from **1a** by elimination and homocoupling, respectively. These results are in contrast with those obtained using (2-bromoethyl)benzene directly as substrate, which resulted in homocoupling as the major product (vide infra).

**Table 1:**
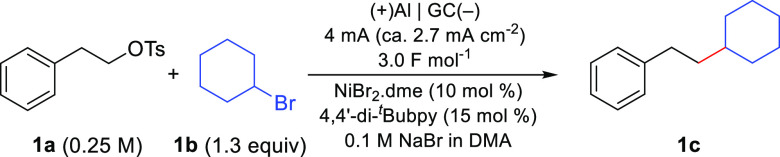
Optimization of Cross-Coupling Conditions

entry	deviation from the above	**1c** (%)^b^
1	none	79
2	10 mA	53
**3**	2 mA	8
4	RVC cathode	66
5	graphite cathode	74
6	Zn anode	24
7	Mg anode	46
8	30 mol % ligand	45
9	nBu_4_NPF_6_ as supporting electrolyte	2
10	nBu_4_NCl as supporting electrolyte	42
11	nBu_4_NI as supporting electrolyte	66
12	Et_4_NOTs as supporting electrolyte	39
13	L1	65
14	L2	61
15	L3	45
16	L4	69
17	L5	37
18	complex **1**	6

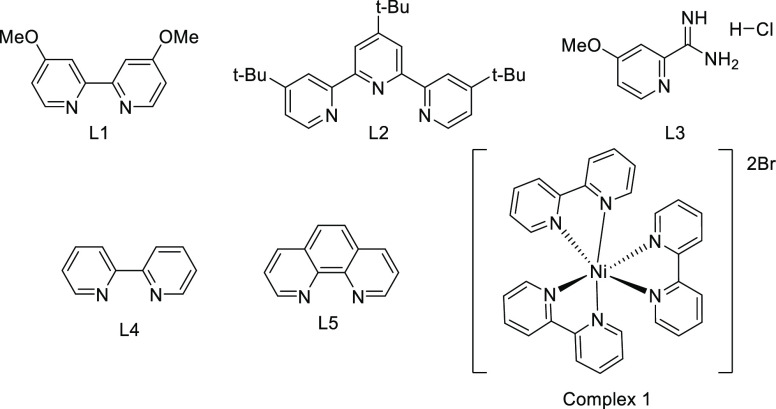

a Conditions: 3 mL volume, 600 rpm, rt, under Ar.

b Determined by calibrated GC-FID.

Notably, an increase or decrease of the current density resulted in a decrease in yield (Table 1, entries 2 and 3). Glassy carbon was the optimum cathode material, but satisfactory results were also achieved with RVC or graphite (entries 4 and 5). The choice of a sacrificial anode material proved to be very important. When zinc or magnesium (entries 6 and 7) were used, the formation of **1c** dropped below 50%. In principle, the role of the sacrificial anode is to undergo oxidation as a counter electrode, and the reason for the improved performance of aluminum is not clear. No evidence was found for the direct participation of aluminum salts in the reaction mechanism. Solvents other than DMA did not perform well in the reaction (Table S2).

Increasing the ligand/nickel ratio to 3/1 had a negative effect (Table 1, entry 8), probably due to catalyst deactivation. As expected, when a nonhalide salt (nBu_4_NPF_6_) was used as supporting electrolyte (entry 9), the yield dropped to 2%. This observation is in line with the initial hypothesis that a tosylate–halide exchange initiates the reaction. The use of chloride- or iodide-containing supporting electrolytes also negatively impacted the reaction (entries 10 and 11). Iodide salts resulted in higher amounts of styrene, likely due to easier elimination. The use of chloride augmented the proportion of homocoupling (Table S3). A 0.1 M concentration of NaBr was found to be optimal (Table S4). Replacing NiBr_2_·dme with other nickel halide salts decreased the amount of **1c** (Table S5). This is likely due to the fact that the halide anions from the catalyst can also participate in the exchange with tosylate. The ligand employed in the reaction had a lower impact on the reaction outcome than expected (entries 13–17). Although 4-methoxypicolinimidamide hydrochloride (L3) has shown good reactivity in other electrochemical Ni-catalyzed cross-coupling reactions,^11^ only 45% of **1c** was formed in this case (entry 15). 2,2′-Bipyridine (L4) gave analogous results, while 1,10-phenanthroline decreased the yield to 37% (entries 16 and 17). Surprisingly, complex **1** provided much poorer results than did L4 (entry 18).

Interestingly, direct reuse of the glassy-carbon electrodes (after rinsing with solvent) resulted in a decrease in the reaction yield by ca. 10%. The yield did not decline any further upon reuse of the electrode for a third and fourth time. This issue was initially ascribed to grafting of the carbon surface to alkyl radicals. However, analysis of the electrode surface by SEM-EDX and LA-ICP-MS revealed that the carbon surface of the electrode had most likely been brominated (Figure S2). This layer could not be eliminated by polishing without damaging the electrode surface.

With the optimized conditions in hand, the scope of the cross-coupling reaction was investigated (Scheme 2). Alkylation of **1a** with cyclopentyl, hexyl, and neopentyl bromide furnished the desired products **2c**–**4c** in good yields. However, alkylation with bromocyclopropane or 2-bromoethyl methyl ether performed poorly (**5c**, **6c**) due to rapid debromination of these reactants under the reaction conditions. The same issue was observed with 2-bromoethyl acetate (**8c**). Nitrogen-containing bromides could be used as coupling partners, giving moderate yields of **9c** and **10c**.

**Scheme 2:**
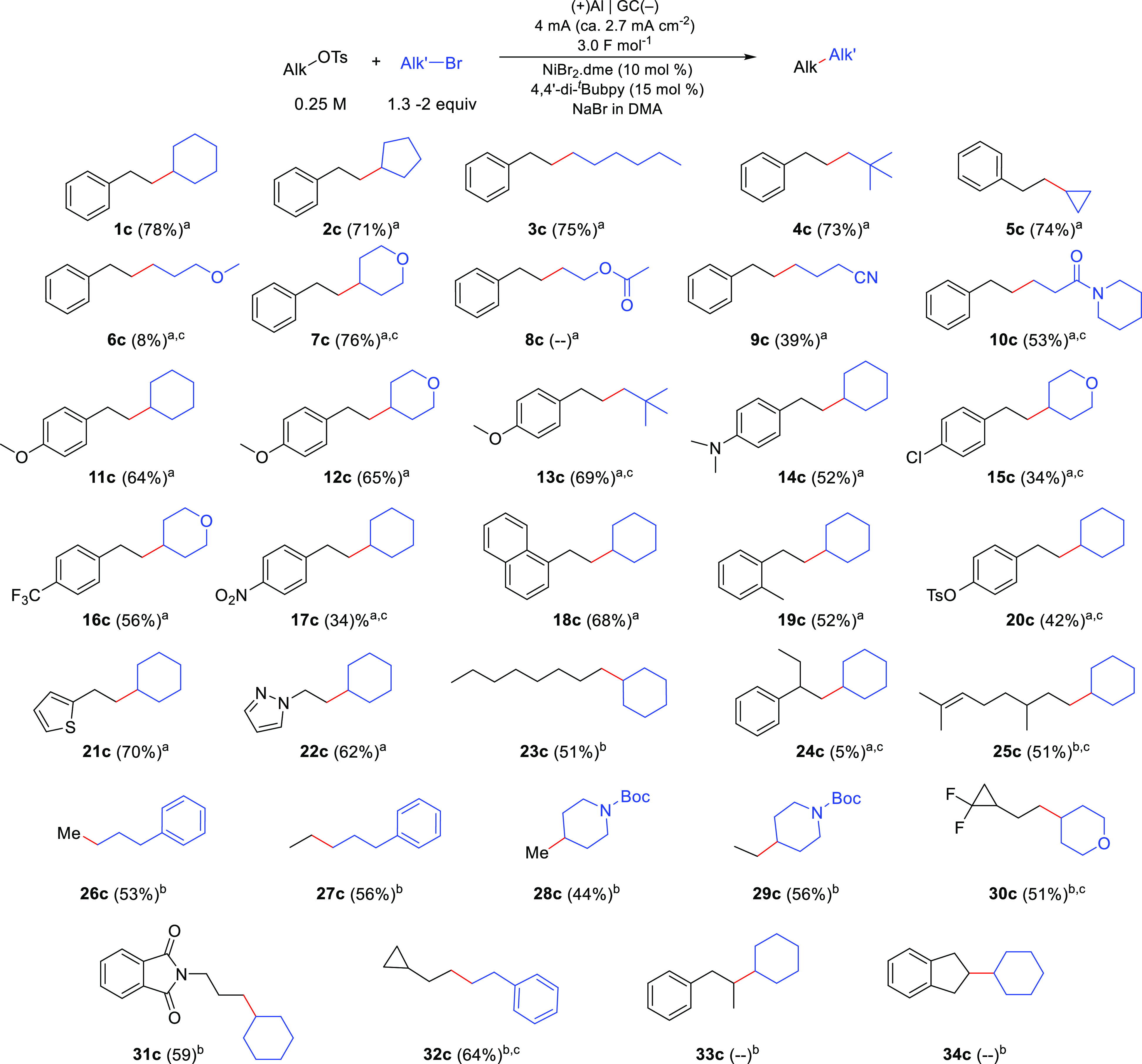
Scope of the Electrochemical Cross-Coupling of Alkyl Tosylates with Alkyl Bromides Conditions A: 3 mL volume, 0.1 M NaBr, 1.3 equiv alkyl bromide, 600 rpm, under Ar. Conditions B: same as conditions A with 0.075 M NaBr and 2 equiv alkyl bromide. GC-FID yield using biphenyl as internal standard. Isolated yields are shown.

A variety of functionalized 2-ethylarene-tosylates were also tested. The method proved to be compatible with electron-rich aromatics (**11c**–**14c**). The presence of halides or nitro groups did not inhibit the reaction (**15c**–**17c**). However, partial nitro reduction was observed during the formation of **17c**. Compounds containing naphthyl (**18c**) or methylaryl (**19c**) groups could also be prepared in good yields. As anticipated, when a reactant containing both an aryl- and alkyl-tosyl group was tested, the reaction proceeded selectively at the alkyl tosylate as only that position can undergo tosylate-bromide exchange (**20c**). Sulfur- and nitrogen-containing heterocycles could also be successfully incorporated into the coupling products (**21c**, **22c**).

Surprisingly, a secondary tosylate resulted in a very low yield under the standard reaction conditions (**24c**). A relatively large amount of tosylate was still detected by GC analysis after the reaction. In contrast, other primary tosylates such as that resulting from citronellol (**25c**) gave large amounts of tosylate homocoupling. This variability was ascribed to different rates of tosylate–bromide exchange depending on the substrate. To shed light onto this issue, the reactivity of several tosylates toward NaBr was studied in the absence of current (Figure S3). It was found that substrates with a fast OTs–Br exchange rate perform better with a lower loading of NaBr (0.075 M instead of 0.1 M) and a larger excess of the alkyl bromide reaction partner (Conditions B in Scheme 2) (Tables S6 and S7).

Methylation is a very important structural modification often used in medicinal chemistry.^17^ To our delight, our methodology could be used to realize methylation and ethylation of alkyl bromides using commercially available methyl and ethyl tosylates as alkylating agents (**26c**–**29c**). Additionally, the method was amenable to the preparation of compounds decorated with 2,2-difluorocyclopropane (**30c**) and phthalimide (**31c**). It was further confirmed that the method does not perform well with secondary tosylates (**33c**, **34c**).

To confirm our hypothesis that gradual tosylate–bromide exchange is responsible for the cross-coupling selectivity obtained, the concentration of all components was monitored for the model reaction (Figure 1a). As expected, the formation of bromide **1f** and its subsequent disappearance was observed. Furthermore, when **1f** was used directly as substrate (Figure 1b) side product **1e**, resulting from homocoupling of **1f**, was observed as the main product, supporting the key role of the gradual tosylate–bromide exchange in the reaction selectivity. The more reactive cross-coupling alkyl bromide pair corresponds to the reactant that is added as tosylate (**1a**). In this manner, as the bromide is generated in small amounts, it encounters an excess of the other reactant (**1b**). The alkyl bromide that is less reactive (**1b**) can also undergo oxidative addition, although more slowly, and it is indeed consumed by the end of the reaction, partially generating bicyclohexyl as a side product. Tosylate/iodide exchange has been previously suggested as a step in reductive aryl–alkyl couplings.^18^

**Figure 1:**
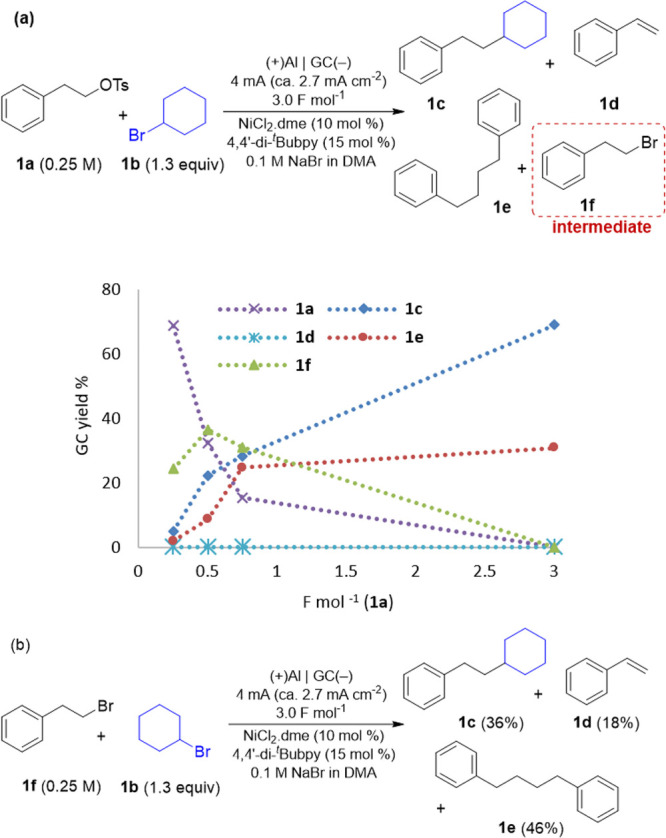
(a) Monitoring of the reaction mixture during the cross-coupling of **1a** with **1b** (Table 1, entry 1). (b) Control experiment using bromide **1f** instead of tosylate **1a**.

Based on the experiments above and literature data on nickel-catalyzed reductive processes,^14,19,20^ the reaction mechanism depicted in Figure 2 was proposed. The process is initiated by the cathodic reduction of **C1**, giving nickel(0) species **C2**. Tosylate–bromide exchange forms the more reactive alkyl bromide, which undergoes oxidative addition to **C2**, resulting in **C3**,^21^ which is further oxidized to nickel(III) (**C4**) upon addition of the alkyl radical produced by reduction of the coupling partner. The cross-coupling product is released by the reductive elimination from **C4**. The catalytic cycle closes by the redox reaction between **C5** and the alkyl halide coupling partner. Notably, no reaction occurred when the substrates were added after prereduction of the nickel, suggesting a short lifetime of the reduced Ni species in the absence of oxidants.^22^

**Figure 2:**
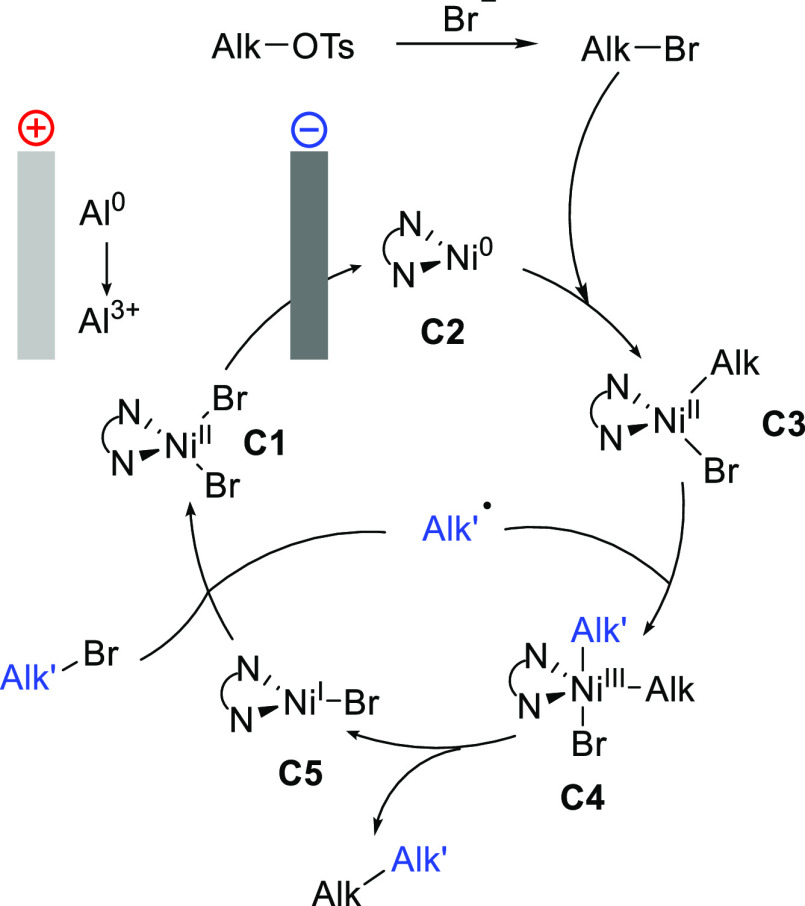
Proposed mechanism for electrochemical cross-coupling.

To summarize, we have demonstrated that selective electrochemical nickel-catalyzed C(sp^3^)–C(sp^3^) cross-coupling reactions are enabled when alkyl halides and tosylates are combined with a bromide supporting electrolyte. Key to achieving good selectivity is the progressive tosylate–bromide exchange taking place during the electrolysis, with a lower concentration of the reactive intermediate being sufficient for oxidative addition toward nickel(0), while disfavoring the homocoupling pathway. Utilization of Al as a sacrificial electrode instead of metal reductants such as Zn or Mg avoids the need for activation of the metal surface and enables the use of a less reactive and more cost-effective material. Given the growing importance of alkyl–alkyl bond forming reactions in medicinal chemistry, we anticipate that this contribution will further encourage the adoption of electroorganic synthesis in organic chemistry laboratories.

## Supporting Information Available

Materials and methods, additional optimization data, characterization data for the cathode material, experimental procedures, characterization data, and NMR spectra (PDF)
