# Supplementary material for: Electrochemical Nickel-Catalyzed C(sp3)–C(sp3) Cross-Coupling of Alkyl Halides with Alkyl Tosylates
Source: J Am Chem Soc. 2023 Jul 26;145(31):17023–8. doi: 10.1021/jacs.3c07313 (PMC10416217; doi:10.1021/jacs.3c07313)
Supplement: Supplementary file 1 — ja3c07313_si_001.pdf [file ja3c07313_si_001.pdf]

Supporting Information

Electrochemical Nickel-Catalyzed C(sp^3)-C(sp^3) Cross-Coupling of Alkyl Halides with Alkyl Tosylates

Malek Y. S. Ibrahim,[a,b] Graham R. Cumming,[c] Raquel Gonzalez de Vega,[d] Pablo Garcia-Losada,[c] Oscar de Frutos,[c] C. Oliver Kappe, [a,b] David Cantillo [a,b]*

[a] Institute of Chemistry, University of Graz, NAWI Graz, Heinrichstrasse 28, 8010 Graz, Austria.

[b] Center for Continuous Flow Synthesis and Processing (CCFLOW), Research Center Pharmaceutical Engineering GmbH (RCPE), Inffeldgasse 13, 8010 Graz, Austria.

[c] Centro de Investigación Lilly S.A., Avda. de la Industria 30, 28108 Alcobendas-Madrid, Spain

[d] TESLA-Analytical Chemistry, Institute of Chemistry, University of Graz, Graz, Austria

*Email: david.cantillo@uni-graz.at

Contents

Materials and methods S2

GC analysis. Sample preparation and calibration data S3

Optimization of the reaction conditions S3

Solvent screening S5

Supporting electrolyte screening S6

Catalyst screening S8

Characterization of the cathode surface S9

Monitoring of the tosylate/halide exchange S10

Experimental procedures and characterization data S13

Supplementary references S24

Copies of NMR spectra S27

Materials and Methods

^{1}H NMR spectra were recorded on a Bruker 300 MHz instrument. ^{13}C NMR spectra were recorded on the same instrument at 75 MHz. Chemical shifts (δ) are expressed in ppm downfield from TMS as internal standard. The letters s, d, t, q, and m are used to indicate singlet, doublet, triplet, quadruplet, and multiplet, respectively. Analytical HPLC analysis was carried out on a C18 reversed-phase (RP) analytical column (150 × 4.6 mm, particle size 5 mm) at 37 °C by using mobile phases A [water/acetonitrile 90:10 (v/v) + 0.1% TFA] and B (acetonitrile + 0.1% TFA) at a flow rate of 1.5 mL/min. The following gradient was applied: linear increase from solution 3% B to 100% B within 10 min. GC–mass spectrometry (MS) analysis was performed on a Shimadzu GCMS-QP2010 SE coupled with a DSQ II (EI, 70 eV). A fused silica capillary column Rtx-5MS column (5% diphenyl, 95% dimethylpolysiloxane, 30 m × 0.25 mm × 0.25 μm) was used. The injector temperature was set at 280 °C. After 1 min at 50 °C, the oven temperature was increased by 25 °C/min to 300 °C and maintained at 300 °C for 3 min. As a carrier gas, helium at 40 cm s^{-1} linear velocity was used. MS conditions were ionization voltage of 70 eV and the acquisition mass range of 50–450 *m*/*z*. Mass spectral libraries (Wiley Registry of Mass Spectral Data 11th Edition, NIST/EPA/NIH Mass Spectral Library 14) were searched with NIST MS Search software. Gas chromatography (GC)–flame ionization detector (FID) chromatography was performed using a Shimadzu GC FID 230 gas chromatograph with a FID. Helium, used as the carrier gas (40 cm s^{-1} linear velocity), goes through a RTX-5MS column (30 m × 0.25 mm ID × 0.25 μm). The injector temperature is set to 280 °C. After 1 min at 50 °C, the column temperature is increased by 25 °C min^{-1} to 300 °C and then held for 4 min at 300 °C. The gases used in the detector for flame ionization are hydrogen and synthetic air (5.0 quality). LA-ICP-MS experiments were performed with an Analyte G2 excimer laser ablation system (Teledyne CETAC Technologies, Omaha, NE, USA) equipped with an aerosol rapid introduction system (ARIS, Teledyne CETAC) and coupled to an an Agilent 8900 Triple Quadrupole ICP-MS/MS instrument (Agilent Technologies, Santa Clara, CA, USA). Helium was used as carrier gas (99.999% purity, Messer Austria GmbH). The LA-ICP-MS system was tuned daily for maximum sensitivity analyzing the reference material NIST 612 "Trace Elements in Glass". The ICP-MS instrument was operated in standard (single quadrupole) mode and tuned to minimize the formation of oxides by monitoring the oxide ratio ($^{232}Th^{16}O^{+}/^{232}Th^{+}$, m/z 248/232 < 1%). Isotope ratios were monitored to confirm the absence of interfering polyatomic species. The laser beam spot size was adjusted to 50 µm and the frequency to 60 Hz generating a fluence of 3.5 J cm^{-2}. Helium was used as carrier gas with a flow rate of 0.55 L min^{-1} (0.30 L min^{-1} cell gas, 0.25 L min^{-1} ablation cup gas). Image construction was perform using the HDIP software v.1.6.6.d44415e5 (Teledyne). Electrode imaging and energy dispersive X-ray (EDX) analysis were performed using a Zeiss Gemini DSM 982 Field Emission Scanning Electron Microscope using built in secondary electron detectors for imaging and a RÖNTEC GmbH M-Series EDX-detector. In addition, element identification was performed using the Röntec-Tools software suite. All electrochemical reactions were carried out in IKA ElectraSyn 2.0 undivided cells (5 mL vials). Aluminum electrodes were cut to standard ElectraSyn 2.0 dimensions from an aluminum sheet (99%, Goodfellow). Electrodes were typically polished using sand paper (3000 grit) before use. All chemicals were purchased from standard vendors and used without further purification.

GC Analysis – Sample Preparation and Calibration

GC samples were prepared by diluting 100 μL aliquots from the crude solutions in 1 mL of ethyl acetate containing 0.1 M biphenyl. The diluted sample was filtered through a celite and sodium sulphate plug before being transferred to a GC sample vial. The vial was capped and the content of the vial was then directly analyzed by with the method described above in the Materials and Methods section. The calibration data for the components of the model reaction is shown in Figure S1.

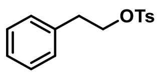

1a

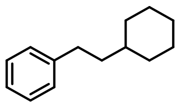

1c

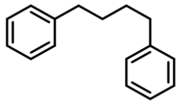

1e

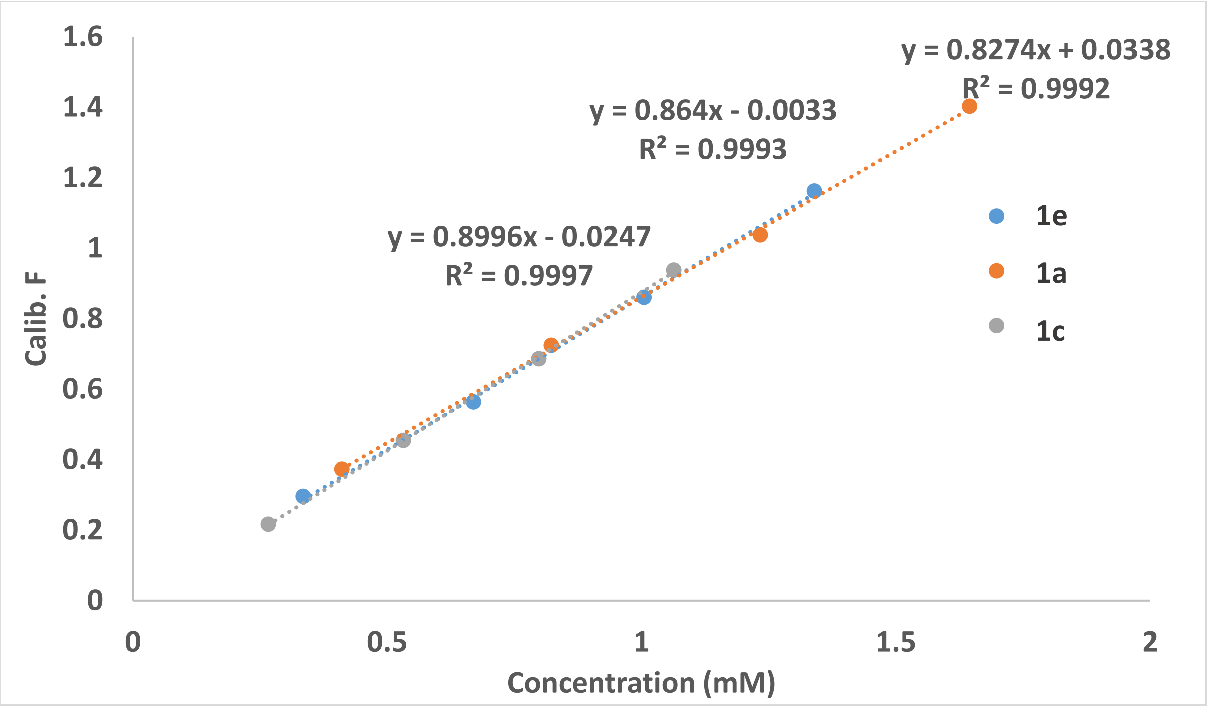

Figure S1. GC calibration of compounds **1a**, **1c**, and **1e**

Optimization of the electrochemical coupling of 2-phenylethyl tosylate (1a) with bromo cyclohexane (1b)

A stock solution of DMA containing $NiBr_2$.dme (0.025 M), 4,4'-di-^{t}Bubpy (0.0375 M) and NaBr (0.1 M) was prepared and purged with argon. The 2-phenylethyl tosylate (**1a**)(0.75 mmol) and bromo cyclohexane (**1b**)(0.975 mmol) were placed in a 5 ml IKA ElectraSyn 2.0 vial and dissolved in 3 mL of the stock solution. The cell was capped and the solution purged with argon for 30 min. The reaction mixture was then electrolyzed a under constant current of 4 mA under stirring (600 rpm) until the desired amount of charge had been passed. After electrolysis, the crude reaction mixtures were analyzed by GC-FID.

Table S1. Optimization of the electrochemical coupling of 2-phenylethyl tosylate (**1a**) with bromo cyclohexane (**1b**).[a]

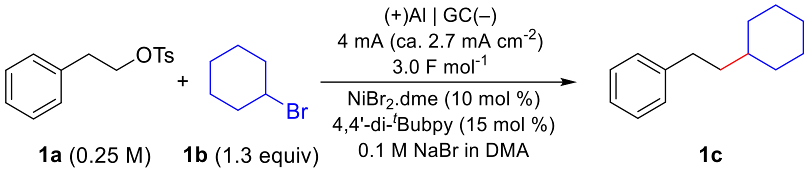

Entry	Deviation from the above	1c (%)[b]
1	--	79
2	10 mA	53
**3**	2 mA	8
4	RVC cathode	66
5	Graphite cathode	74
6	GC (reused)	65
7	Zn anode	24
8	Mg anode	46
9	MeCN as the solvent	3
10	30 mol% ligand	5
11	NaI as supporting electrolyte	46
12	LiBr as supporting electrolyte	51
13	KI as supporting electrolyte	41
14	Bu_4NI as supporting electrolyte	66
15	Bu_4NPF_6 as supporting electrolyte	2
16	Bu_4NBr as supporting electrolyte	51
17	Bu_4NCl as supporting electrolyte	42
18	Et_4NOTs as supporting electrolyte	39
19	$NiCl_2$.dme as the catalyst	50
20	L1	65
21	L2	61
22	L3	45
23	L4	69
24	L5	37
25	Complex 1	6
26	Ni(II) pre-reduction	4

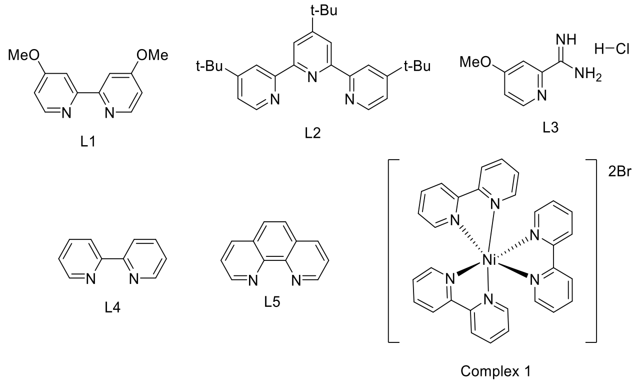

[a] Conditions: 5 mL undivided IKA ElectraSyn 2.0 vial. 3 mL reaction volume, 600 rpm, under Ar. [b] Determined by calibrated GC-FID.

Solvent screening for the electrochemical coupling of 2-phenylethyl tosylate (1a) with bromo cyclohexane (1b)

Following the general conditions for the optimization of the reaction conditions described above, the electrochemical reaction was tested using DMA, NMP, MeCN and 1,4 dioxane as the solvent. After electrolysis, the crude reaction mixtures were analyzed by GC-FID (Table S2).

Table S2. Solvent screening for the electrochemical coupling of 2-phenylethyl tosylate (**1a**) with bromo cyclohexane (**1b**).[a]

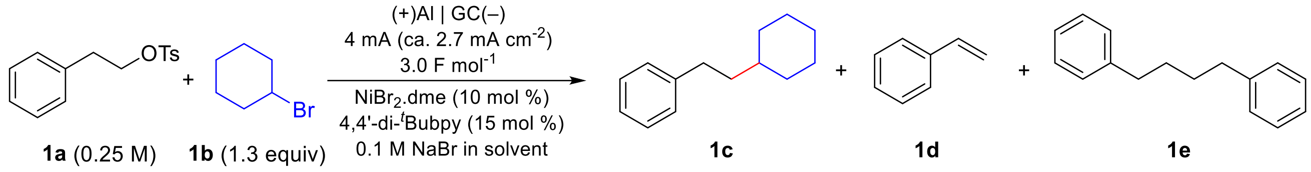

Entry	Solvent	1a (%)[b]	1c (%)[b]	1d (%)[b]	1e (%)[b]
**1**	DMA	< 1	66	0	34
**2**	NMP	6	4	44	3
**3**	MeCN	96	3	0	2
**4**	1,4-dioxane	Maximum voltage, low current			

[a] Conditions: 5 mL undivided IKA ElectraSyn 2.0 vial. 3 mL reaction volume, 600 rpm, under Ar. [b] Determined by GC-FID.

Supporting electrolyte screening for the electrochemical coupling of 2-phenylethyl tosylate (1a) with bromo cyclohexane (1b)

Following the general conditions for the optimization of the reaction conditions described above, the electrochemical reaction was tested using several salts as the supporting electrolyte. After electrolysis, the crude reaction mixtures were analyzed by GC-FID (Table S3).

Table S3. Optimization of the supporting electrolyte for the electrochemical coupling of 2-phenylethyl tosylate (**1a**) with bromo cyclohexane (**1b**)

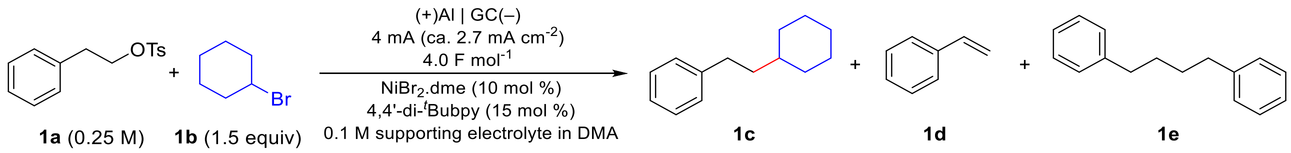

Entry	Electrolyte	1a (%)[b]	1c (%)[b]	1d (%)[b]	1e (%)[b]
1	NaI	< 1	46	18	24
2	KI	3	41	14	29
3	Bu_4NI	< 1	66	7	22
4	Bu_4NPF_6	64	2	< 1	3
5	Bu_4NBr	< 1	51	4	19
6	Bu_4NCl	< 1	42	15	19
7	LiBr	< 1	51	< 1	19
8	Et_4NOTs	15	39	15	13
9	NaBr	< 1	69	1	26

[a] Conditions: 5 mL undivided IKA ElectraSyn 2.0 vial. 3 mL reaction volume, 600 rpm, under Ar. [b] Determined by GC-FID.

Optimization of the loading of the supporting electrolyte loading for the electrochemical coupling of 2-phenylethyl tosylate (1a) with bromo cyclohexane (1b)

Following the general conditions for the optimization of the reaction conditions described above with $NiCl_2$·dme as the catalyst (10 mol%), the electrochemical reaction was tested using variable amounts of NaBr as the supporting electrolyte. After electrolysis, the crude reaction mixtures were analyzed by GC-FID (Figure S2).

Table S4. Optimization of the amount of supporting electrolyte for the electrochemical coupling of 2-phenylethyl tosylate (4a) with bromo cyclohexane (4b)

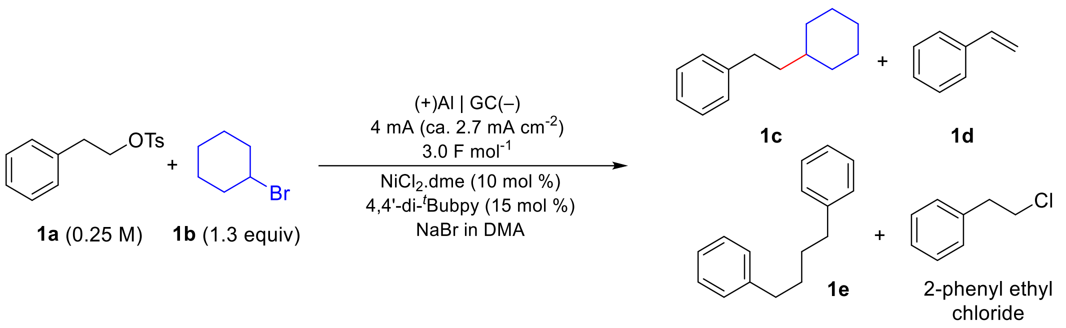

Entry	NaBr (M)	1c (%)[b]	1d (%)[b]	1e (%)[b]	2-phenyl ethyl chloride
1	0.075	63	0	7	30
2	0.1	75	0	8	17
3	0.25	65	0	9	26
4	0.5	57	1	10	32

[a] Conditions: 5 mL undivided IKA ElectraSyn 2.0 vial. 3 mL reaction volume, 600 rpm, under Ar. [b] Determined by GC-FID.

Catalyst screening for the electrochemical coupling of 2-phenylethyl tosylate (1a) with bromo cyclohexane (1b)

Following the general conditions for the optimization of the reaction conditions described above, the electrochemical reaction was tested using several nickel sources as the catalyst. After electrolysis, the crude reaction mixtures were analyzed by GC-FID (Table S4).

Table S5. Optimization of the Nickel catalyst source for the electrochemical coupling of 2-phenylethyl tosylate (**1a**) with bromo cyclohexane (**1b**)

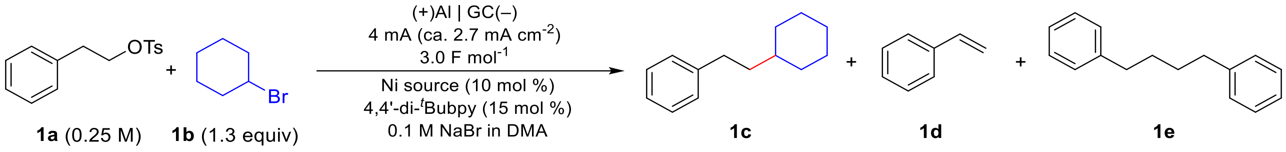

Entry	Ni source	1a (%)	1c (%)	1d (%)	1e (%)
**1**	NiI_2 (5 mol %)	< 1	19	35	11
**2**	NiI_2 (10 mol %)	< 1	39	11	50
**3**	NiI_2 (20 mol %)	< 1	61	< 1	37
**4**	NiI_2 (30 mol %)	< 1	58	< 1	43
**5**	$NiCl_2$.dme (10 mol %)	< 1	50	< 1	23
**6**	$NiBr_2$.dme (10 mol %)	< 1	65	3	33

[a] Conditions: 5 mL undivided IKA ElectraSyn 2.0 vial. 3 mL reaction volume, 600 rpm, under Ar. [b] Determined by GC-FID.

Characterization of the cathode surface after the electrochemical reactions

The glassy carbon electrodes used in this work performed best when used for the first time. Interestingly, reused electrodes typically provided 10%-15% lower yield for the cross-coupling reaction. Reuse of the electrodes for additional experiments did no longer affect the reaction outcome, pointing to a stable modification of the carbon surface when the new electrode was used for the first time. To shed light into this material modification, the surface of a used electrode was analyzed by SEM-EDX and LA-ICP-MS (Figure S3). SEM-EDX analysis pointed to coating of the carbon surface with either bromine or aluminum (Figure S3, top). LA-ICP-MS revealed accumulation of bromine on the surface of the electrode that had been immersed in the reaction mixture during electrolysis (Figure S3, bottom).

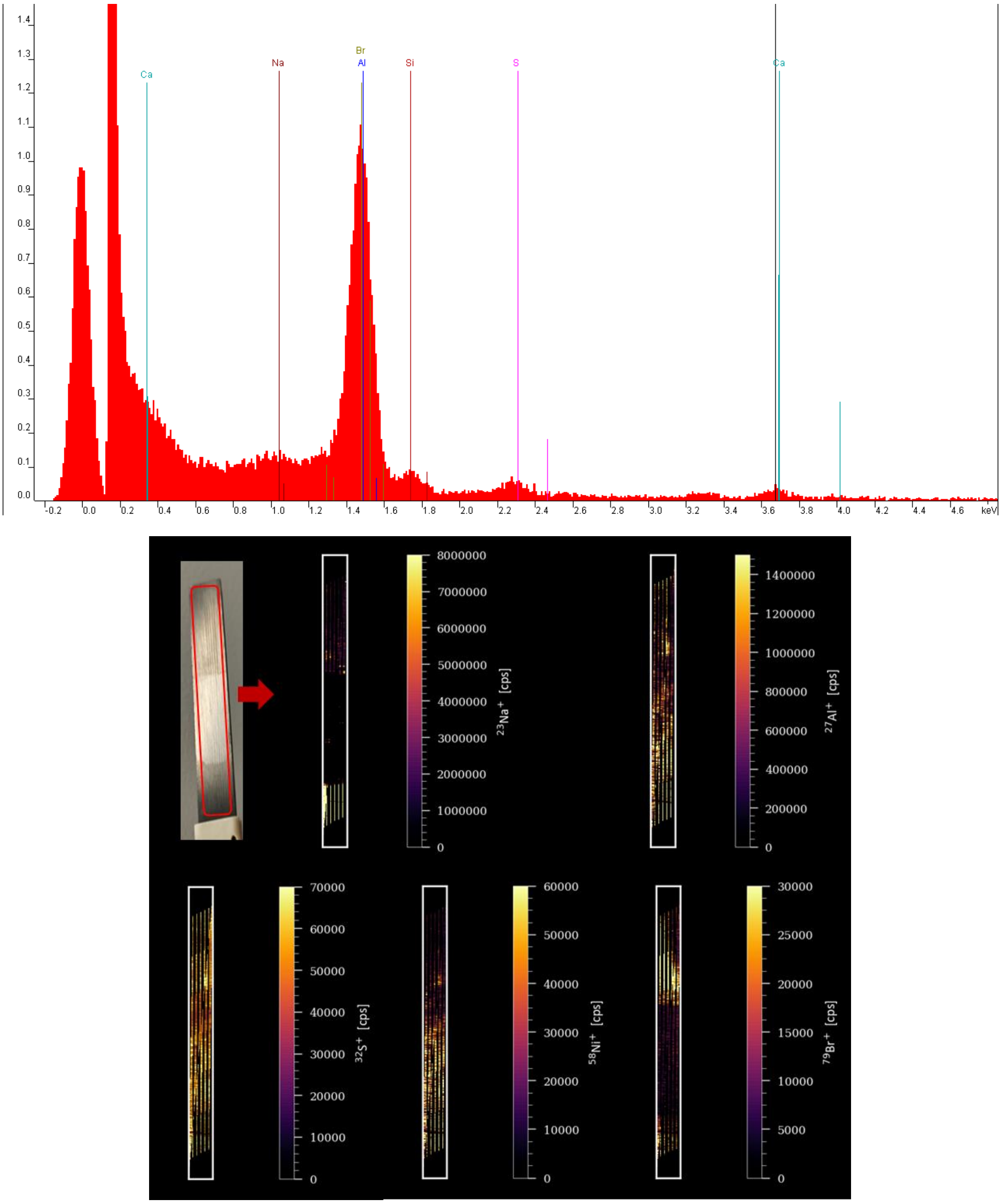

Figure S2. SEM-EDX an area of the surface on a used glassy carbon electrode (top) and longitudinal LA-ICP-MS profile of the same electrode (bottom).

Monitoring of the tosylate/halide exchange rates in DMA for various alkyl tosylates.

A stock solution of DMA that contains $NiBr_2$.dme catalyst (0.025 M), and NaBr supporting electrolyte (0.35 M) was prepared and purged with argon. The corresponding amounts of 1) 2-phenyl tosylate, 2) octyl tosylate, 3) 1-phenyl-2-propyl tosylate, 4) 4-terbutyl-1-phenyl tosylate were placed in a 4 ml glass vial followed by 3 ml of the DMA stock solution were added so that the concentration of the tosylate is 0.25 M. The vials were capped and the reaction mixture was stirred and purged with argon for 30 minutes. The reaction mixture was allowed to progress at room temperature while timely samples were collected and analyzed by GC-FID following the procedure above. In a separate experiment, the aluminum-containing precipitate from a model cross coupling reaction described in Table S1, entry 1 was filtered and added to the tosylate-bromide substitution experiment to investigate the effect of the aluminum salt on the kinetics.

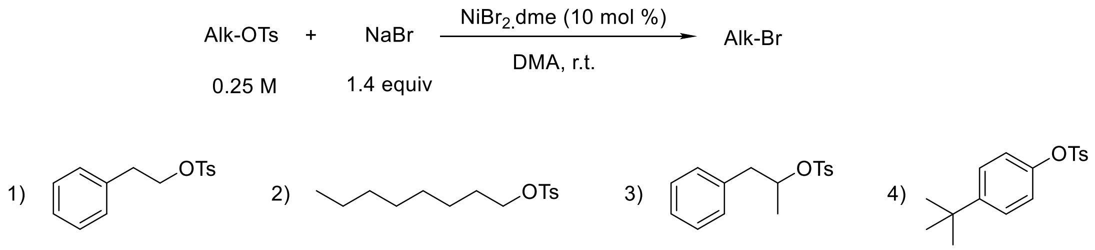

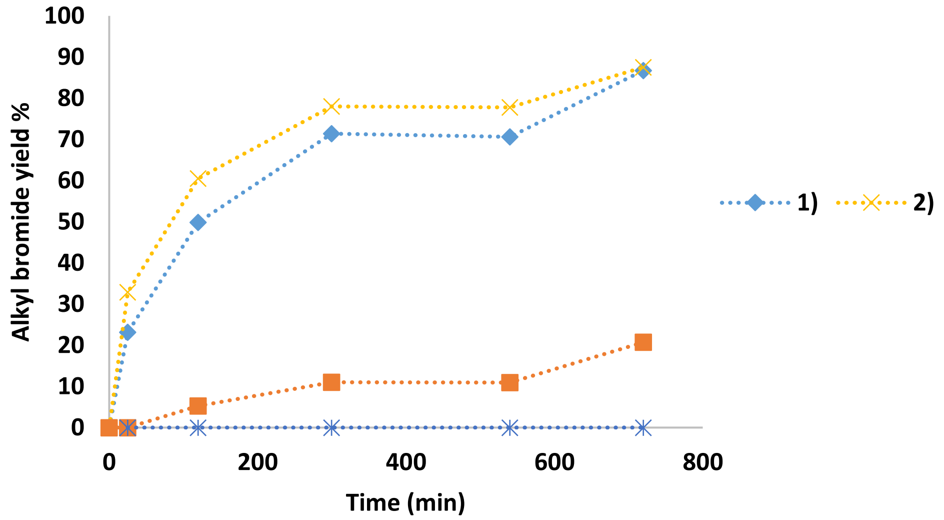

Figure S3. Alkyl tosylate-bromide substitution temporal progress with NaBr in DMA in presence of NiBr2.dme catalyst for 1) 2-phenyl tosylate, 2) octyl tosylate, 3) 1-phenyl-2-propyl tosylate, 4) 4-terbutyl-1-phenyl tosylate.

Optimization of the concentration of supporting electrolyte for the electrochemical coupling of octyl tosylate (23a) with bromo cyclohexane (1b)

The amount of supporting electrolyte was reoptimized for the cross-coupling between octyl tosylate and cyclohexyl bromide. Thus, following the general conditions for the optimization of the reaction conditions described above with octyl tosylate as the substrate and $NiCl_2$·dme as the catalyst (10 mol%), the electrochemical reaction was tested using variable amounts of NaBr as the supporting electrolyte. After electrolysis, the crude reaction mixtures were analyzed by GC-FID (Table S5).

Table S6. Optimization of the amount of supporting electrolyte for the electrochemical coupling of octyl tosylate (**23a**) with bromo cyclohexane (**1b**)

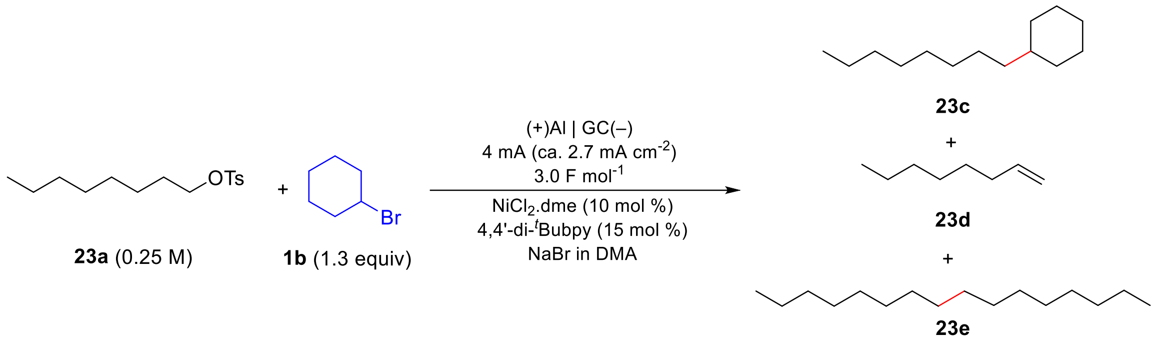

Entry	NaBr (M)	23c (%)[b]	23d (%)[b]	23e (%)[b]
1	0.05	25	29	11
2	0.75	56	30	14
	0.1	50	32	18
3	0.35	35	39	26
4	0.5	32	34	29

[a] Conditions: 5 mL undivided IKA ElectraSyn 2.0 vial. 3 mL reaction volume, 600 rpm, under Ar. [b] Determined by GC-FID.

Optimization of the amount of alkyl bromide for the electrochemical coupling of octyl tosylate (23a) with bromo cyclohexane (1b)

The amount of cyclohexyl bromide was reoptimized for the cross-coupling between octyl tosylate and cyclohexyl bromide. Thus, following the general conditions for the optimization of the reaction conditions described above with octyl tosylate as the substrate, the electrochemical reaction was tested using variable amounts of octyl bromide. After electrolysis, the crude reaction mixtures were analyzed by GC-FID (Table S6).

Table S7. Optimization of the amount of alkyl bromide for the electrochemical coupling of octyl tosylate (**23a**) with bromo cyclohexane (**1b**)

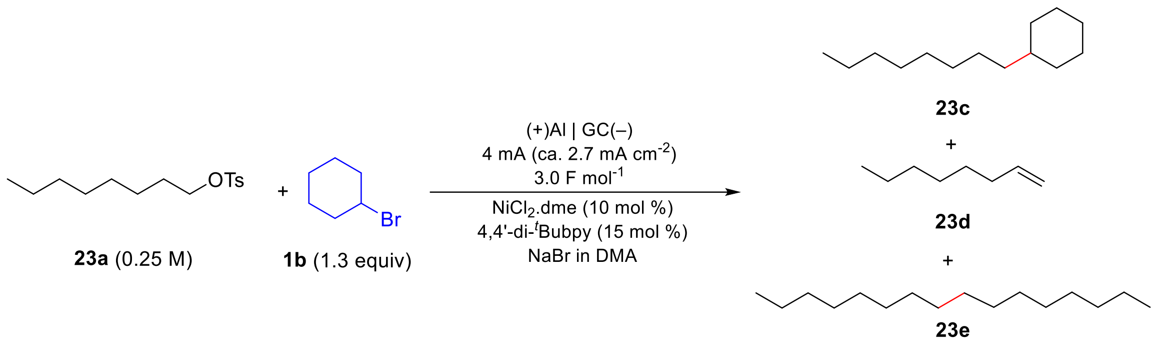

Entry	1b (equiv)	23c (%)[b]	23d (%)[b]	23e (%)[b]
1	1.2	39	44	18
2	1.4	43	37	13
3	1.6	46	41	13
4	2.0	53	31	16

[a] Conditions: 5 mL undivided IKA ElectraSyn 2.0 vial. 3 mL reaction volume, 600 rpm, under Ar. [b] Determined by GC-FID.

Experimental procedures and compound characterization

Synthesis of starting materials

Synthesis of alkyl tosylates. The synthesis of the alkyl tosylates employed as reactants was carried out using either tosyl chloride following method (1) or tosyl anhydride following method (2). **Method (1):** alkyl tosylates were prepared following the procedure reported by Komeyama et al.[1] In a glass vial, N,N-dimethylaminopyridine (DMAP) (2 mol%) and tosyl chloride (TsCl) (1.2 equiv) were dissolved in 5.5 mL of anhydrous DCM. The reaction mixture was cooled in an ice bath. Then, the corresponding alcogol (2.5 mmol) was added followed by 1.3 equiv of Et_3N. The solution was stirred at 600 rpm at room temperature for 24 h. Then, the reaction mixture was diluted with DCM and washed with aqueous $NaHCO_3$ and brine. The organic layer was dried over Na_2SO_4 and the solvent evaporated. The crude residue was purified by column chromatography. **Method (2):** alkyl tosylates were prepared following the method by by Comagic et al.[2] In a glass vial, $Yb(OTf)_3$ (1 mol%) and tosyl anhydride (Ts_2O) (1.3 equiv) were dissolved in 20 mL of anhydrous DCM. The solution was purged with argon for 30 min. Then, the corresponding alcohol was added (2 mmol) and the reaction was stirred at 600 rpm for 24 h. The crude reaction mixture was diluted with DCM and then washed with aqueous 2 M K_2HPO_4 and brine. The organic layer was dried over $MgSO_4$, the solvent evaporated under reduced pressure and the residue purified by column chromatography.

Scheme S1. Alkyl tosylates prepared using Method A. Isolated yields are shown.

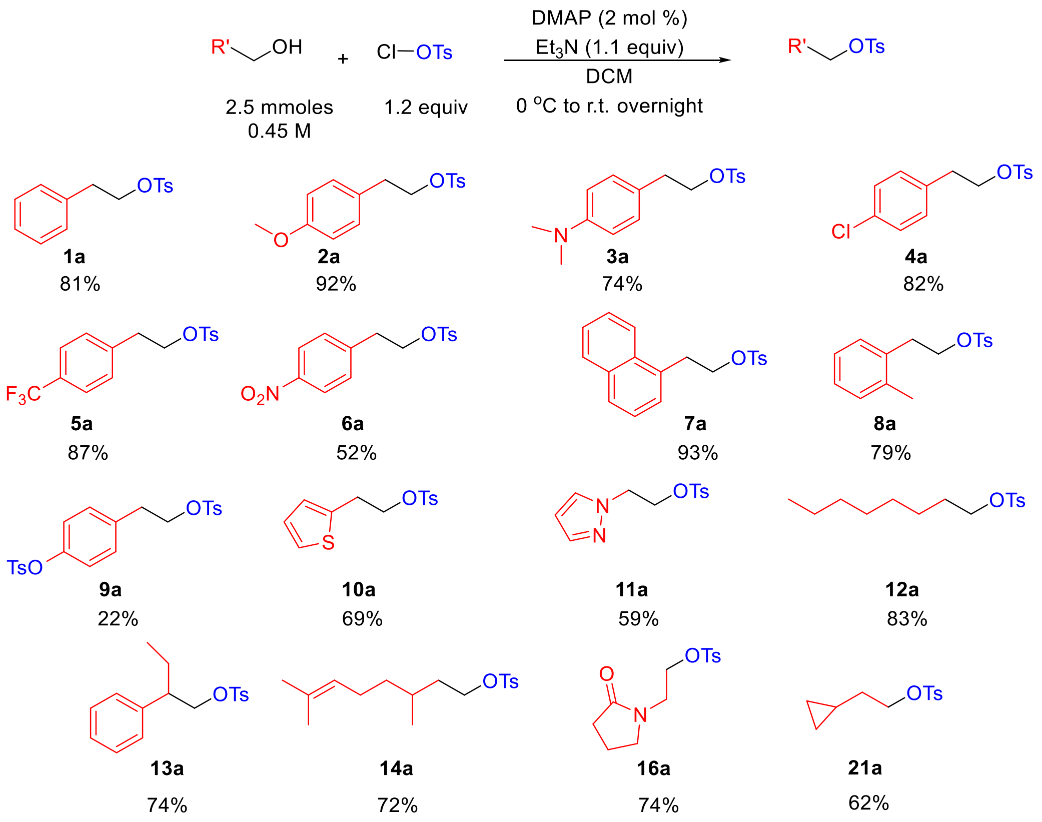

Scheme S2. Alkyl tosylates prepared using Method B. Isolated yields are shown.

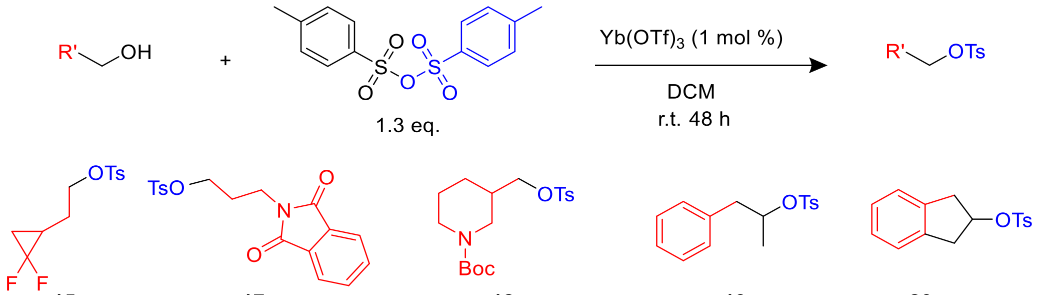

15a	17a	18a	19a	20a
61%	84%	49%	85%	82%

Phenethyl 4-methylbenzenesulfonate (1a)[3]

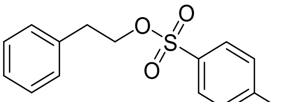

1a

Following the general Method A, the title compound was obtained as a white crystalline solid (559 mg, 81%).

^{1}H NMR (300 MHz, Chloroform-*d*) δ 7.75 – 7.61 (m, 2H), 7.30 – 7.18 (m, 5H), 7.09 (dd, *J* = 7.6, 2.0 Hz, 2H), 4.19 (t, *J* = 7.1 Hz, 2H), 2.93 (t, *J* = 7.1 Hz, 2H), 2.41 (s, 3H).

GC-MS-EI analysis: m/z 276 (1%), 155 (5%), 118 (100%), 105 (45%), 91 (40%)

4-Methoxyphenethyl 4-methylbenzenesulfonate (2a)[4]

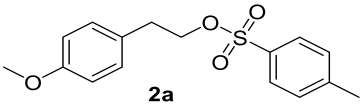

2a

Following the general Method A, the title compound was obtained as a white solid (704 mg, 92%).

^{1}H NMR (300 MHz, Chloroform-*d*) δ 7.67 (d, *J* = 8.4 Hz, 2H), 7.32 – 7.24 (m, 4H), 7.05 – 6.93 (m, 2H), 6.82 – 6.69 (m, 2H), 4.14 (t, *J* = 7.1 Hz, 2H), 3.76 (s, 3H), 2.87 (t, *J* = 7.1 Hz, 2H), 2.41 (s, 3H).

GC-MS-EI analysis: m/z 306 (3%), 155 (1%), 134 (100%), 121 (52%), 91 (25%)

4-(Dimethylamino)phenethyl 4-methylbenzenesulfonate (3a)[5]

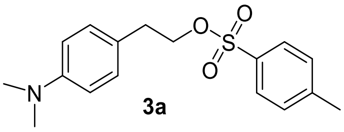

3a

Following the general Method A, the title compound was obtained as a red solid (590 mg. 75%).

^{1}H NMR (300 MHz, Chloroform-*d*) δ 8.01 – 7.56 (m, 2H), 7.38 – 7.13 (m, 2H), 6.96 (d, *J* = 8.5 Hz, 2H), 6.61 (d, *J* = 8.3 Hz, 2H), 4.12 (t, *J* = 7.4 Hz, 2H), 2.89 (s, 2H), 2.84 (t, *J* = 7.4 Hz, 8H), 2.41 (s, 3H).

GC-MS-EI analysis: m/z 319 (3%), 147 (62%), 118 (11%), 91 (18%)

4-Chlorophenethyl 4-methylbenzenesulfonate (4a)[6]

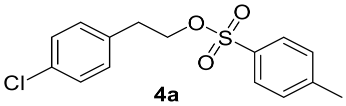

4a

Following the general Method A, the title compound was obtained as a white solid (636 mg, 82%).

^{1}H NMR (300 MHz, Chloroform-*d*) δ 7.63 (d, *J* = 8.3 Hz, 2H), 7.29 – 7.09 (m, 6H), 7.00 (d, *J* = 8.4 Hz, 2H), 4.17 (t, *J* = 6.7 Hz, 2H), 2.89 (t, *J* = 6.7 Hz, 2H), 2.42 (s, 3H).

GC-MS-EI analysis: m/z 310 (1%), 155 (10%), 138 (100%), 125 (35%), 91 (49%)

4-(Trifluoromethyl)phenethyl 4-methylbenzenesulfonate (5a)[7]

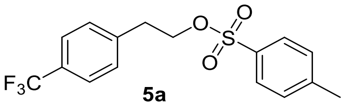

5a

Following the general Method A, the title compound was obtained as a white solid (727 mg, 87%).

^{1}H NMR (300 MHz, Chloroform-*d*) δ 7.61 (d, *J* = 8.4 Hz, 2H), 7.46 (d, *J* = 8.0 Hz, 2H), 7.27 – 6.99 (m, 9H), 4.22 (t, *J* = 6.6 Hz, 2H), 2.98 (t, *J* = 6.6 Hz, 2H), 2.40 (s, 3H).

GC-MS-EI analysis: m/z 172 (100%), 159 (29%), 155 (30%), 91 (75%)

4-Nitrophenethyl 4-methylbenzenesulfonate (6a)[8]

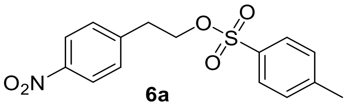

6a

Following the general Method A, the title compound was obtained as a brown oil (418 mg, 52%).

^{1}H NMR (300 MHz, Chloroform-*d*) δ 8.07 (d, *J* = 8.7 Hz, 2H), 7.63 (d, *J* = 8.3 Hz, 2H), 7.30 – 7.19 (m, 8H), 4.26 (t, *J* = 6.4 Hz, 2H), 3.04 (t, *J* = 6.4 Hz, 2H), 2.41 (s, 3H).

GC-MS-EI analysis: m/z 321 (25%), 155 (40%), 149 (80%), 119 (20%), 91 (100%)

2-(Naphthalen-1-yl)ethyl 4-methylbenzenesulfonate (7a)[9]

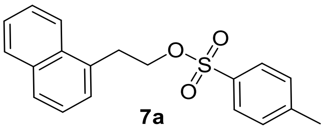

7a

Following the general Method A, the title compound was obtained as a white solid (758 mg, 93%).

^{1}H NMR (300 MHz, Chloroform-*d*) δ 7.94 – 6.98 (m, 11H), 4.31 (t, *J* = 7.4 Hz, 2H), 3.41 (t, *J* = 7.3 Hz, 2H), 2.36 (s, 3H).

GC-MS-EI analysis: m/z 326 (10%), 154 (10%), 141 (52%), 115 (25%), 91 (30%)

2-Methylphenethyl 4-methylbenzenesulfonate (8a)[10]

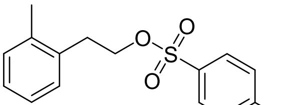

8a

Following the general Method A, the title compound was obtained as a colorless oil (572 mg, 79%).

^{1}H NMR (300 MHz, Chloroform-*d*) δ 7.68 (d, *J* = 8.3 Hz, 2H), 7.32 – 7.25 (m, 2H), 7.17 – 6.94 (m, 3H), 4.14 (t, *J* = 7.5 Hz, 2H), 2.95 (t, *J* = 7.4 Hz, 2H), 2.41 (s, 3H), 2.20 (s, 3H).

GC-MS-EI analysis: m/z 155 (10%), 118 (100%), 105 (40%), 91 (40%)

4-(2-(Tosyloxy)ethyl)phenyl 4-methylbenzenesulfonate (9a)[11]

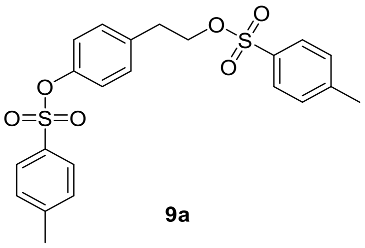

9a

Following the general Method A, the title compound was obtained as a white solid (245 mg, 22%).

^{1}H NMR (300 MHz, Chloroform-*d*) δ 7.84 (d, *J* = 8.5 Hz, 2H), 7.66 (dd, *J* = 11.6, 8.4 Hz, 4H), 7.44 – 7.26 (m, 6H), 7.00 (d, *J* = 8.6 Hz, 2H), 6.84 (d, *J* = 8.6 Hz, 2H), 2.89 (t, *J* = 6.8 Hz, 2H), 2.46 (s, 3H), 2.42 (s, 3H).

2-(Thiophen-2-yl)ethyl 4-methylbenzenesulfonate (10a)[12]

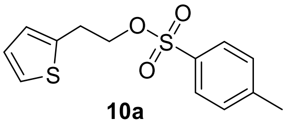

10a

Following the general Method A, the title compound was obtained as a yellow solid (487 mg, 22%).

^{1}H NMR (300 MHz, Chloroform-*d*) δ 7.83 – 7.67 (m, 2H), 7.34 (d, *J* = 8.0 Hz, 2H), 7.18 (ddd, *J* = 11.1, 5.1, 1.2 Hz, 1H), 6.96 – 6.88 (m, 1H), 6.86 – 6.78 (m, 1H), 4.24 (t, *J* = 6.9 Hz, 2H), 3.20 (td, *J* = 6.9, 0.9 Hz, 2H), 2.47 (s, 3H).

GC-MS-EI analysis: m/z 155 (7%), 110 (100%), 91 (30%), 65 (18%)

2-(1H-Pyrazol-1-yl)ethyl 4-methylbenzenesulfonate (11a)[13]

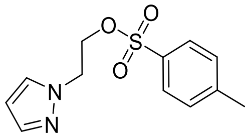

11a

Following the general Method A, the title compound was obtained as a yellow oil at (392 mg, 59%).

^{1}H NMR (300 MHz, Chloroform-*d*) δ 7.62 (d, *J* = 8.4 Hz, 2H), 7.48 – 7.39 (m, 2H), 7.31 – 7.11 (m, 2H), 6.19 (t, *J* = 2.1 Hz, 1H), 4.49 – 4.09 (m, 4H), 2.41 (s, 3H).

GC-MS-EI analysis: m/z 266 (1%), 172 (35%), 111 (20%), 94 (85%), 81 (100%)

Octyl 4-methylbenzenesulfonate (12a)[14]

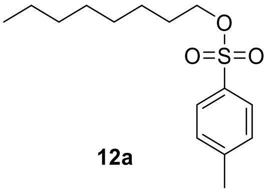

12a

Following the general Method A, the title compound was obtained as a colorless oil (589 mg, 83%).

^{1}H NMR (300 MHz, Chloroform-*d*) δ 7.83 – 7.72 (m, 2H), 7.37 – 7.29 (m, 2H), 3.99 (t, *J* = 6.5 Hz, 2H), 2.43 (s, 3H), 1.60 (dt, *J* = 8.1, 6.5 Hz, 2H), 1.34 – 1.15 (m, 10H), 0.90 – 0.78 (m, 3H).

GC-MS-EI analysis: m/z 284 (1%), 173 (77%), 155 (40%), 112 (48%), 91 (100%)

2-Phenylbutyl 4-methylbenzenesulfonate (13a)[15]

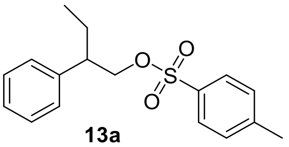

13a

Following the general Method A, the title compound was obtained as a white solid (562 mg, 74%).

^{1}H NMR (300 MHz, Chloroform-*d*) δ 7.62 (d, *J* = 8.3 Hz, 2H), 7.35 – 7.18 (m, 5H), 7.04 (dd, *J* = 7.7, 1.9 Hz, 2H), 4.37 – 3.92 (m, 2H), 2.78 (dq, *J* = 9.5, 6.7 Hz, 1H), 2.41 (s, 3H), 1.94 – 1.37 (m, 2H), 0.75 (t, *J* = 7.4 Hz, 3H).

GC-MS-EI analysis: m/z 304 (1%), 155 (12%), 132 (75%), 117 (40%), 91 (100%)

3,7-Dimethyloct-6-en-1-yl 4-methylbenzenesulfonate (14a)[16]

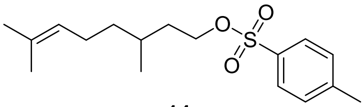

14a

Following the general Method A, the title compound was obtained as a colourless oil (558 mg, 72%).

^{1}H NMR (300 MHz, Chloroform-*d*) δ 7.85 – 7.73 (m, 2H), 7.40 – 7.28 (m, 2H), 2.43 (s, 3H), 1.90 (dq, *J* = 16.8, 7.6 Hz, 2H), 1.72 – 1.60 (m, 4H), 1.49 – 1.35 (m, 1H), 1.20 (ddd, *J* = 9.4, 6.5, 5.2 Hz, 1H).

GC-MS-EI analysis: m/z 138 (42%), 123 (52%), 80 (95%), 81 (100%)

2-(2,2-Difluorocyclopropyl)ethyl 4-methylbenzenesulfonate (15a)[17]

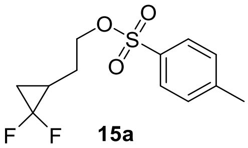

15a

Following the general Method B, the title compound was obtained as a colourless oil (558 mg, 72%).

^{1}H NMR (300 MHz, Chloroform-*d*) δ 7.78 (d, *J* = 8.3 Hz, 2H), 7.34 (d, *J* = 8.0 Hz, 2H), 4.07 (t, *J* = 6.2 Hz, 2H), 2.44 (s, 3H), 1.93 – 1.79 (m, 1H), 1.79 – 1.66 (m, 0H), 1.44 – 1.31 (m, 1H), 0.91 (dtd, *J* = 12.9, 7.4, 3.5 Hz, 1H).

GC-MS-EI analysis: m/z 276 (25%), 173 (10%), 155 (40%), 104 (80%), 91 (100%)

2-(2-Oxopyrrolidin-1-yl)ethyl 4-methylbenzenesulfonate (16a)[18]

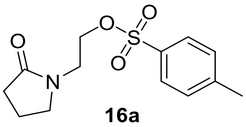

16a

Following the general Method A, the title compound was obtained as a white solid (431 mg, 61%).

^{1}H NMR (300 MHz, Chloroform-*d*) δ 7.76 (d, *J* = 8.2 Hz, 2H), 7.31 (d, *J* = 8.1 Hz, 2H), 4.08 (t, *J* = 6.2 Hz, 2H), 3.72 (t, *J* = 7.0 Hz, 2H), 2.42 (s, 3H), 2.14 – 1.88 (m, 2H).

GC-MS-EI analysis: m/z 283 (1%), 128 (38%), 111 (35%), 98 (100%), 91 (10%)

3-(1,3-Dioxoisoindolin-2-yl)propyl 4-methylbenzenesulfonate (17a)[19]

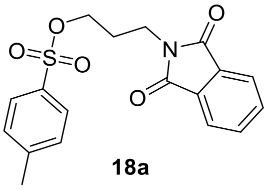

18a

Following the general Method A, the title compound was obtained as a white solid (431 mg, 61%).

^{1}H NMR (300 MHz, Chloroform-*d*) δ 8.10 – 7.57 (m, 6H), 7.30 (d, *J* = 8.1 Hz, 2H), 4.08 (t, *J* = 6.2 Hz, 2H), 3.72 (t, *J* = 7.0 Hz, 2H), 2.42 (s, 3H), 2.04 (p, *J* = 6.5 Hz, 2H).

GC-MS-EI analysis: m/z 359 (1%), 188 (100%), 160 (90%), 130 (30%), 104 (25%)

tert-Butyl 3-((tosyloxy)methyl)piperidine-1-carboxylate (18a)[20]

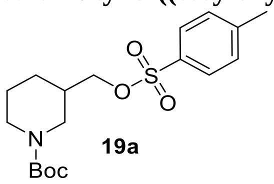

19a

Following the general Method B, the title compound was obtained as a white solid (452 mg, 49%).
^{1}H NMR (400 MHz, CHLOROFORM-*d*) δ ppm 1.17 - 1.41 (m, 2 H) 1.43 (s, 9 H) 1.56 - 1.69 (m, 2 H) 1.79 - 1.91 (m, 1 H) 2.45 (s, 3 H) 2.54 - 2.87 (m, 2 H) 3.74 - 3.86 (m, 2 H) 3.89 (dd, J=6.18, 1.60 Hz, 2 H) 7.35 (d, J=7.79 Hz, 2 H) 7.78 (d, J=8.24 Hz, 2 H)
GC-MS-EI analysis: m/z 369 (1%), 350 (40%), 321 (20%), 295 (100%), 282 (60%)

1-Phenylpropan-2-yl 4-methylbenzenesulfonate (19a)[21]

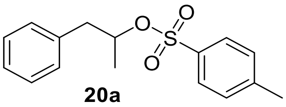

20a

Following the general Method B, the title compound was obtained as a white solid (616 mg, 85%).

^{1}H NMR (300 MHz, Chloroform-*d*) δ 7.60 (d, *J* = 8.3 Hz, 2H), 7.24 – 7.07 (m, 5H), 7.11 – 6.73 (m, 2H), 4.72 (q, *J* = 6.4 Hz, 1H), 2.90 (dd, *J* = 13.8, 6.6 Hz, 1H), 2.75 (dd, *J* = 13.8, 6.5 Hz, 1H), 2.39 (s, 3H), 1.28 (d, *J* = 6.2 Hz, 3H).

GC-MS-EI analysis: m/z 369 (1%), 350 (40%), 321 (20%), 295 (100%), 282 (60%)

2,3-Dihydro-1H-inden-2-yl 4-methylbenzenesulfonate (20a)[22]

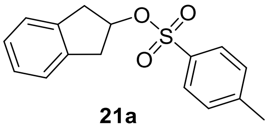

21a

Following the general Method B, the title compound was obtained as a white solid (612 mg, 85%).

^{1}H NMR (300 MHz, Chloroform-*d*) δ 7.82 (dd, *J* = 15.4, 8.4 Hz, 3H), 7.42 – 7.28 (m, 2H), 7.15 (s, 1H), 5.28 (s, 1H), 3.41 – 3.03 (m, 1H), 2.46 (s, 3H).

2-Cyclopropylethyl 4-methylbenzenesulfonate (21a)[23]

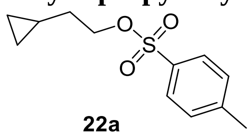

22a

Following the general Method A, the title compound was obtained as a white solid (372 mg, 62%).

^{1}H NMR (300 MHz, Chloroform-*d*) δ 7.82 – 7.66 (m, 2H), 7.32 (d, *J* = 8.1 Hz, 2H), 4.06 (t, *J* = 6.6 Hz, 2H), 2.42 (s, 4H), 1.51 (q, *J* = 6.8 Hz, 2H), 0.78 – 0.52 (m, 1H), 0.48 – 0.19 (m, 2H), -0.01 (dd, *J* = 4.8, 1.4 Hz, 2H).

GC-MS-EI analysis: 240 (0%), 172 (40%), 171 (20%). 155 (10%), 134 (10%), 111 (20%), 82 (100%)

Electrochemical nickel-catalyzed cross-coupling of alkyl tosylates with alkyl bromides

A stock solution of DMA containing $NiBr_2$.dme (0.025 M), 4,4'-di-^{t}Bubpy (0.0375 M) and NaBr (0.1 M) was purged with argon for 30 min. The corresponding amounts of the alkyl tosylate and alkyl bromide were placed in a 5 ml IKA ElectraSyn 2.0 vial, followed by 3 mL of the aforementioned DMA stock solution. The cell cap was equipped with a glassy carbon cathode and an aluminum anode. The cell was capped and the reaction mixture was stirred and purged with argon for 30 minutes before the electrolysis was started. Then, the solution was electrolyzed under a constant current of 4 mA (2.7 mA/cm^2) under argon atmosphere while stirring it at 600 rpm. Electrolysis was continues until 3 F/mol of charge had been passed. After electrolysis, the crude reaction mixture was diluted with ethyl acetate and washed five times with an aqueous 20 wt% sodium citrate solution. The organic layer was dried over Na_2SO_4, the solvent evaporated, and the crude residue purified using column chromatography with petroleum ether/ethyl acetate as the eluent.

(2-Cyclohexylethyl)benzene (1c)[24]

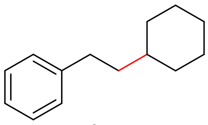

1c

Following the general procedure, the title compound was isolated as a colorless oil (110 mg, 78%).

^{1}H NMR (300 MHz, Chloroform-*d*) δ 7.26 (d, *J* = 7.9 Hz, 2H), 7.16 (d, *J* = 7.2 Hz, 2H), 2.84 – 2.46 (m, 2H), 1.90 – 1.59 (m, 2H), 1.55 – 1.36 (m, 2H), 1.36 – 1.06 (m, 4H), 1.02 – 0.65 (m, 2H).
^{13}C NMR (75 MHz, $CDCl_3$) δ 143.3, 128.4, 128.3, 125.5, 39.5, 37.4, 33.4, 33.3, 26.8, 26.4.

GC-MS-EI analysis: m/z 188 (12%), 105 (1%), 97 (10%), 92 (100%), 55 (35%)

(2-Cyclopentylethyl)benzene (2c)[25]

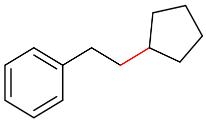

2c

Following the general procedure, the title compound was isolated as a colorless oil (93 mg, 71%).

^{1}H NMR (300 MHz, Chloroform-*d*) δ 7.31 – 7.21 (m, 3H), 7.19 – 7.08 (m, 2H), 2.77 – 2.37 (m, 2H), 1.87 – 1.70 (m, 3H), 1.66 – 1.42 (m, 6H), 1.29 – 0.98 (m, 2H).
^{13}C NMR (126 MHz, Chloroform-*d*) δ 143.3, 128.4, 128.3, 125.5, 39.5, 37.4, 33.4, 33.3, 26.75, 26.39.

GC-MS-EI analysis: m/z 174 (11%), 117 (3%), 105 (5%), 92 (100%), 55 (35%)

Octylbenzene (3c)[26]

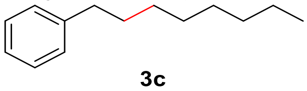

3c

Following the general procedure, the title compound was isolated as a colorless oil (107 mg, 75%).

^{1}H NMR (300 MHz, Chloroform-*d*) δ 7.38 – 7.21 (m, 2H), 7.20 – 6.99 (m, 2H), 2.77 – 2.41 (m, 2H), 1.84 – 1.52 (m, 2H), 1.33 – 1.15 (m, 10H), 0.96 – 0.72 (m, 3H).
^{13}C NMR (126 MHz, Chloroform-*d*) δ 143.0, 128.4, 128.2, 125.5, 36.0, 31.9, 31.6, 29.5, 29.4, 29.3, 22.7, 14.1.

GC-MS-EI analysis: m/z 190 (36%), 113 (12%), 105 (10%), 92 (100%), 57 (20%)

(4,4-dimethylpentyl)benzene (4c)[27]

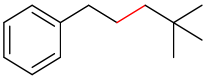

4c

Following the general procedure, the title compound was isolated as a colorless oil (96 mg, 73%).

^{1}H NMR (300 MHz, Chloroform-*d*) δ 7.49 – 6.87 (m, 5H), 2.55 (t, *J* = 7.8 Hz, 2H), 1.70 – 1.54 (m, 2H), 1.32 – 1.03 (m, 2H), 0.85 (s, 9H).
^{13}C NMR (126 MHz, Chloroform-*d*) δ 143.0, 128.4, 128.2, 125.6, 44.0, 36.9, 30.3, 29.4, 26.7.

GC-MS-EI analysis: m/z 176 (12%), 161 (1%), 120 (25%), 105 (12%), 91 (60%), 57 (100%)

(4-Cyclopropylbutyl)benzene (5c)[28]

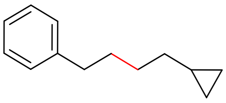

5c

Following the general procedure, the title compound was isolated as a colorless oil (97 mg, 74%).

^{1}H NMR (300 MHz, Chloroform-*d*) δ 7.31 – 7.20 (m, 2H), 7.17 (d, *J* = 7.1 Hz, 3H), 2.77 – 2.37 (m, 2H), 1.63 (tt, *J* = 9.2, 6.8 Hz, 2H), 1.50 – 1.33 (m, 2H), 1.25 – 1.11 (m, 2H), 0.63 (ddtd, *J* = 11.6, 9.5, 7.2, 4.9 Hz, 1H), 0.43 – 0.25 (m, 2H), 0.10 – -0.18 (m, 2H), -0.05 (m, 2H).
^{13}C NMR (75 MHz, Chloroform-*d*) δ 142.9, 128.4, 128.2, 125.6, 36.1, 34.6, 31.4, 29.4, 10.8, 4.4.

GC-MS-EI analysis: m/z 174 (10%), 145 (9%), 117 (35%), 104 (90%), 91 (100%)

6-Phenylhexanenitrile (9c)[29]

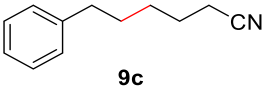

9c

Following the general procedure, the title compound was isolated as a yellow oil (51 mg, 39%).

^{1}H NMR (300 MHz, Chloroform-*d*) δ 7.37 – 7.22 (m, 2H), 7.21 – 7.06 (m, 3H), 2.68 – 2.51 (m, 2H), 2.31 (t, *J* = 7.1 Hz, 2H), 1.79 – 1.58 (m, 4H), 1.52 – 1.38 (m, 2H).
^{13}C NMR (126 MHz, Chloroform-*d*) δ 142.0, 128.4, 128.4, 125.9, 119.7, 35.6, 30.6, 28.3, 25.3, 17.1.

GC-MS-EI analysis: m/z 173 (14%), 144 (10%), 130 (11%), 105 (6%), 91 (100%)

1-(2-Cyclohexylethyl)-4-methoxybenzene (11c)[30]

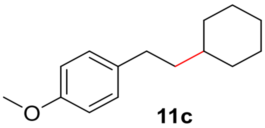

11c

Following the general procedure, the title compound was isolated as a colorless oil (105 mg, 64%).

^{1}H NMR (500 MHz, Chloroform-*d*) δ 7.14 – 7.00 (m, 2H), 6.85 – 6.71 (m, 2H), 3.77 (d, *J* = 1.0 Hz, 3H), 2.67 – 2.38 (m, 2H), 1.91 – 1.58 (m, 5H), 1.51 – 1.34 (m, 2H), 1.30 – 1.06 (m, 4H), 0.96 – 0.72 (m, 2H).
^{13}C NMR (126 MHz, Chloroform-*d*) δ 157.6, 135.4, 129.2, 113.7, 55.3, 39.7, 37.3, 33.4, 32.3, 26.7, 26.4.

GC-MS-EI analysis: m/z 218 (25%), 121 (100%), 108 (10%), 91 (7.5%), 77 (7.5%)

4-(4-Methoxyphenethyl)tetrahydro-2H-pyran (12c)

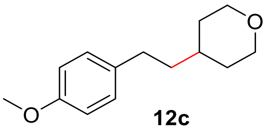

12c

Following the general procedure, the title compound was isolated as a colorless oil (107 mg, 65%).

^{1}H NMR (500 MHz, Chloroform-*d*) δ 7.07 (d, *J* = 8.6 Hz, 2H), 6.81 (d, *J* = 8.6 Hz, 2H), 4.07 – 3.88 (m, 2H), 3.77 (s, 3H), 3.34 (td, *J* = 11.8, 2.1 Hz, 2H), 2.61 – 2.48 (m, 2H), 1.74 – 1.59 (m, 2H), 1.56 – 1.44 (m, 3H), 1.37 – 1.25 (m, 2H).
^{13}C NMR (126 MHz, Chloroform-*d*) δ 157.7, 134.6, 129.2, 113.8, 68.1, 55.3, 39.0, 34.4, 33.1, 31.7.

HRMS-APPI m/z calcd for $C_{14}H_{21}O_2$ ($[M+H]^+$) 221.1536, found 221.1536.

4-(2-Cyclohexylethyl)-N,N-dimethylaniline (14c)[31]

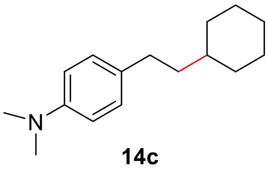

14c

Following the general procedure, the title compound was isolated as a 1:1 mixture of the title compound and 4-ethyl-N,N-dimethylaniline (150 mg, 52% yield, 52% purity).
^{1}H NMR (300 MHz, Acetonitrile-*d*$_3$) δ 7.05 (d, *J* = 8.7 Hz, 2H), 6.71 (d, *J* = 8.6 Hz, 2H), 2.89 (d, *J* = 1.0 Hz, 6H), 2.53 (td, *J* = 8.0, 4.1 Hz, 2H), 1.97 (dd, *J* = 2.9, 2.0 Hz, 1H), 1.88 – 1.58 (m, 4H), 1.46 (ddd, *J* = 9.9, 8.0, 6.3 Hz, 2H), 1.33 – 1.07 (m, 4H), 1.04 – 0.72 (m, 2H).

^{13}C NMR (75 MHz, Acetonitrile-*d*$_3$) δ 149.2, 131.1, 128.8, 113.0, 40.1, 39.6, 37.0, 33.1, 31.7, 26.5, 26.2.

GC-MS-EI analysis: m/z 231 (18%), 134 (100%), 118 (12%), 91 (10%)

4-(4-(Trifluoromethyl)phenethyl)tetrahydro-2H-pyran (16c)

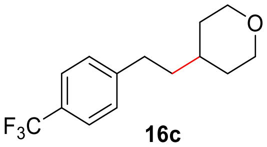

16c

Following the general procedure, the title compound was isolated as a colorless oil (108 mg, 56%).

^{1}H NMR (500 MHz, Chloroform-*d*) δ 7.51 (d, *J* = 8.0 Hz, 2H), 7.26 (d, *J* = 8.0 Hz, 2H), 4.02 – 3.88 (m, 2H), 3.35 (td, *J* = 11.8, 2.1 Hz, 2H), 2.74 – 2.59 (m, 2H), 1.67 – 1.44 (m, 5H), 1.31 (dtd, *J* = 13.2, 11.3, 5.6 Hz, 2H).
^{13}C NMR (126 MHz, Chloroform-*d*) δ 146.7, 128.6, 128.2 (q, *J* = 32 Hz), 125.4 (q, *J* = 270 Hz), 125.3 (q, *J* = 4 Hz), 123.3, 68.0, 38.4, 34.5, 33.0, 32.6.

HRMS-APPI calcd for $C_{14}H_{18}F_3O$ ($[M+H]^+$) 259.1304, found 259.1300,

1-(2-Cyclohexylethyl)naphthalene (18c)[32]

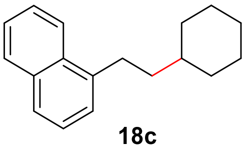

18c

Following the general procedure, the title compound was isolated as a colorless oil (121 mg, 68%).

^{1}H NMR (500 MHz, Chloroform-*d*) δ 8.06 (dd, *J* = 10.9, 5.5 Hz, 1H), 7.86 (dt, *J* = 8.1, 4.0 Hz, 1H), 7.71 (t, *J* = 6.7 Hz, 1H), 7.60 – 7.30 (m, 4H), 3.11 (ddq, *J* = 16.0, 8.2, 4.3, 3.7 Hz, 2H), 2.05 – 0.62 (m, 13H).

^{13}C NMR (126 MHz, Chloroform-*d*) δ 140.3, 139.5, 134.0, 131.9, 128.8, 126.4, 125.7, 125.4, 124.9, 123.9, 38.8, 38.0, 33.4, 30.5, 26.8, 26.5.

GC-MS-EI analysis: m/z 238 (25%), 142 (100%), 115 (30%), 97 (2.5%), 55 (32%)

1-(2-Cyclohexylethyl)-2-methylbenzene (19c)[33]

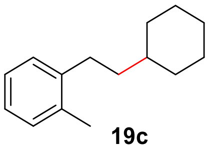

19c

Following the general procedure, the title compound was isolated as a colorless oil (79 mg, 52%).

^{1}H NMR (300 MHz, Chloroform-*d*) δ 7.10 (t, *J* = 1.9 Hz, 4H), 2.75 – 2.51 (m, 2H), 2.28 (s, 3H), 1.97 – 1.62 (m, 5H), 1.49 – 1.35 (m, 2H), 1.32 – 1.06 (m, 4H), 0.96 (td, *J* = 11.9, 3.1 Hz, 2H).
^{13}C NMR (126 MHz, Chloroform-*d*) δ 141.5, 135.8, 130.1, 128.7, 125.9, 125.7, 38.2, 37.9, 33.4, 30.7, 26.7, 26.4, 19.2.

GC-MS-EI analysis: m/z 202 (22%), 119 (5%), 106 (100%), 91 (25%), 55 (40%)

2-(2-Cyclohexylethyl)thiophene (21c)[34]

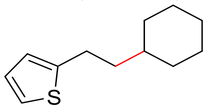

21c

Following the general procedure, the title compound was isolated as a yellow oil (102 mg, 70%).

^{1}H NMR (300 MHz, Chloroform-*d*) δ 7.08 (dd, *J* = 5.1, 1.2 Hz, 1H), 6.89 (dd, *J* = 5.1, 3.4 Hz, 1H), 6.75 (dt, *J* = 3.4, 1.0 Hz, 1H), 3.02 – 2.68 (m, 2H), 1.93 – 1.63 (m, 6H), 1.42 – 1.06 (m, 5H), 0.91 (q, *J* = 11.1, 10.6 Hz, 2H).
^{13}C NMR (126 MHz, Chloroform-*d*) δ 146.2, 126.6, 123.8, 122.7, 39.5, 37.1, 33.2, 27.3, 26.7, 26.3.
GC-MS-EI analysis: m/z 196 (21%), 149 (20%), 123 (18%), 97 (100%), 87 (38%)

1-(2-Cyclohexylethyl)-1H-pyrazole (22c)

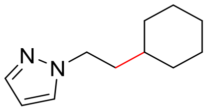

22c

Following the general procedure, the title compound was isolated as a yellow oil (83 mg, 62%).

^{1}H NMR (300 MHz, Chloroform-*d*) δ 7.47 (d, *J* = 1.9 Hz, 1H), 7.35 (d, *J* = 2.3 Hz, 1H), 6.21 (t, *J* = 2.1 Hz, 1H), 4.30 – 4.00 (m, 2H), 1.83 – 1.61 (m, 7H), 1.34 – 1.09 (m, 5H), 1.01 – 0.66 (m, 1H).
^{13}C NMR (126 MHz, Chloroform-*d*) δ 139.0, 128.6, 105.2, 49.9, 37.9, 35.1, 33.0, 26.5, 26.1.

HRMS-APCI m/z calcd for $C_{11}H_{19}N_2$ ($[M+H]^+$) 179.1543, found 179.1543.

Octylcyclohexane[35]

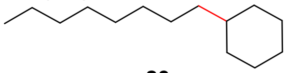

23c

Following the general procedure, the title compound was isolated as a colorless oil (75 mg, 51%).

^{1}H NMR (300 MHz, Chloroform-*d*) δ 1.87 – 1.58 (m, 5H), 1.28 (m, 18H), 0.96 – 0.82 (m, 5H).

^{13}C NMR (126 MHz, Chloroform-*d*) δ 37.7, 37.6, 33.5, 31.9, 30.2, 30.0, 29.7, 29.4, 26.9, 26.8, 26.5, 22.7, 14.1.

GC-MS-EI analysis: m/z 196 (1%), 97 (8%), 83 (100%), 67 (20%), 55 (60%)

Butylbenzene (26c)[36]

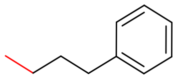

26c

Following the general procedure, the title compound was isolated as a colorless oil (57 mg, 53%).

^{1}H NMR (500 MHz, Chloroform-*d*) δ 7.29 – 7.21 (m, 2H), 7.19 – 7.10 (m, 3H), 2.66 – 2.48 (m, 2H), 1.69 – 1.55 (m, 2H), 1.37 – 1.26 (m, 4H), 0.87 (t, *J* = 6.9 Hz, 3H).
^{13}C NMR (126 MHz, Chloroform-*d*) δ 142.9, 128.4, 128.2, 125.6, 35.9, 31.6, 22.6, 14.0.

GC-MS-EI analysis: m/z 134 (25%), 105 (8%), 91 (100%), 65 (15%), 52 (7%)

Pentylbenzene (27c)[37]

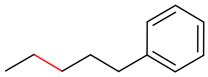

27c

Following the general procedure, the title compound was isolated as a colorless oil (62 mg, 56%).

^{1}H NMR (500 MHz, Chloroform-*d*) δ 7.37 – 7.27 (m, 2H), 7.24 – 7.17 (m, 3H), 4.31 – 3.80 (m, 2H), 3.40 (td, *J* = 11.8, 2.1 Hz, 2H), 2.86 – 2.47 (m, 2H), 1.89 – 1.50 (m, 6H), 1.48 – 1.16 (m, 3H).
^{13}C NMR (126 MHz, Chloroform-*d*) δ 142.6, 125.7, 68.1, 38.7, 34.5, 33.1, 32.7. 22.6, 14.0.

GC-MS-EI analysis: m/z 148 (20%), 105 (10%), 91 (100%), 65 (12%)

tert-Butyl 4-methylpiperidine-1-carboxylate (28c)[38]

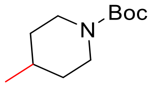

28c

Following the general procedure, the title compound was isolated as a colorless oil (66 mg, 44%).

^{1}H NMR (500 MHz, Chloroform-*d*) δ 3.33 (t, *J* = 5.5 Hz, 4H), 1.62 – 1.45 (m, 5H), 1.43 (d, *J* = 1.0 Hz, 9H), 0.91 (d, *J* = 6.5 Hz, 3H).
^{13}C NMR (126 MHz, Chloroform-*d*) δ 154.9, 79.1, 37.7, 29.2, 28.5, 24.5, 11.2.

GC-MS-EI analysis: m/z 199 (1.5%), 143 (15%), 126 (10%), 98 (30%), 84 (9%)

tert-Butyl 4-ethylpiperidine-1-carboxylate (29c)[39]

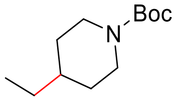

29c

Following the general procedure, the title compound was isolated as a yellow oil (89 mg, 56%).

^{1}H NMR (500 MHz, Chloroform-*d*) δ 3.42 – 3.29 (m, 4H), 1.83 – 1.44 (m, 5H), 1.42 (d, *J* = 1.2 Hz, 9H), 1.02 (qd, *J* = 12.6, 4.5 Hz, 2H), 0.86 (t, *J* = 7.2 Hz, 3H).
^{13}C NMR (126 MHz, Chloroform-*d*) δ 154.9, 79.1, 41.1, 31.0, 28.5, 26.9, 24.5, 21.9.

GC-MS-EI analysis: m/z 213 (2.5%), 157 (17.5%), 108 (10%), 112 (20%), 57 (100%)

2-(3-Cyclohexylpropyl)isoindoline-1,3-dione (31c)[40]

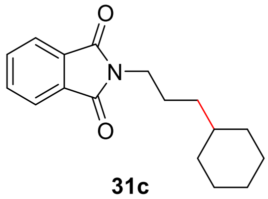

31c

Following the general procedure, the title compound was isolated as a yellow oil (120 mg, 59%).

^{1}H NMR (300 MHz, Chloroform-*d*) δ 7.97 – 7.81 (m, 2H), 7.77 – 7.68 (m, 2H), 3.68 (t, J = 7.3 Hz, 2H), 1.91 – 1.52 (m, 8H), 1.39 – 1.03 (m, 4H), 1.03 – 0.73 (m, 3H).
^{13}C NMR (126 MHz, Chloroform-*d*) δ 168.5, 133.8, 132.2, 123.2, 39.6, 38.4, 37.3, 34.5, 33.3, 26.6, 26.3, 26.0.
GC-MS-EI analysis: m/z 271 (80%), 175 (25%), 160 (100%), 133 (40%), 105 (30%)

Supplementary References

1. Komeyama, K.; Michiyuki, T.; Osaka, I. Nickel/Cobalt-Catalyzed C(sp3)-C(sp3) Cross-Coupling of Alkyl Halides with Alkyl Tosylates. *ACS Catalysis* **2019**, *9*, 9285-9291.

2. Comagic, S.; Schirrmacher, R. Efficient and Mild Ytterbium(III)-Catalyzed Tosylation of Alcohols. *Synthesis*, **2004**, *6*, 885-888.

3. Feng, S.; Li, J.; Wei, J. Ionic liquid brush as an efficient and reusable heterogeneous catalytic assembly for the tosylation of phenols and alcohols in neat water. *New Journal of Chemistry*, **2017**, *41*, 4743-4746.

4. Blaess, M.; Bibak, N.; Claus, R. A.; Kohl, M.; Bonaterra, G. A.; Kinscherf, R.; Laufer, S.; Deigner, H. P. NB 06: From a simple lysosomotropic aSMase inhibitor to tools for elucidating the role of lysosomes in signaling apoptosis and LPS-induced inflammation. *European Journal of Medicinal Chemistry* **2018**, *153*, 73-104.

5. Byun, Y. S.; Lightner, D. A. Long-range exciton coupling from dipyrrinone chromophores. *Tetrahedron*, **1991**, *47*, 9759-9772.

6. Ilies, L.; Matsubara, T.; Ichikawa, S.; Asako, S.; Nakamura, E. Iron-Catalyzed Directed Alkylation of Aromatic and Olefinic Carboxamides with Primary and Secondary Alkyl Tosylates, Mesylates, and Halides. *J. Am. Chem. Soc.* **2014**, *136*, 13126-13129.

7. Son, W. S.; Jeong, K. S.; Lim, S. M.; Pae, A. N. Structural hybridization of pyrrolidine-based T-type calcium channel inhibitors and exploration of their analgesic effects in a neuropathic pain model. *Bioorg. Med. Chem. Lett.* **2019**, *29*, 1168-1172.

8. Bhupathiraju, N. V. S. D. K.; Younes, A.; Cao, M.; Ali, J.; Cicek, H. T.; Tully, K. M.; Ponnala, S.; Babich, J. W.; Deri, M. A.; Lewis, J. S.; Francesconi, L. C.; Drain, C. M. Improved synthesis of the bifunctional chelator p-SCN-Bn-HOPO. *Org. Biom. Chem.* **2019**, *17*, 6866-6871.

9. Gardner, R. A.; Delcros, J.-G.; Konate, F.; Breitbeil, F.; Martin, B.; Sigman, M.; Huang, H.; Phanstiel IV, O. N-Substituent Effects in the Selective Delivery of Polyamine Conjugates into Cells Containing Active Polyamine Transporters. *J. Med. Chem.* **2004**, *47*, 6055-6069.

10. Cheng, K.; Kim, I. J.; Lee, M. J.; Adah, S. A.; Raymond, T. J.; Bilsky, E. J.; Aceto, M. D.; May, E. L.; Harris, L. S.; Coop, A.; Dersch, C. M.; Rothman, R. B.; Jacobson, A. E.; Rice, K. C. Opioid

ligands with mixed properties from substituted enantiomeric N-phenethyl-5-phenylmorphans. Synthesis of a μ-agonist δ-antagonist and δ-inverse agonists. *Org. Biomol. Chem.* **2007**, *5*, 1177-1190.

11. Lathioor, E. C.; Leigh, W. J.; St. Pierre, M. J. Geometrical Effects on Intramolecular Quenching of Aromatic Ketone (π,π*) Triplets by Remote Phenolic Hydrogen Abstraction. *J. Am. Chem. Soc.* **1999**, *121*, 11984-11992.

12. Tang, D. T. D.; Collins, K. D.; Ernst, J. B.; Glorius, F. Pd/C as a Catalyst for Completely Regioselective C-H Functionalization of Thiophenes under Mild Conditions. *Angew. Chem. Int. Ed.* **2014**, *53*, 1809-1813.

13. Inoue, Y.; Terasawa, T.; Takasugi, H.; Nagayoshi, A.; Ueshima, K.; Sawada, M; Furukawa, Y.; Mikami, M.; Hinoue, K.; Fukumoto, D. Amide compounds for the treatment of hyperlipidemia, WO2004039795A2, May 13, 2004.

14. Chu, Y.-H.; Hwang, C.-C.; Chen, C.-Y.; Tseng, M.-J. Combinatorial discovery of thermoresponsive cycloammonium ionic liquids. *Chem. Commun.* **2020**, *56*, 11855-11858.

15. Giardello, M. A.; Conticello, V. P. Brard, L.; Gagne, M. R.; Marks, T. J. Chiral Organolanthanides Designed for Asymmetric Catalysis. A Kinetic and Mechanistic Study of Enantioselective Olefin Hydroamination/Cyclization and Hydrogenation by C1-Symmetric Me2Si(Me4C5)(C5H3R*)Ln Complexes where R* = Chiral Auxiliary. *J. Am. Chem. Soc.* **1994**, *116*, 10241-10254.

16. Pasupuleti, B. G.; Khongsti, K.; Das, B.; Bez, G. 1,2,3-Triazole tethered 1,2,4-trioxanes: Studies on their synthesis and effect on osteopontin expression in MDA-MB-435 breast cancer cells. *Eur. J. Med. Chem.* **2020**, *186*, 111908.

17. Buysse, A. M.; Niyaz, N. M.; Demeter, D. A.; Zhang, Y.; Walsh, M. J.; Kubota, A.; Hunter, R.; Trullinger, T. K.; Lowe, C. T.; Knueppel, D.; Patny, A.; Garizi, N.; LePlae, Jr., P. R.; Wessels, F.; Ross, Jr., R.; Deamicis, C.; Borromeo, P. Pesticidal compositions and processes related thereto US20130288893A1, Oct 31, 2013.

18. Cai, Z. R; Jabri, S. Y; Jin, H.; Lansdown, R. A.; Metobo, S. E.; Mish, M. R.; Pastor, R. M. Integrase inhibitors, US8008287, Aug 30, 2011.

19. Martinkus, K. J.; Tann, C. H.; Gould, S. J. The biosynthesis of the streptolidine moiety in streptothricin F. *Tetrahedron* 1983, *39*, 3493-3505.

20. Bae, J.; Mook Kang, K.; Kim, Y. C. Discovery of 5-methyl-1H-benzo[d]imidazole derivatives as novel P2X3 Receptor antagonists. *Bioorg. Med. Chem. Lett.* 2022, *72*, 128820.

21. Huang, M.; Hu, J.; Shi, S.; Friedrich, A.; Krebs, J.; Westcott, S. A.; Radius, U.; Marder, T. B. Selective, Transition Metal-free 1,2-Diboration of Alkyl Halides, Tosylates, and Alcohols. *Chem. Eur. J.* **2022**, *28*, e202200480.

22. Bam, R.; Pollatos, A. S.; Moser, A. J.; West, J. G. Mild olefin formation via bio-inspired vitamin B12 photocatalysis. *Chem. Sci.* **2021**, *12*, 1736-1744.

23. Dragovich, P. S.; Blazel, J. K.; Dao, K.; Ellis, D. A.; Li, L. S.; Murphy, D. E.; Ruebsam, F.; Tran, C. V.; Zhou, Y. Regiospecific Synthesis of Novel 6-Amino-5-hydroxypyridazin-3(2H)-ones. *Synthesis*, **2008**, *4*, 610-616.

24. Aragón, J.; Sun, S.; Pascual, D.; Jaworski, S.; Lloret-Fillol, J. Photoredox Activation of Inert Alkyl Chlorides for the Reductive Cross-Coupling with Aromatic Alkenes. *Angew. Chem. Int. Ed.* **2022**, *61*, e202114365.

25. Yus, M.; Ortiz, R. Tandem Intramolecular Carbolithiation-Lithium/Zinc Transmetallation and Applications to Carbon−Carbon Bond-Forming Reactions. *Eur. J. Org. Chem.* **2004**, 3833-3841.

26. Rahaim, Jr., R. J.; Maleczka, Jr., R. E. C−O Hydrogenolysis Catalyzed by Pd-PMHS Nanoparticles in the Company of Chloroarenes. *Org. Lett.* **2011**, *13*, 584-587.

27. Pandiri, H.; Gonnade, R. G.; Punji, B. Synthesis of quinolinyl-based pincer copper(ii) complexes: an efficient catalyst system for Kumada coupling of alkyl chlorides and bromides with alkyl Grignard reagents. *Dalton Trans.* **2018**, *47*, 16747-16754.

28. Back, T. G.; Baron, D. L.; Yang, K. Desulfurization with nickel and cobalt boride: scope, selectivity, stereochemistry, and deuterium-labeling studies. *J. Org. Chem.* **1993**, *58*, 2407-2413.

29. Figula, B. C.; Kane, D. L.; Balaraman, K.; Wolf, C. Organocuprate Cross–Coupling Reactions with Alkyl Fluorides. *Org. Lett.* **2022**, *24*, 8719-8723.

30. Petruncio, G.; Elahi-Mohassel, S.; Girgis, M.; Paige, M. Copper-catalyzed sp3-sp3 cross-coupling of turbo Grignards with benzyl halides. *Tetrahedron Lett.* **2021**, *86*, 153516.

31. Yang, Z.; Zhu, X.; Yang, S.; Cheng, W.; Zhang, X.; Zang, Z. Iridium-Catalysed Reductive Deoxygenation of Ketones with Formic Acid as Traceless Hydride Donor. *Adv. Synth. Catal.* **2020**, *362*, 5496-5505.

32. Luo, S.; Yu, D. G.; Zhu, R. Y.; Wang, X.; Wang, L.; Shi, Z. J. Fe-promoted cross coupling of homobenzylic methyl ethers with Grignard reagents via sp3 C–O bond cleavage. *Chem. Commun.* **2013**, *49*, 7794-7796.

33. Luo, S.; Yu, D. G.; Zhu, R. Y.; Wang, X.; Wang, L.; Shi, Z. J. Fe-promoted cross coupling of homobenzylic methyl ethers with Grignard reagents via sp3 C–O bond cleavage. *Chem. Commun.* **2013**, *49*, 7794-7796.

34. Kim, S. O.; An, T. K.; Chen, J.; Kang, I.; Kang, S. H.; Chung, D. S.; Park, C. E.; Kim, Y. H.; Kwon, S. K. H-Aggregation Strategy in the Design of Molecular Semiconductors for Highly Reliable Organic Thin Film Transistors. *Adv. Funct. Mater.* **2011**, *21*, 1616-1623.

35.Miyamura, H.; Suzuki, A.; Yasukawa, T.; Shū Kobayashi. Polysilane-Immobilized Rh–Pt Bimetallic Nanoparticles as Powerful Arene Hydrogenation Catalysts: Synthesis, Reactions under Batch and Flow Conditions and Reaction Mechanism. *J. Am. Chem. Soc.* **2018**, *140*, 11325-11334.

36. Kong, D.; Qiqige, Q.; McNutt, W.; Paciello, R.; Schäfer, A.; Schelwies, M.; Lundgren, R. J. Site-Selective Hydrogenation of Electron-Poor Alkenes and Dienes Enabled by a Rhodium-Catalyzed Hydride Addition/Protonolysis Mechanism. *Angew. Chem. Int. Ed.* **2022**, *61*, e202210601.

37. Okura, K.; Shirakawa, E. Single-Electron-Transfer-Induced Coupling of Alkylzinc Reagents with Aryl Iodides. *Eur. J. Org. Chem.* **2016**, 3043-3046.

38. Qin, T.; Malins, L. R.; Edwards, J. T.; Merchant, R. R.; Novak, A. J. E.; Zhong, J. Z.; Mills, R. B.; Yan, M.; Yuan, C.; Eastgate, M. D.; Baran, P. S. Nickel-Catalyzed Barton Decarboxylation and Giese Reactions: A Practical Take on Classic Transforms. *Angew. Chem. Int. Ed.* **2017**, *56*, 260-265.

39. McCallum, T.; Slavko, E.; Morin, M.; Barriault, L. Light-Mediated Deoxygenation of Alcohols with a Dimeric Gold Catalyst. *Eur. J. Org. Chem.* **2015**, 81-85.

40 Hong, S.; Song, C. E.; Ryu, D. H.; Lee, S. K.; Shin, W. S.; Lim, E. Cyclohexyl-substituted non-fullerene small-molecule acceptors for organic solar cells. *New J. Chem.* **2021**, *45*, 10373-10382.

Copies of NMR Spectra

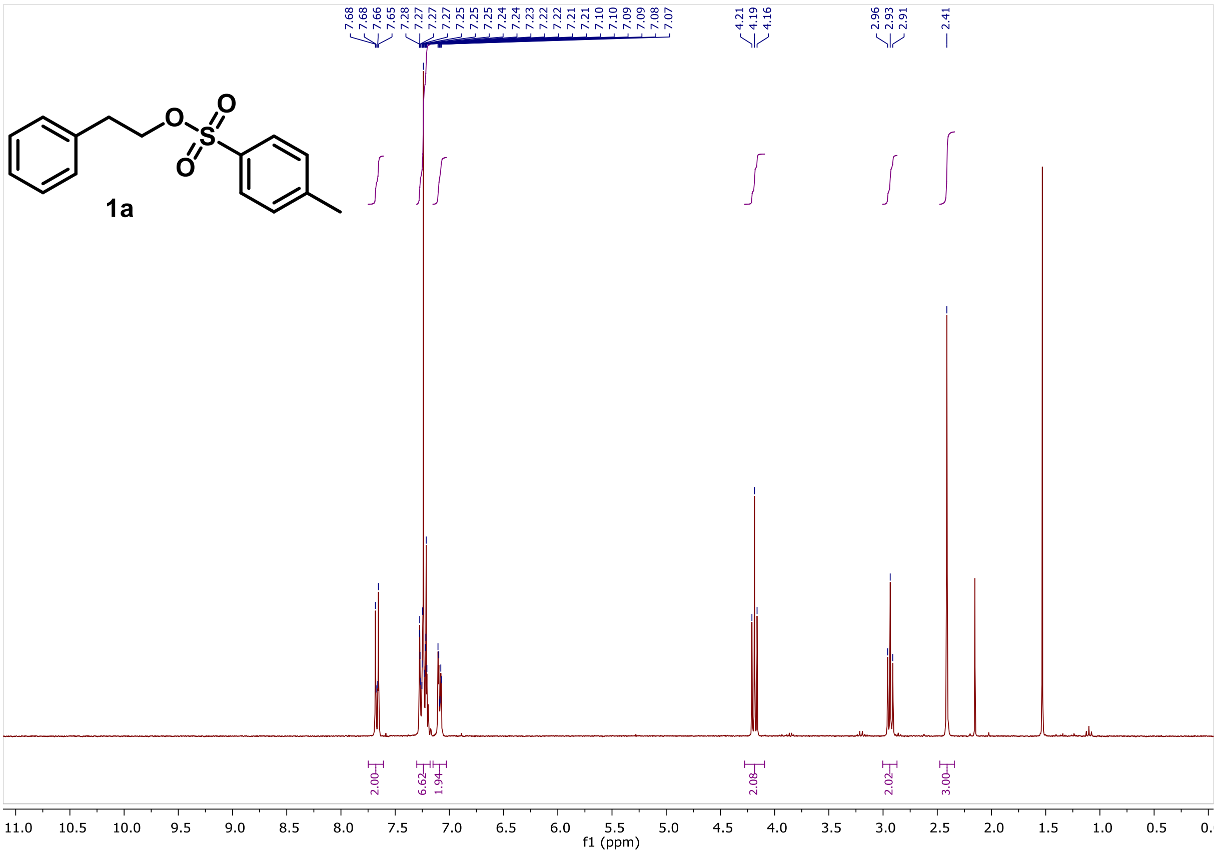

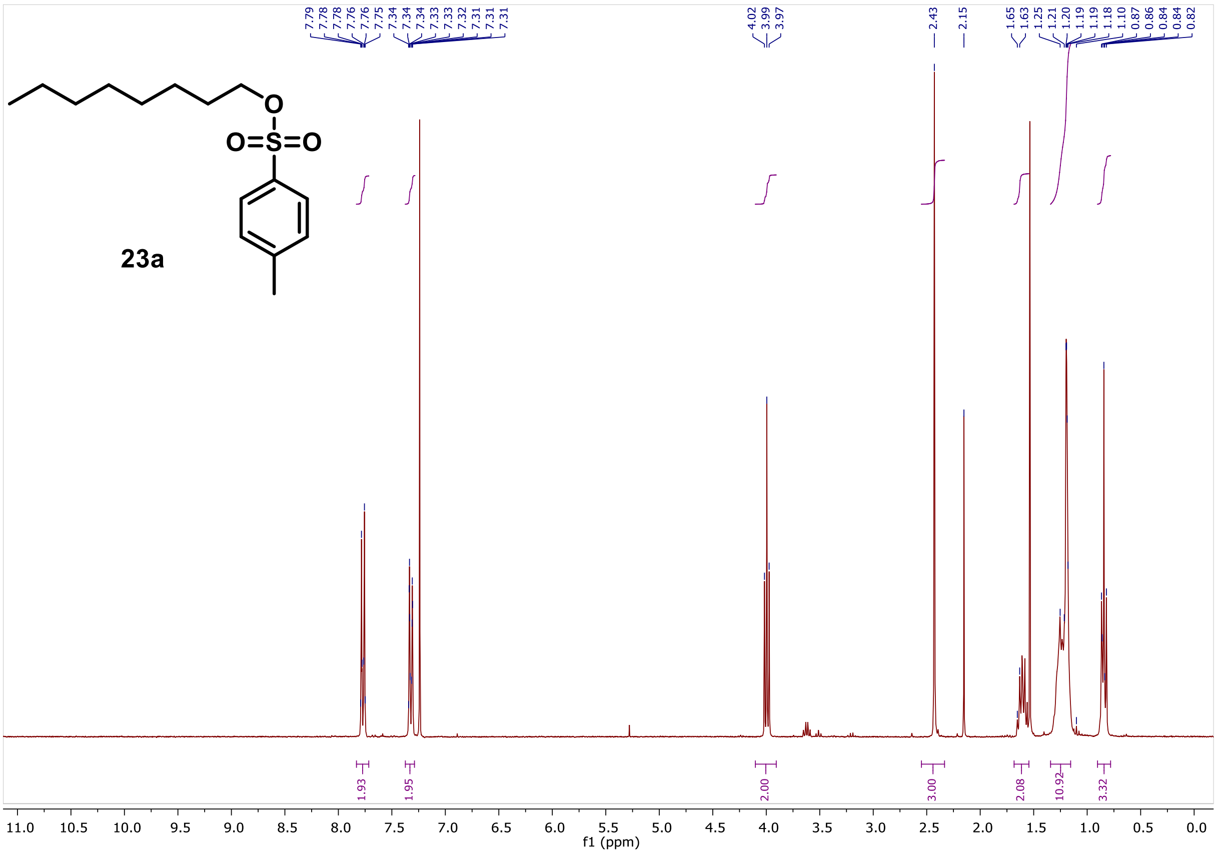

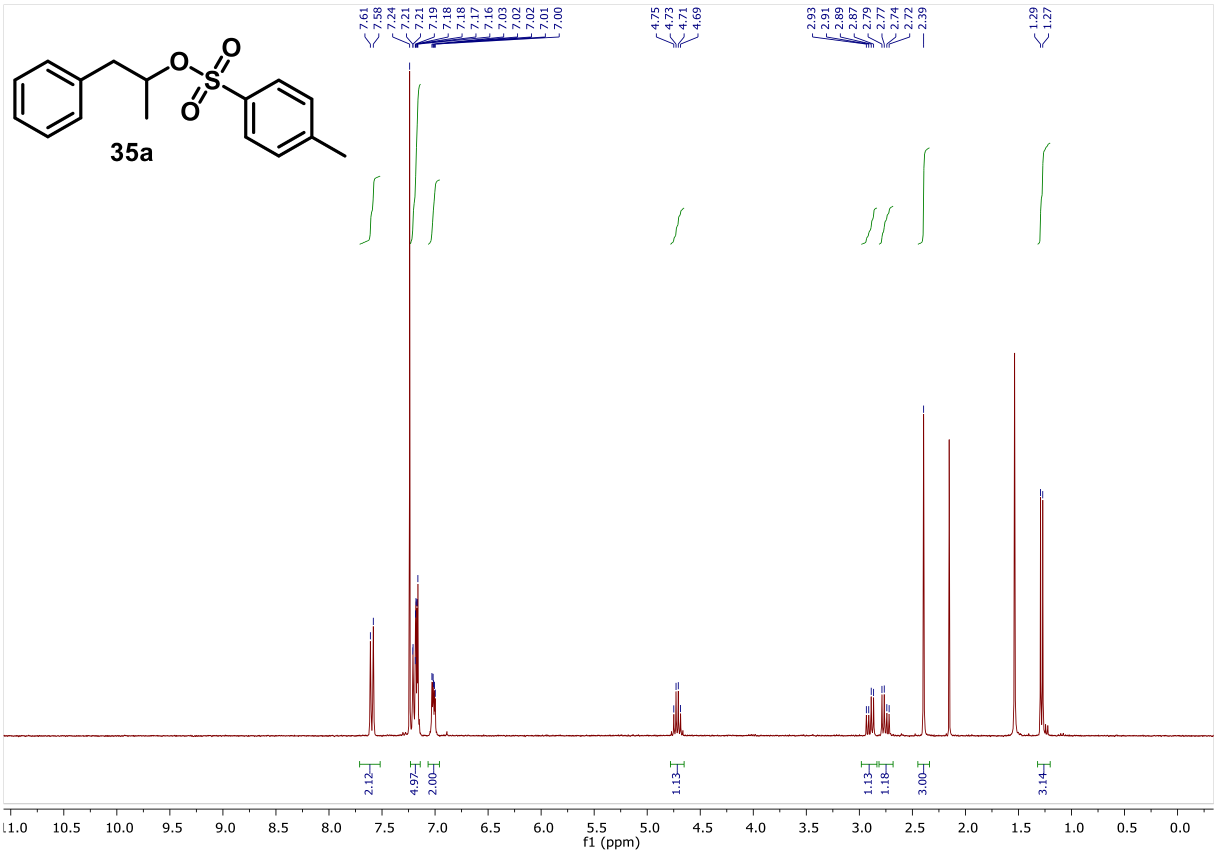
35a
f1 (ppm)

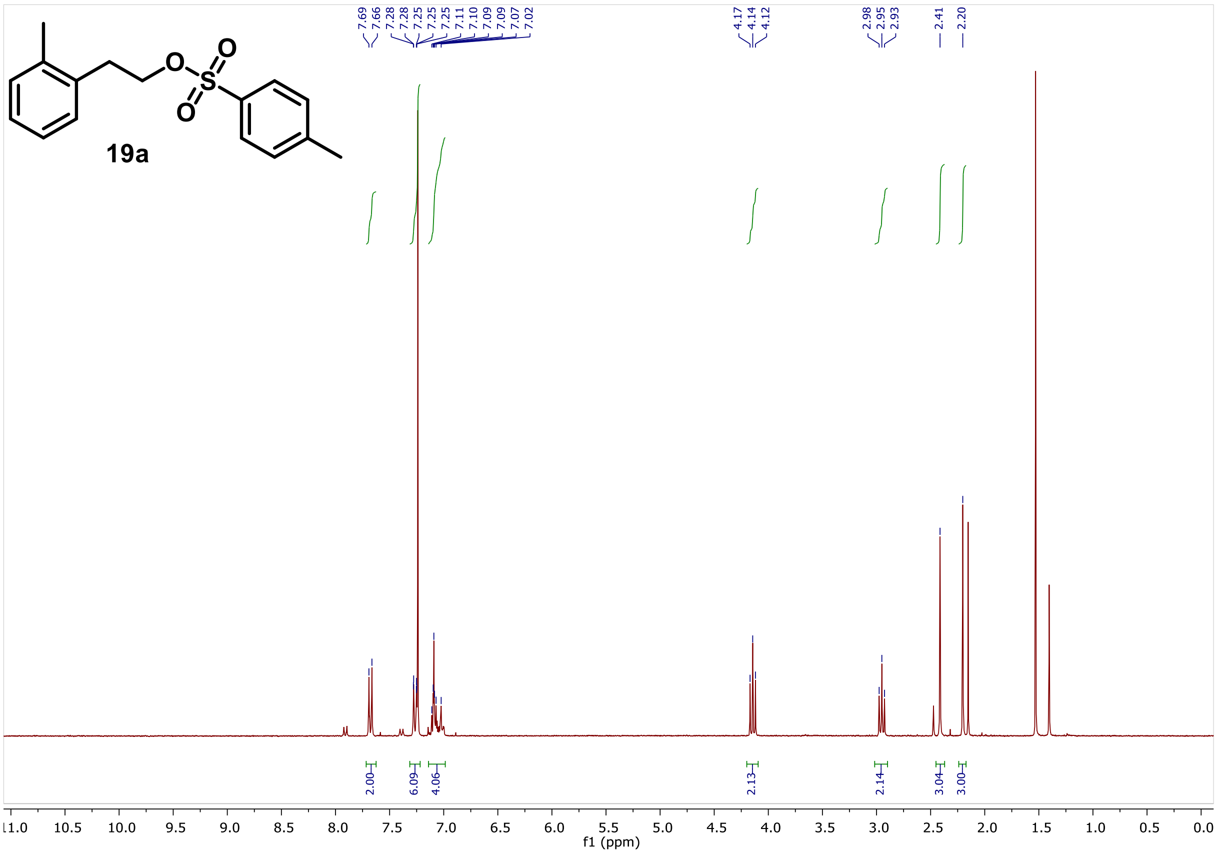
19a
f1 (ppm)

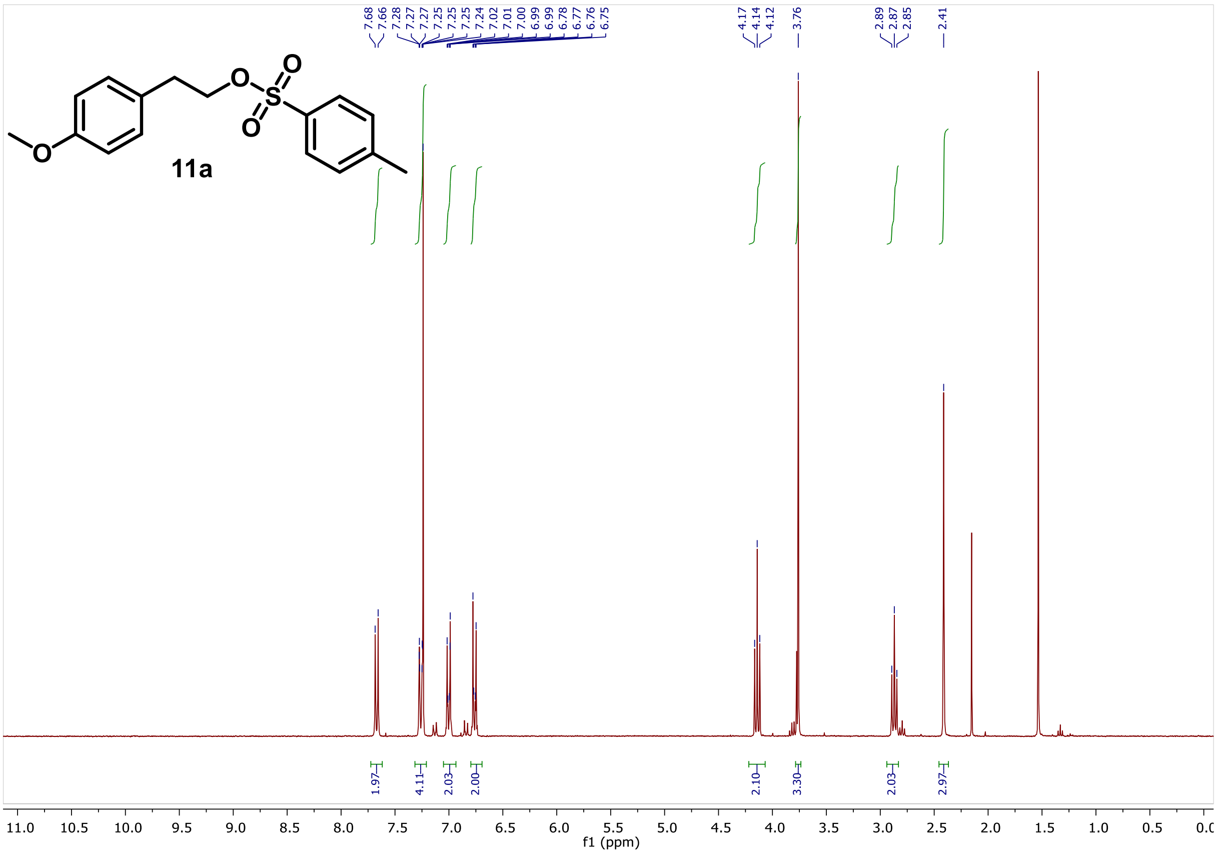
11a
f1 (ppm)

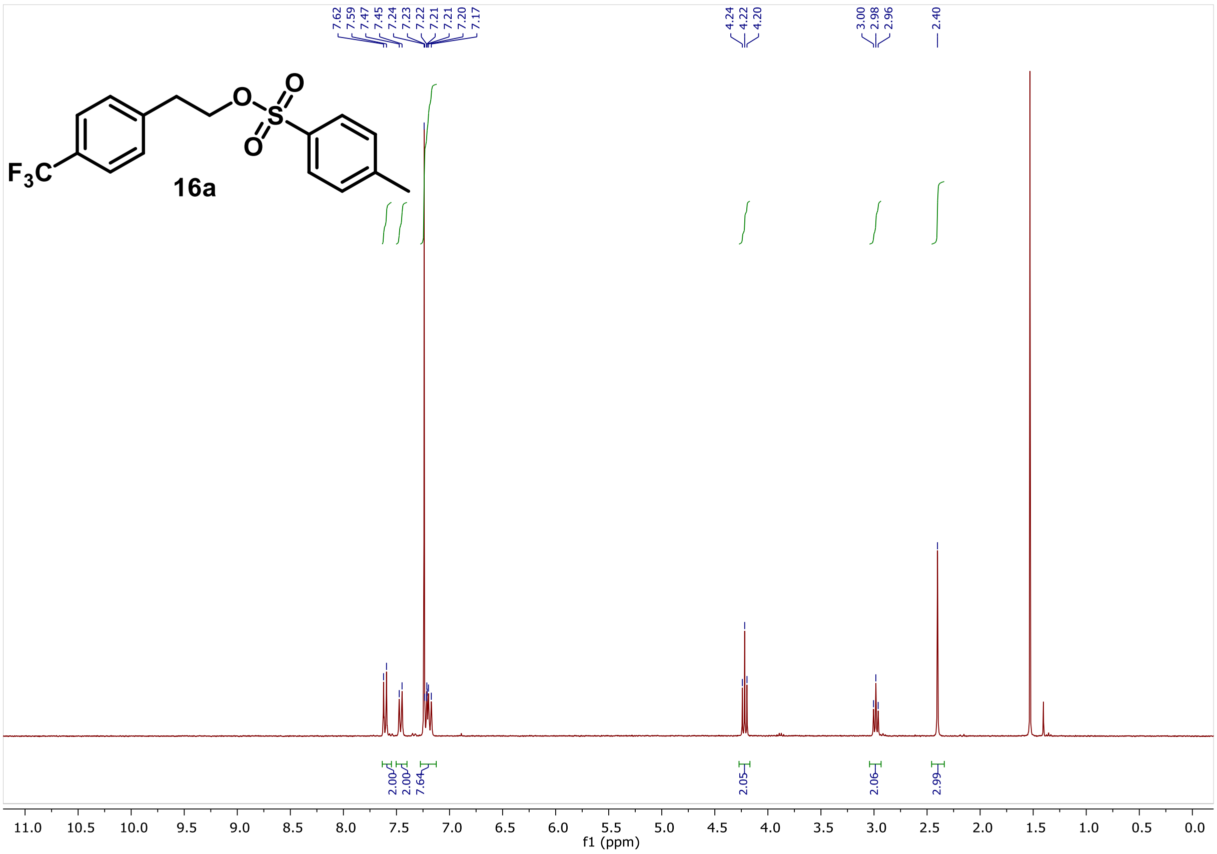
F_3C
16a
f1 (ppm)

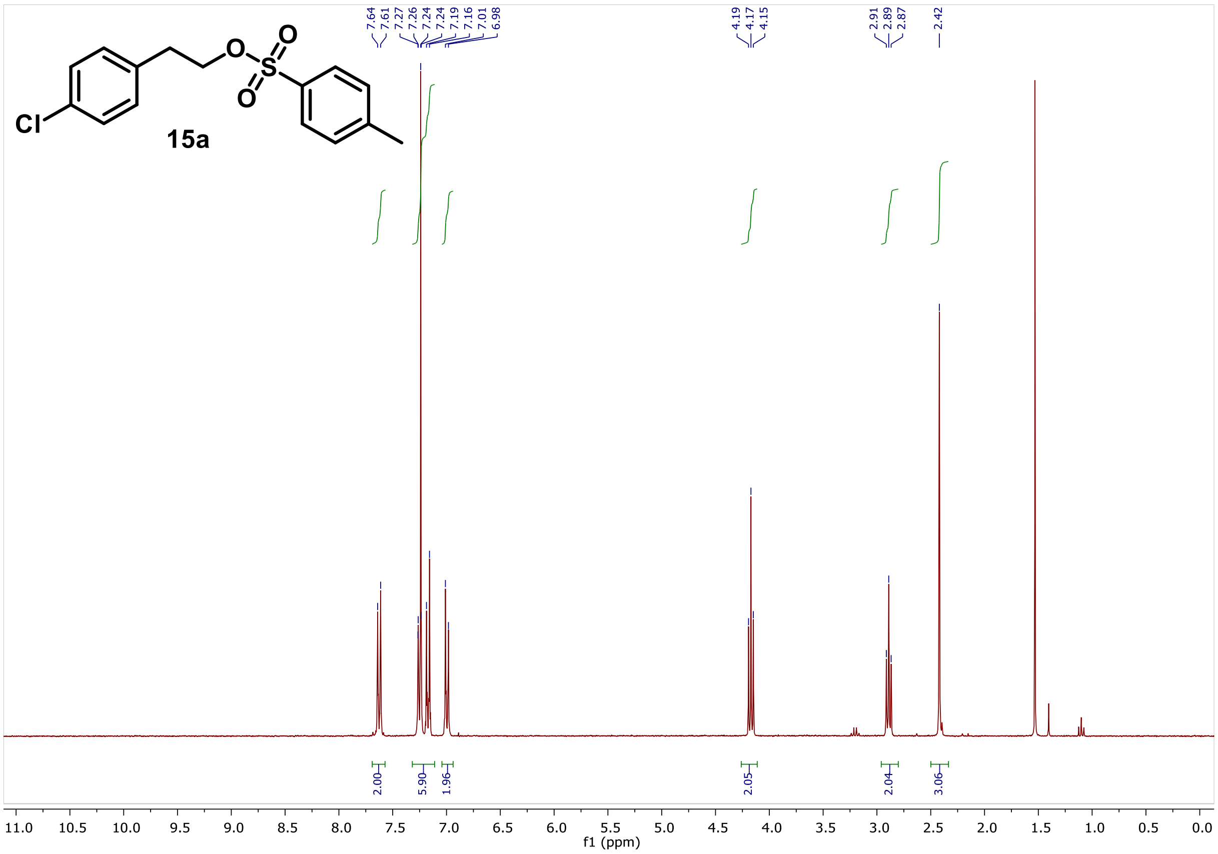
Cl
15a
f1 (ppm)

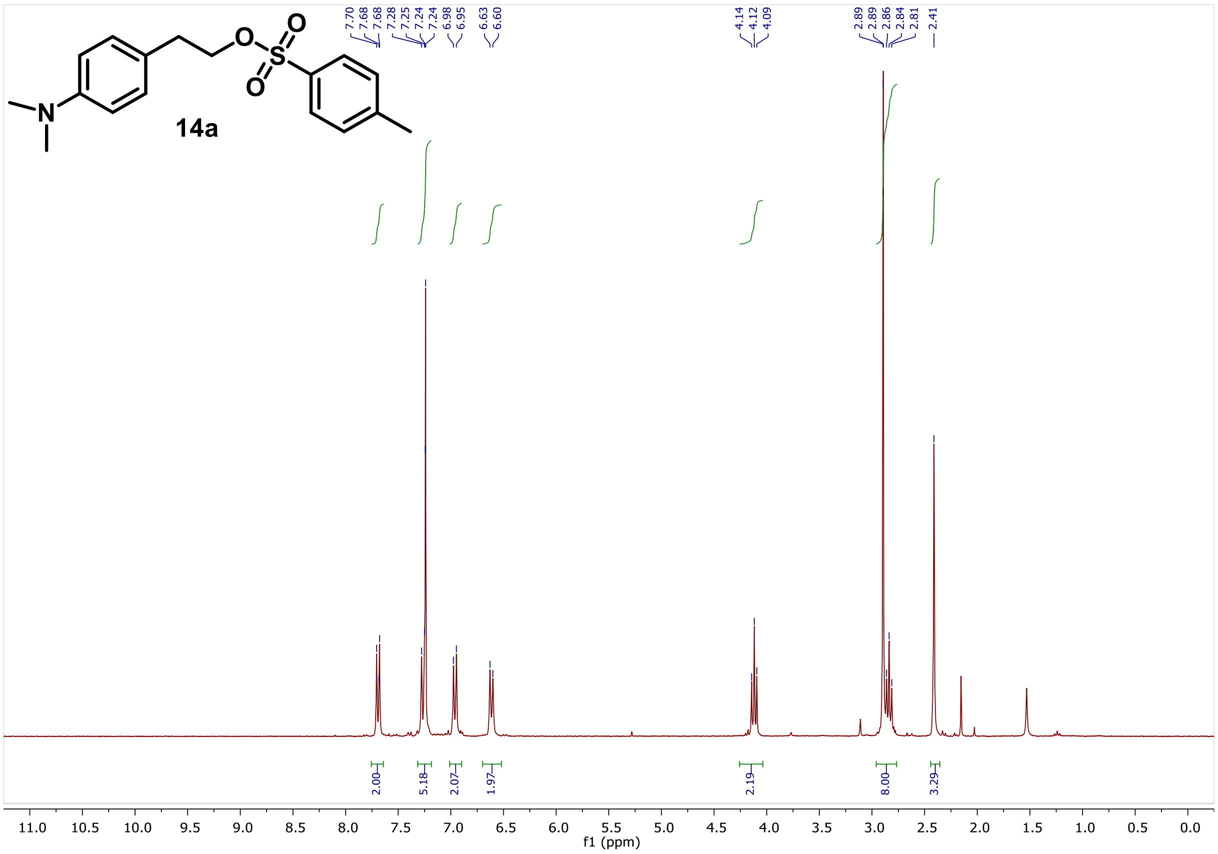
N
14a
f1 (ppm)

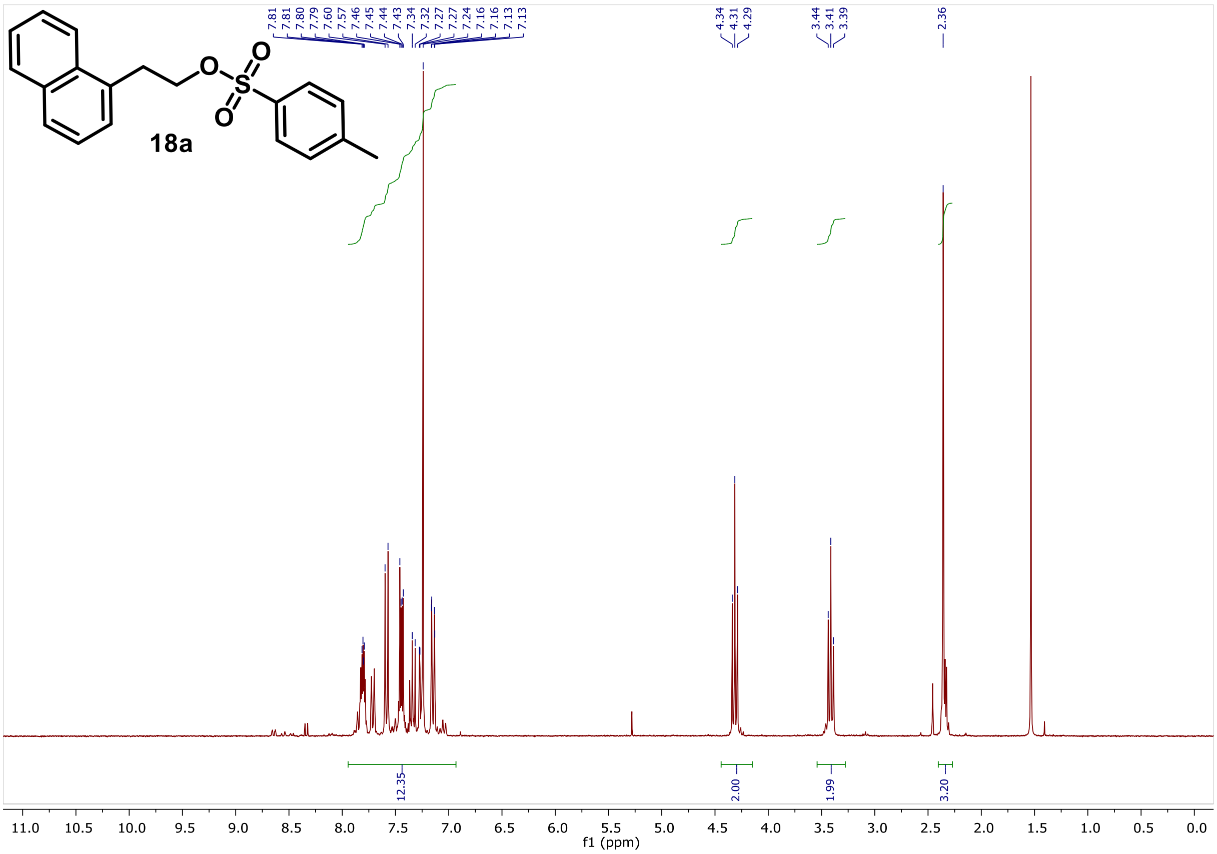

18a

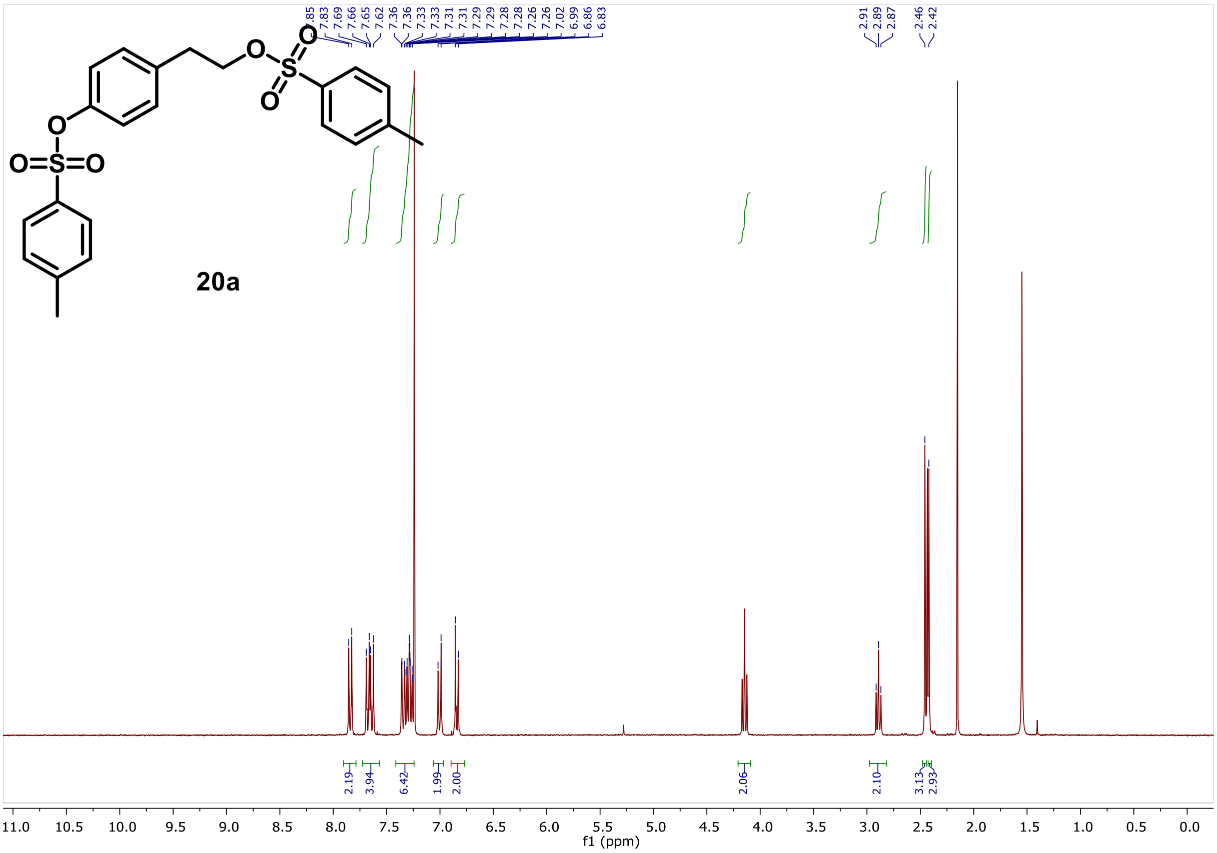

20a

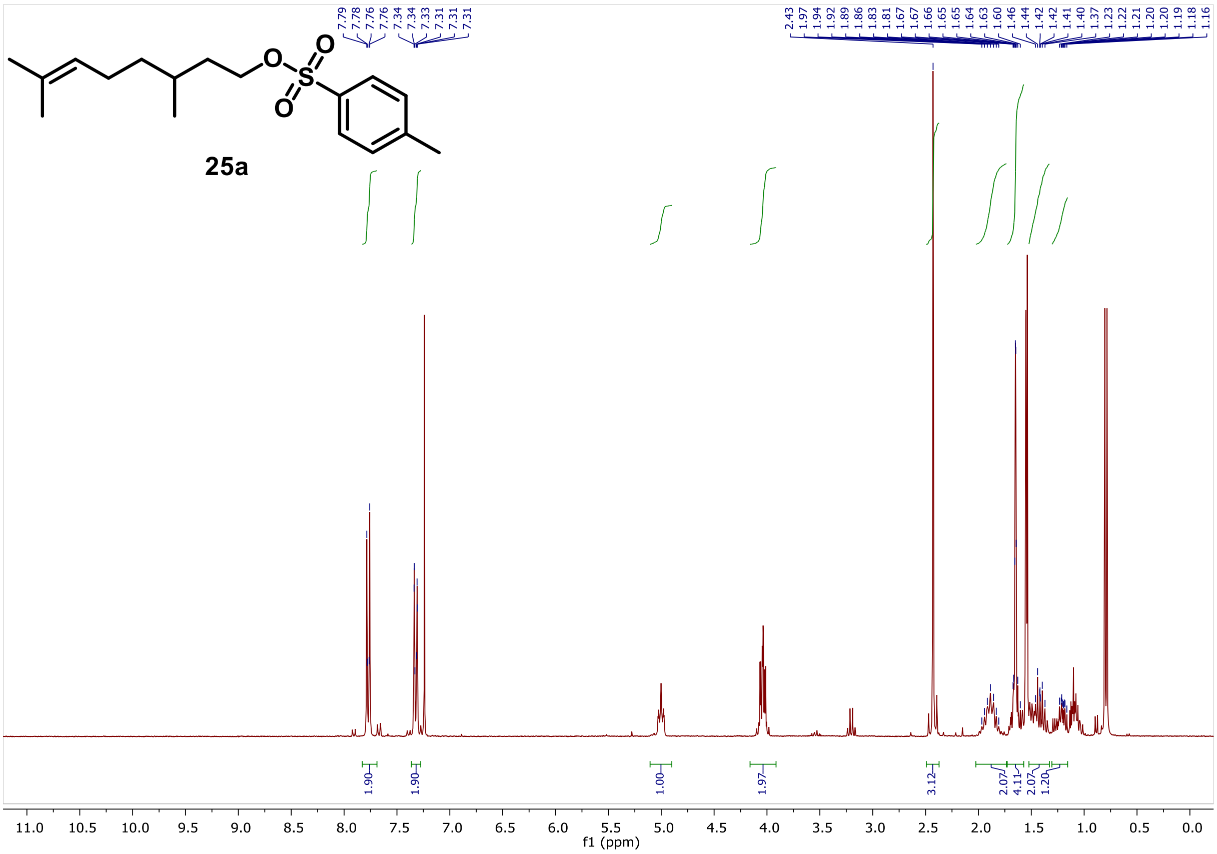
25a
f1 (ppm)

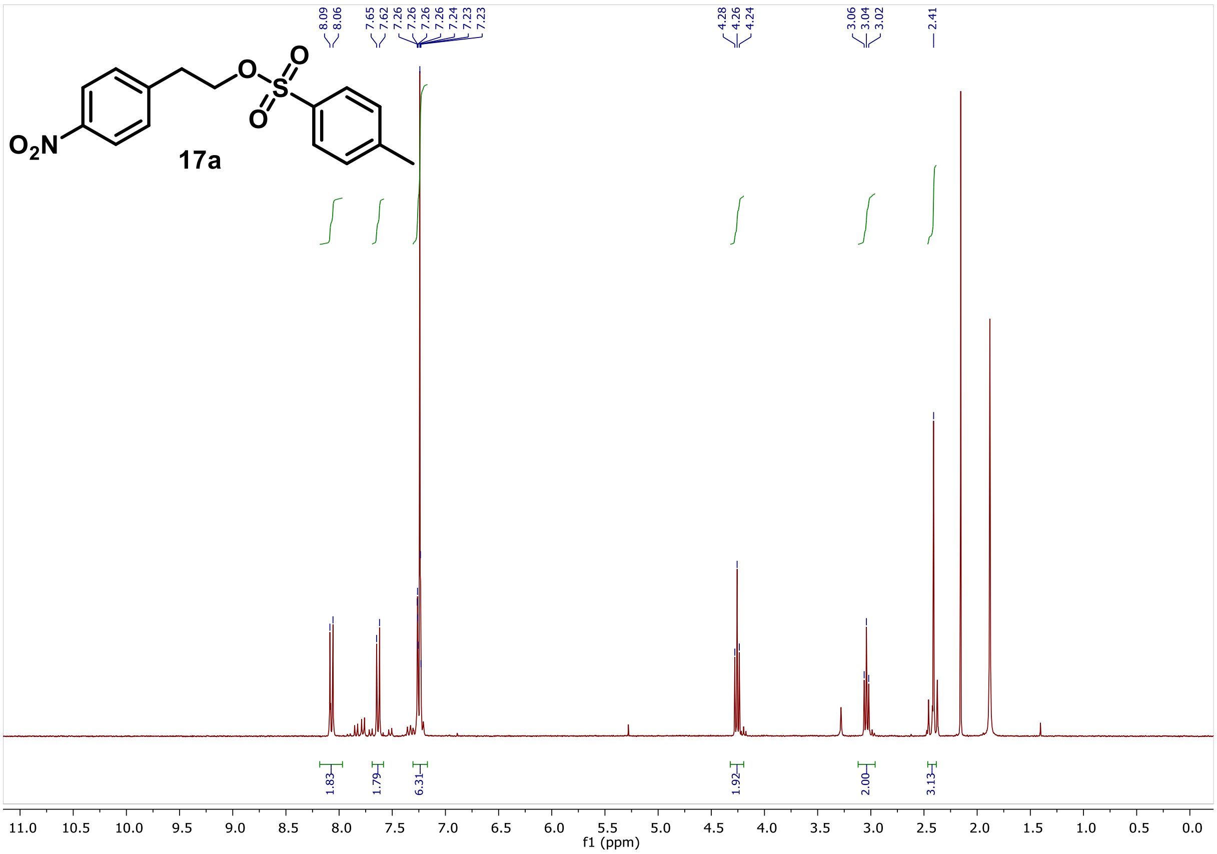
O_2N
17a
f1 (ppm)

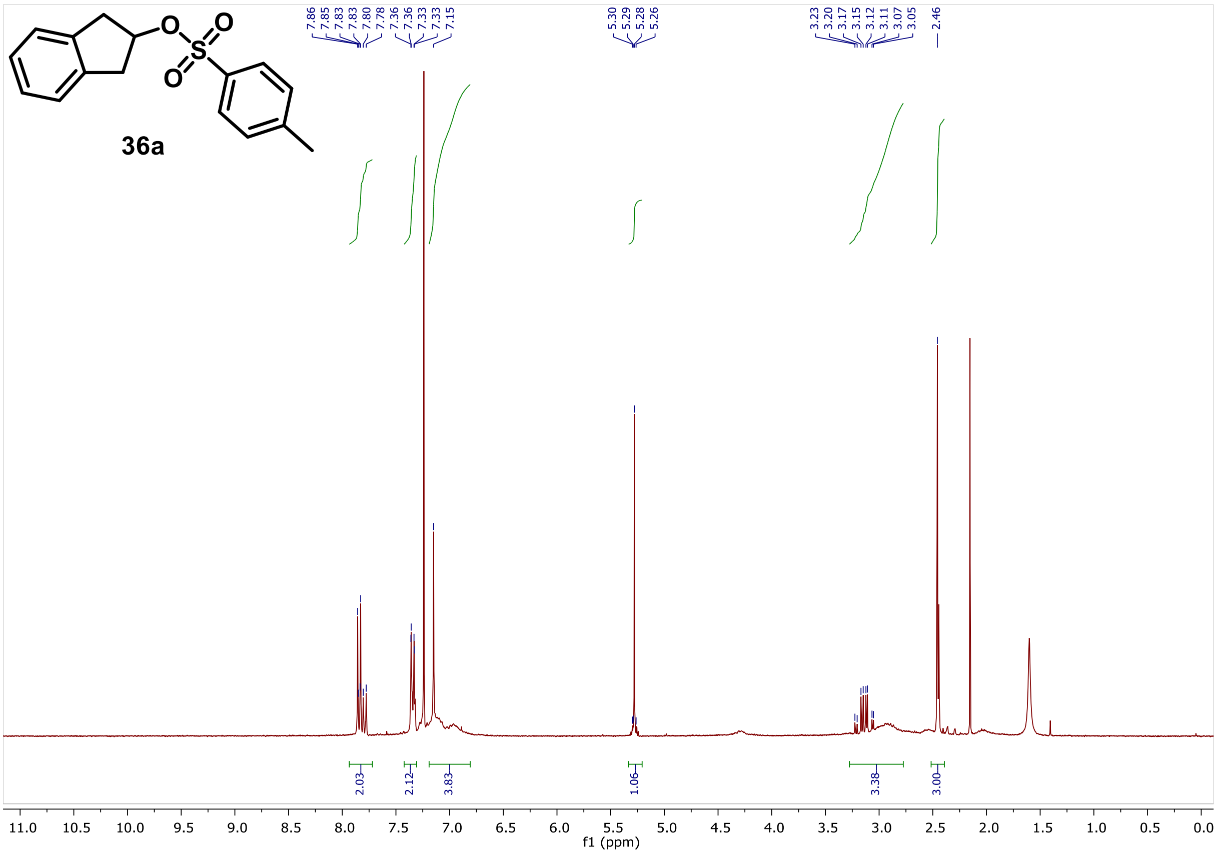

36a

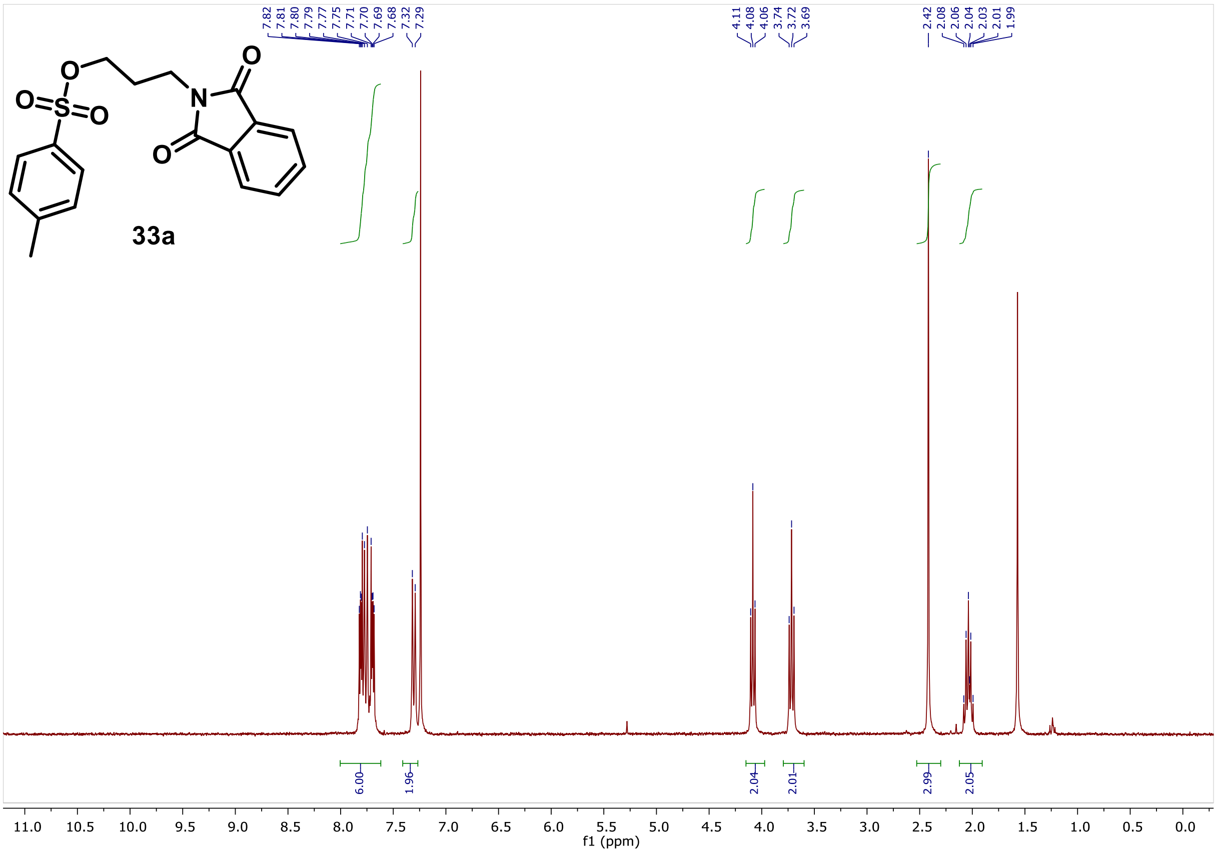

33a

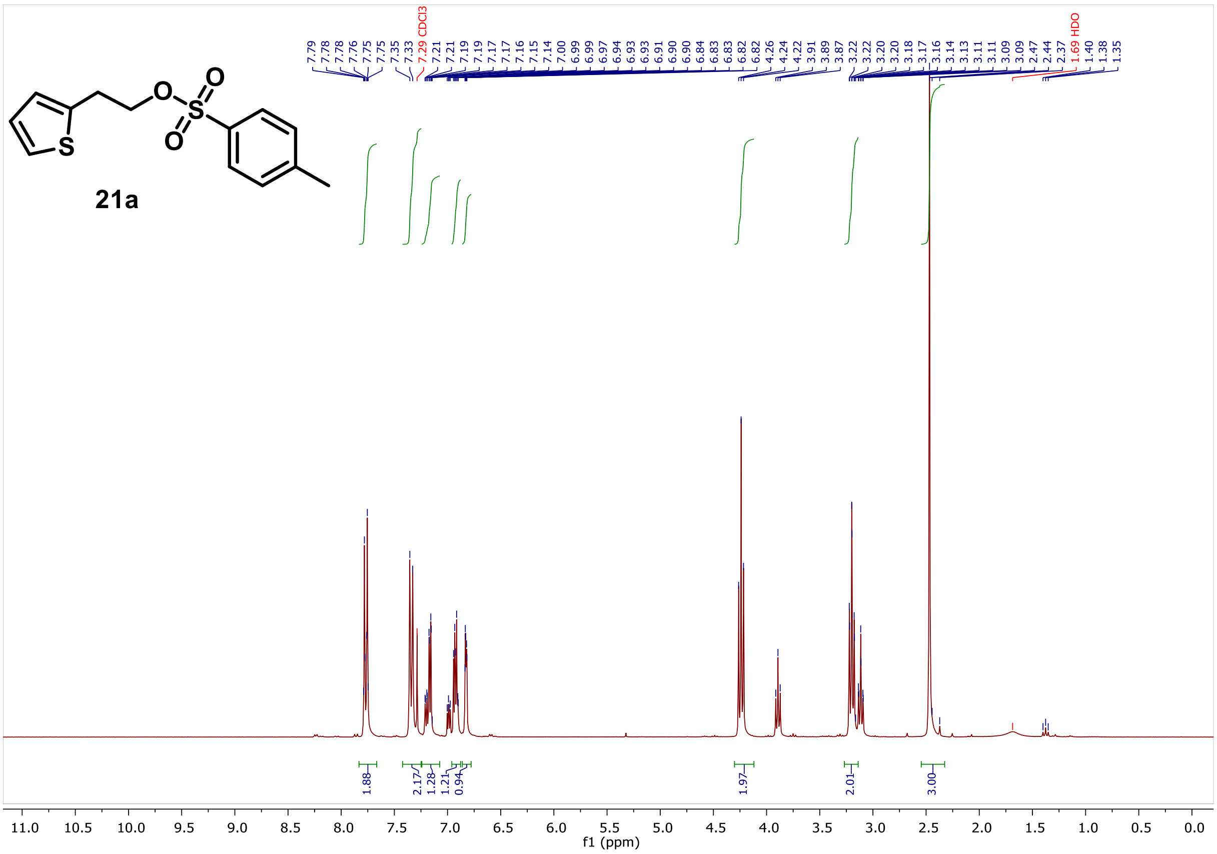

21a

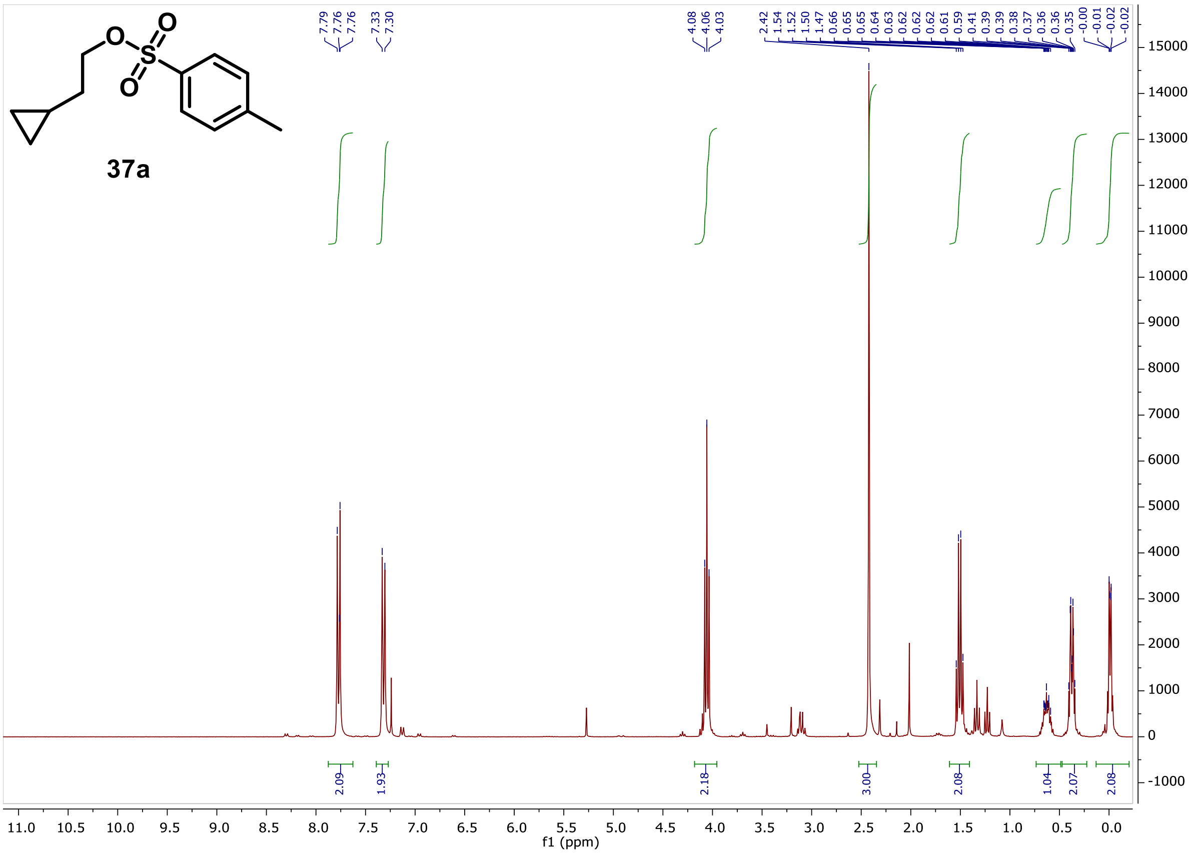

37a

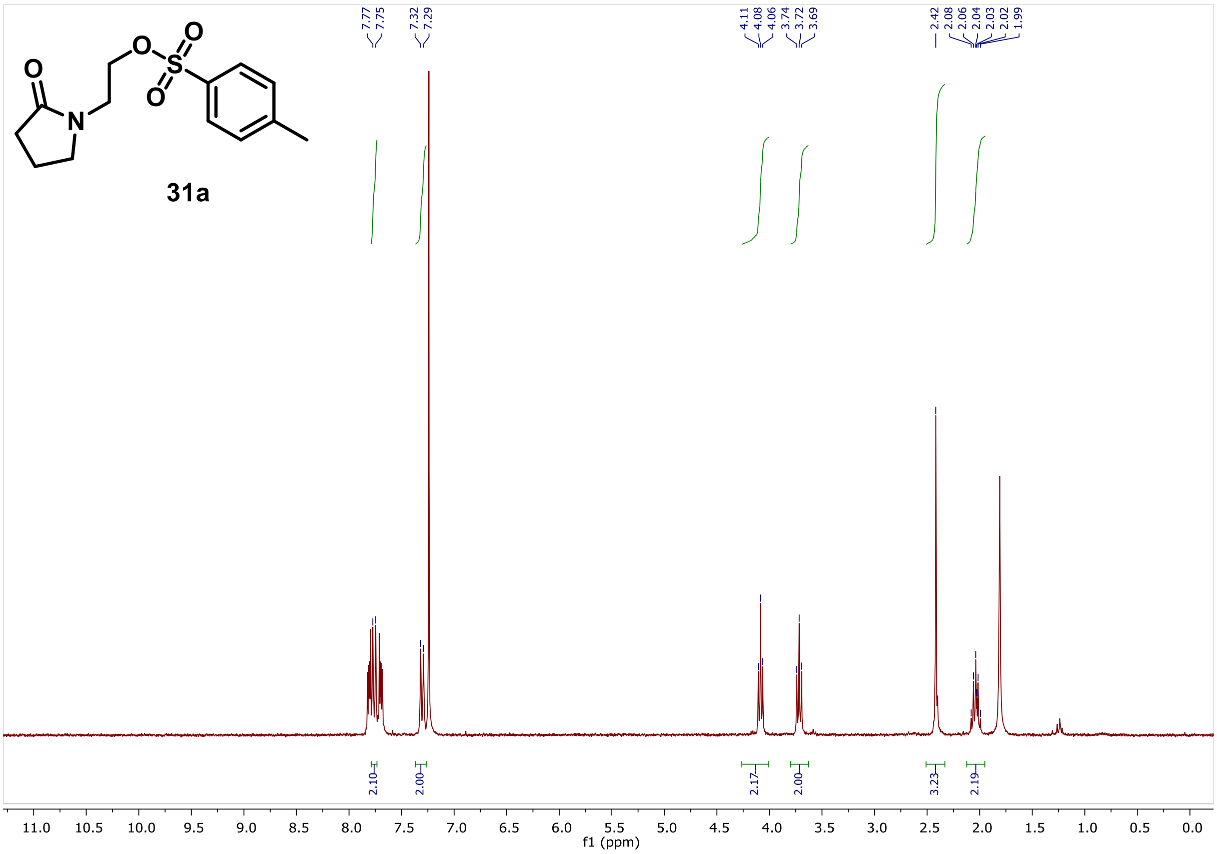
31a
7.77
7.75
7.32
7.29
4.11
4.08
4.06
3.74
3.72
3.69
2.42
2.08
2.06
2.04
2.03
2.02
1.99
2.10
2.00
2.17
2.00
3.23
2.19
11.0 10.5 10.0 9.5 9.0 8.5 8.0 7.5 7.0 6.5 6.0 5.5 5.0 4.5 4.0 3.5 3.0 2.5 2.0 1.5 1.0 0.5 0.0
f1 (ppm)

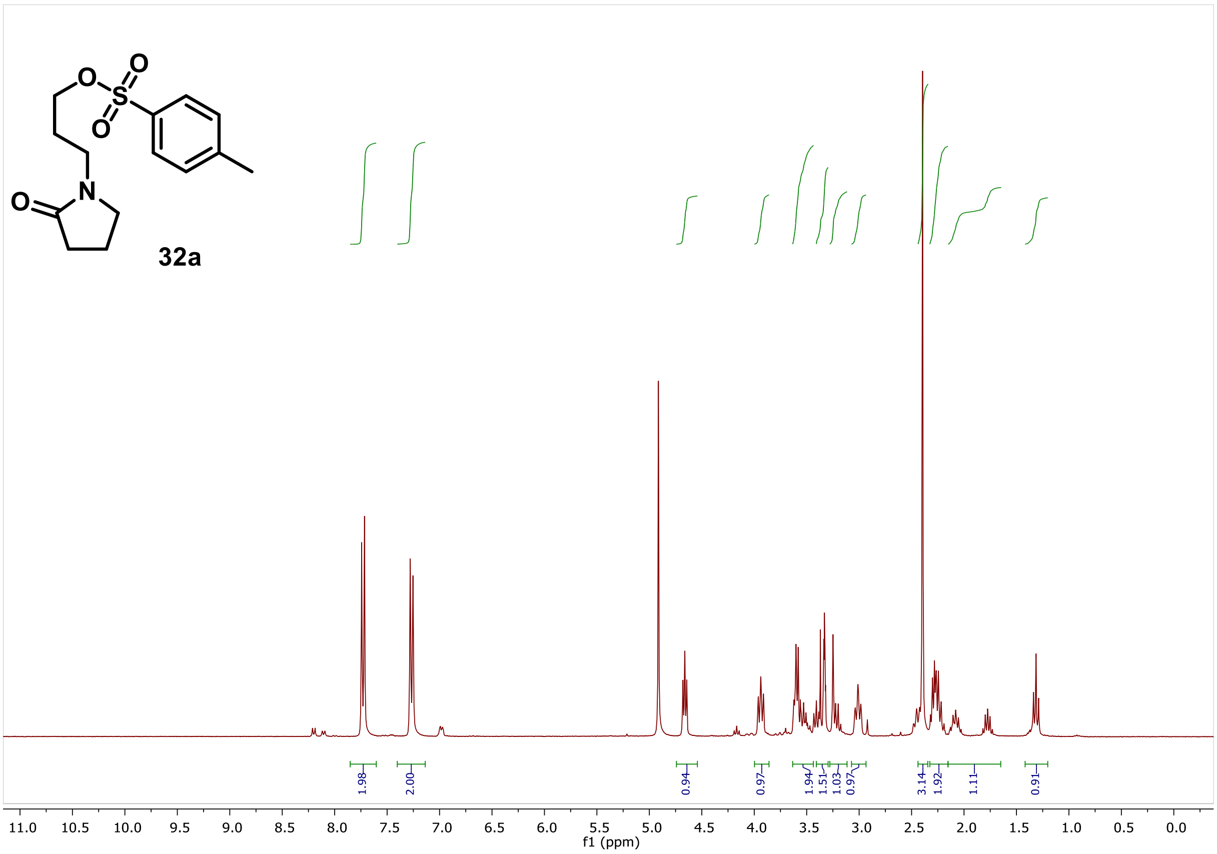
32a
1.98
2.00
0.94
0.97
1.94
1.51
1.03
0.97
3.14
1.92
1.11
0.91
11.0 10.5 10.0 9.5 9.0 8.5 8.0 7.5 7.0 6.5 6.0 5.5 5.0 4.5 4.0 3.5 3.0 2.5 2.0 1.5 1.0 0.5 0.0
f1 (ppm)

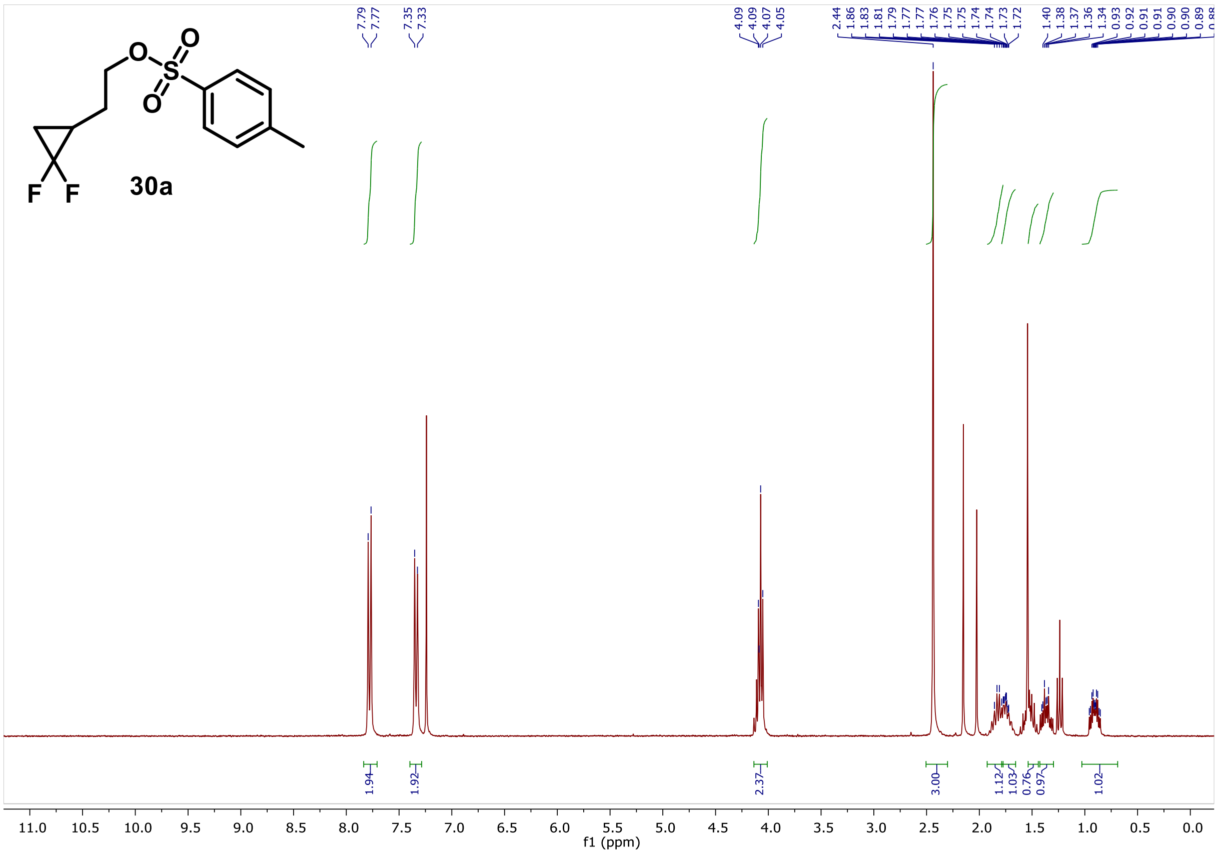
30a
f1 (ppm)

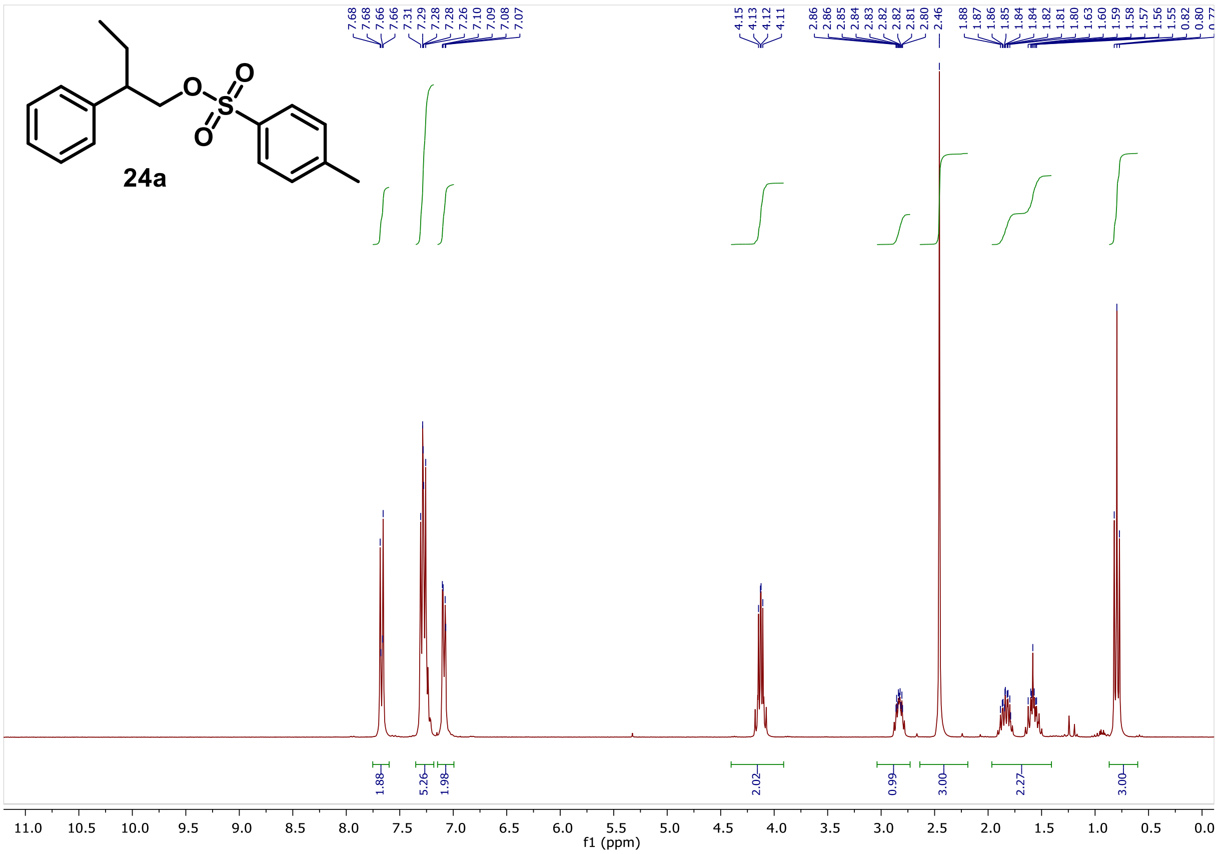
24a
f1 (ppm)

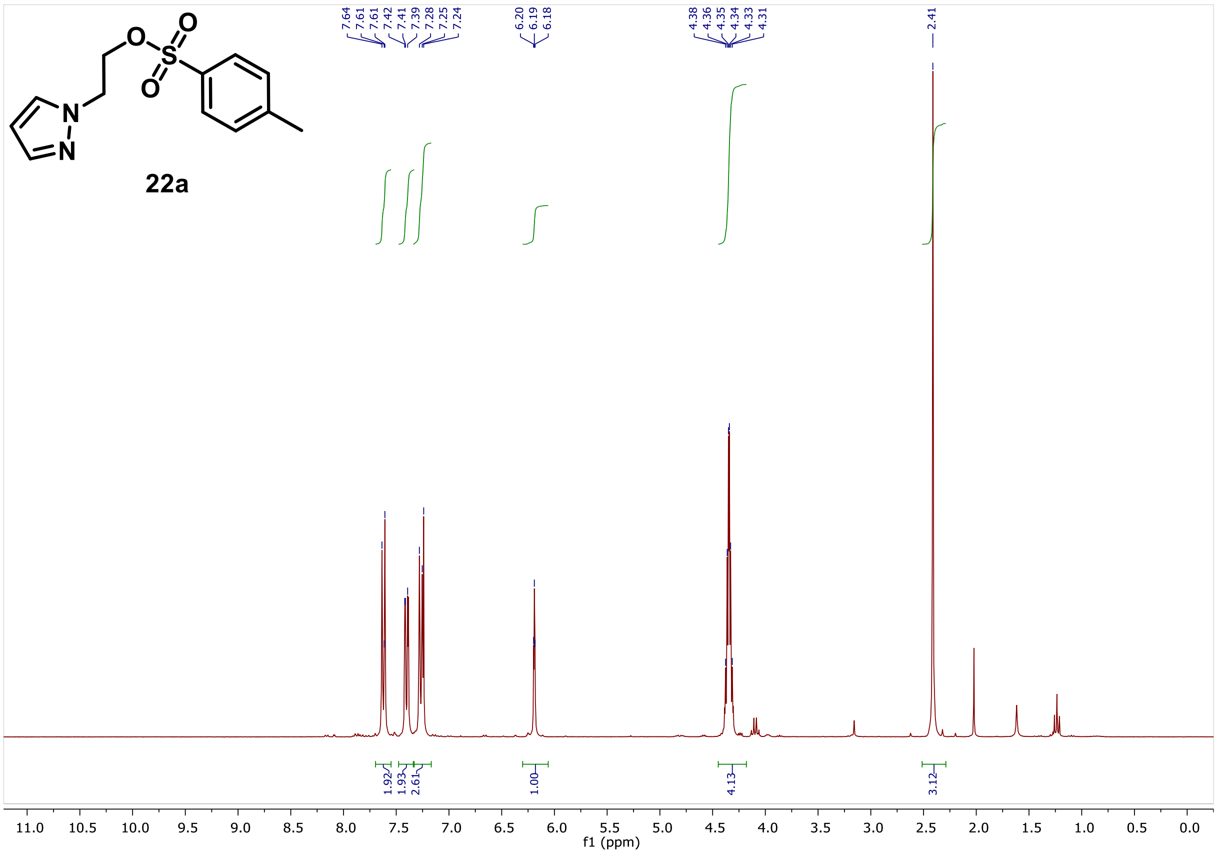
7.64
7.61
7.61
7.42
7.41
7.39
7.28
7.25
7.24
6.20
6.19
6.18
4.38
4.36
4.35
4.34
4.33
4.31
2.41
1.92
1.93
2.61
1.00
4.13
3.12
f1 (ppm)

22a

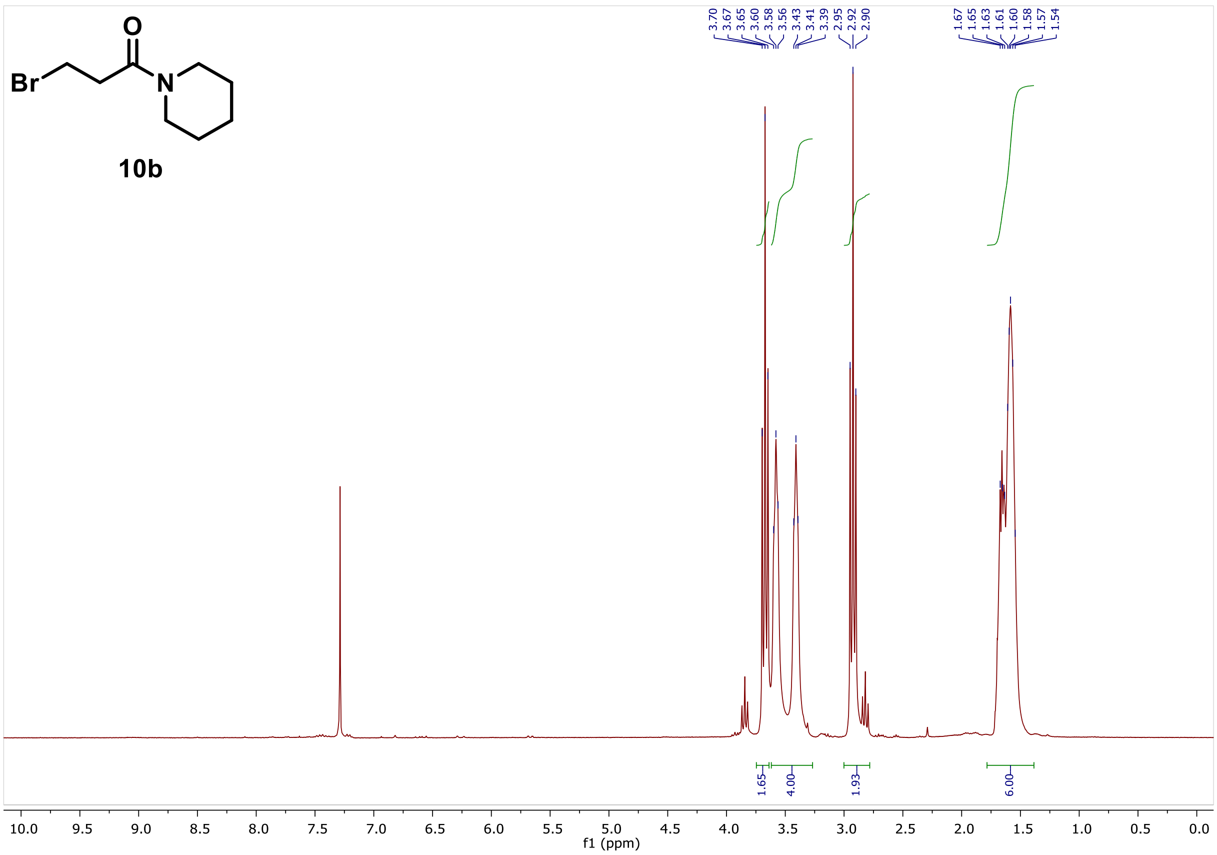
3.70
3.67
3.65
3.60
3.58
3.56
3.43
3.41
3.39
2.95
2.92
2.90
1.67
1.65
1.63
1.61
1.60
1.58
1.57
1.54
1.65
4.00
1.93
6.00
f1 (ppm)

10b

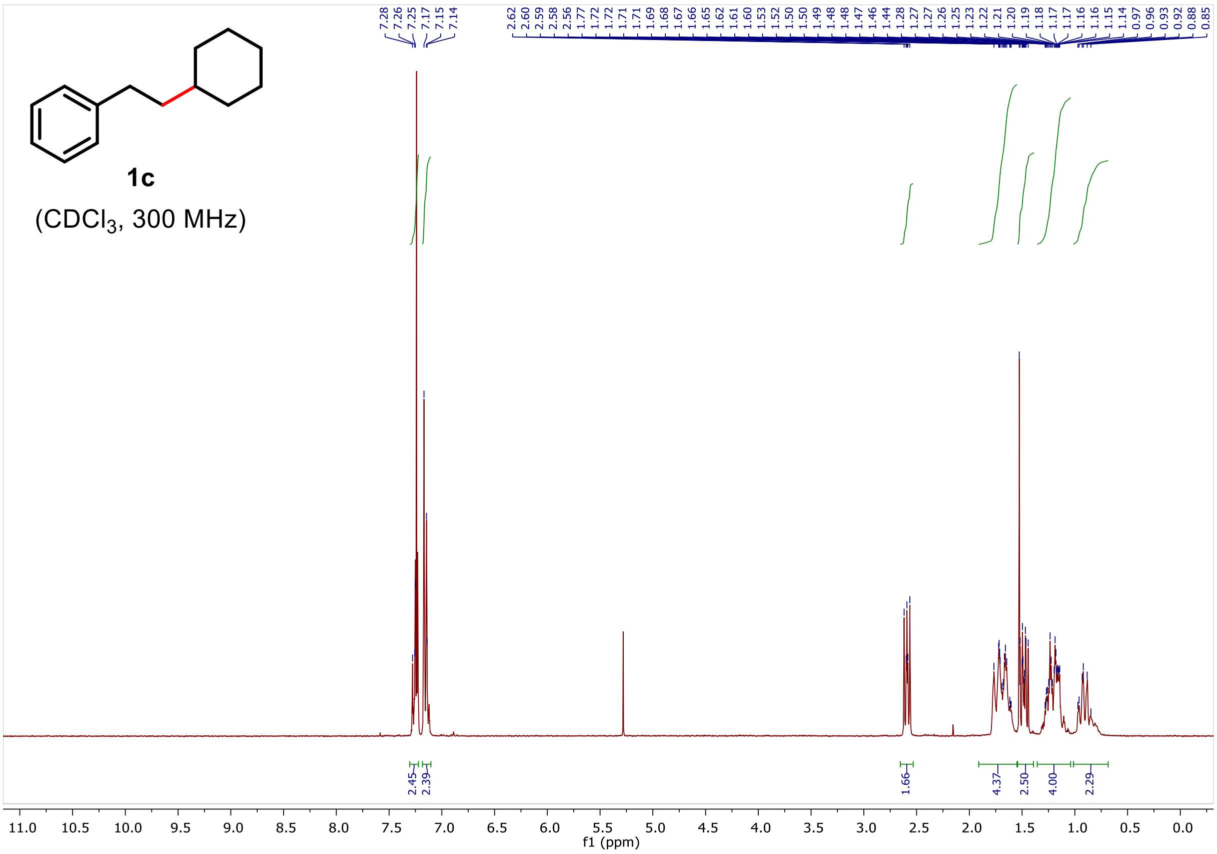
1c
(CDCl$_3$, 300 MHz)
7.28 7.26 7.25 7.17 7.15 7.14
2.62 2.60 2.59 2.58 2.56 1.77 1.72 1.72 1.71 1.71 1.69 1.68 1.67 1.66 1.65 1.62 1.61 1.60 1.53 1.52 1.50 1.50 1.49 1.48 1.48 1.47 1.46 1.44 1.28 1.27 1.27 1.26 1.25 1.23 1.22 1.21 1.20 1.19 1.18 1.17 1.17 1.16 1.16 1.15 1.14 0.97 0.96 0.93 0.92 0.88 0.85
2.45 2.39 1.66 4.37 2.50 4.00 2.29
f1 (ppm)

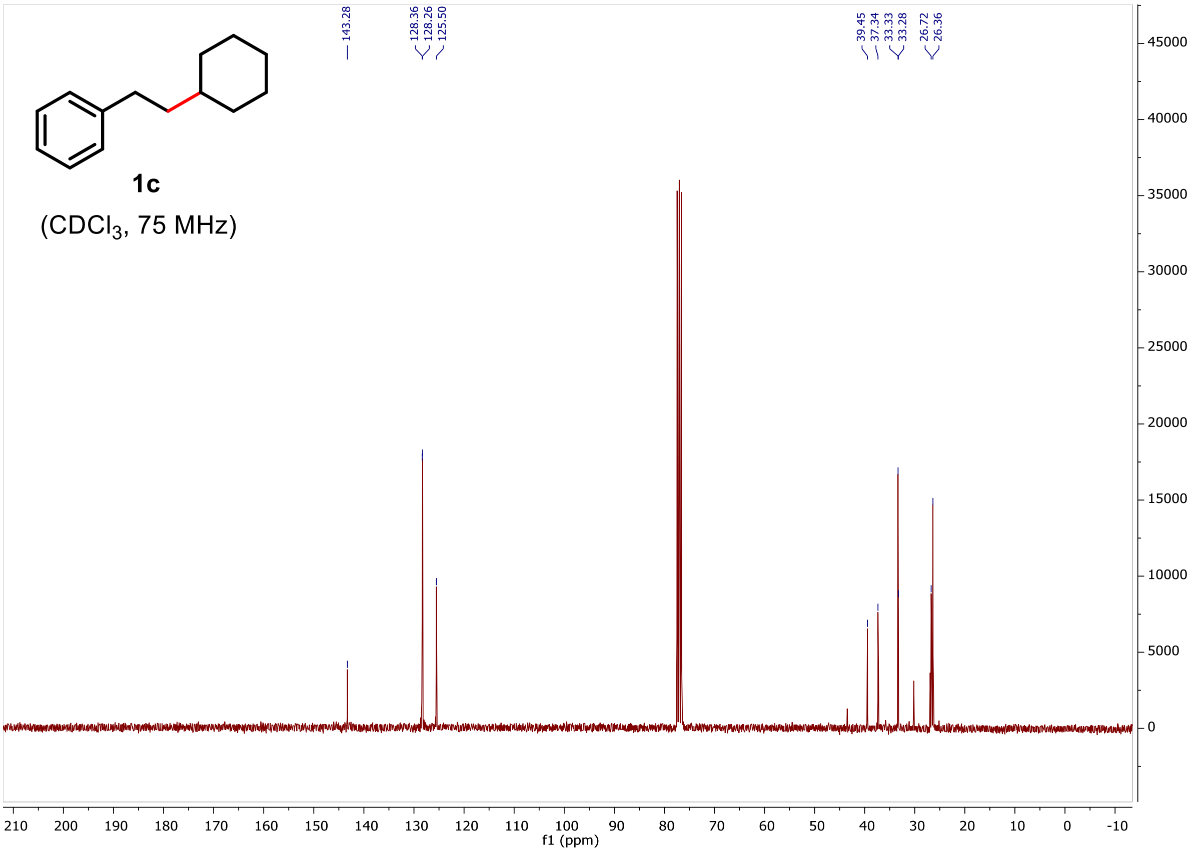
1c
(CDCl$_3$, 75 MHz)
143.28
128.36 128.26 125.50
39.45 37.34 33.33 33.28 26.72 26.36
f1 (ppm)

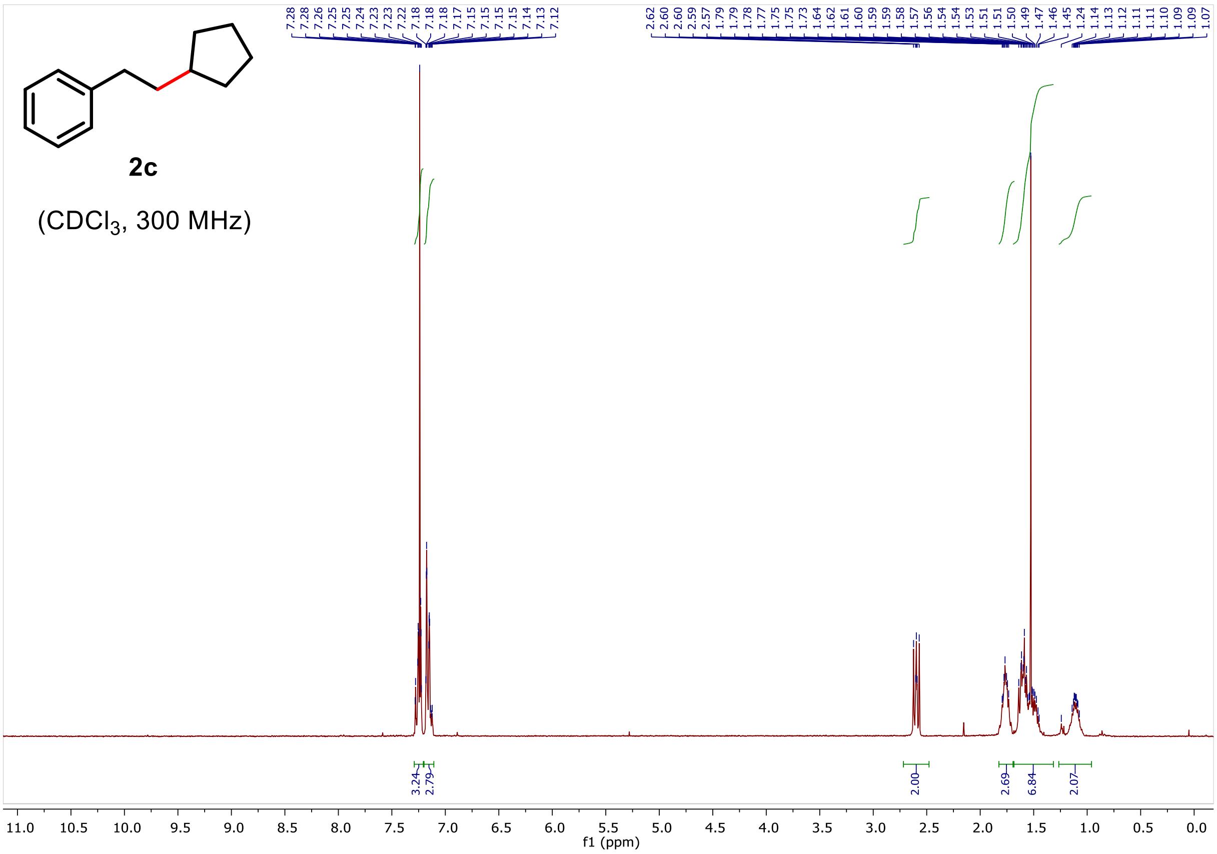
7.28
7.28
7.26
7.25
7.25
7.24
7.23
7.23
7.22
7.18
7.18
7.17
7.15
7.15
7.15
7.15
7.14
7.13
7.12
2.62
2.60
2.60
2.59
2.57
1.79
1.79
1.78
1.77
1.75
1.75
1.73
1.64
1.62
1.61
1.60
1.59
1.59
1.58
1.57
1.56
1.54
1.54
1.53
1.51
1.51
1.50
1.49
1.47
1.46
1.45
1.24
1.14
1.13
1.12
1.11
1.11
1.10
1.09
1.09
1.07
2c
(CDCl3, 300 MHz)
3.24
2.79
2.00
2.69
6.84
2.07
f1 (ppm)

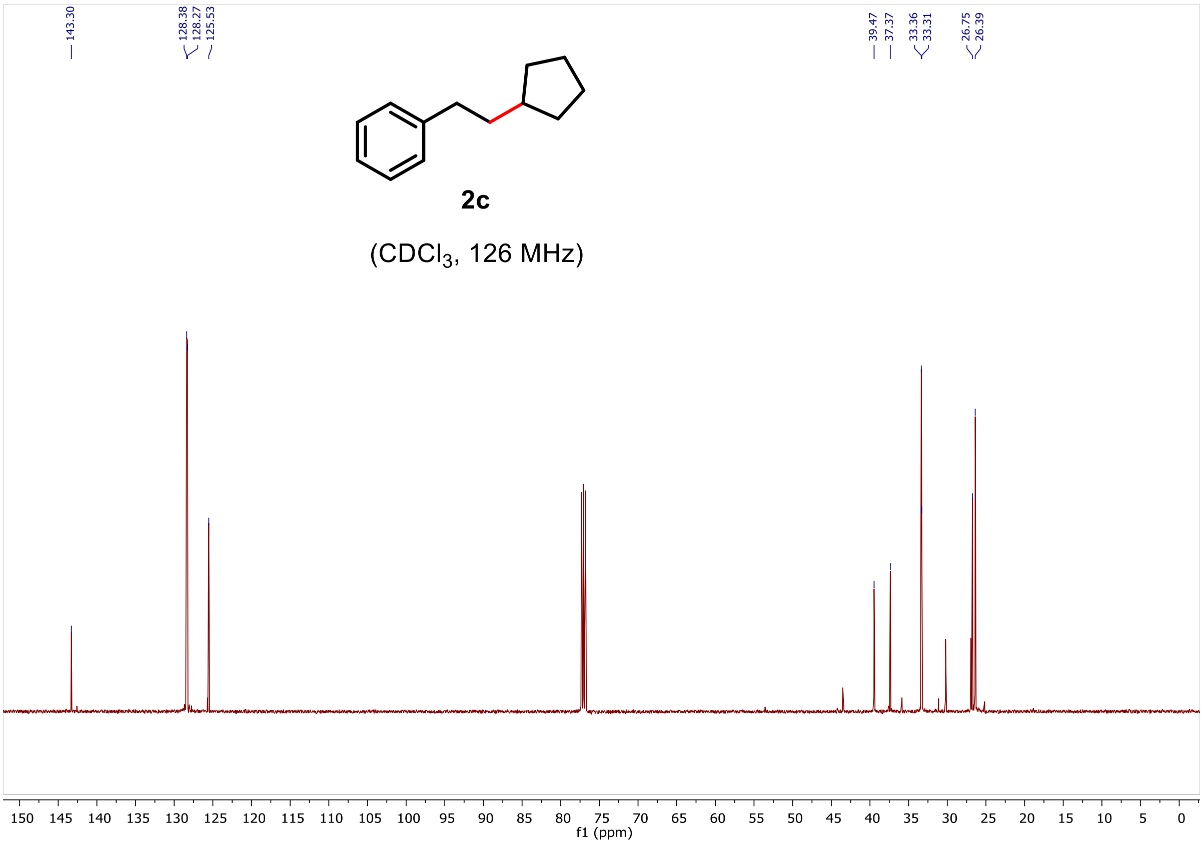
143.30
128.38
128.27
125.53
39.47
37.37
33.36
33.31
26.75
26.39
2c
(CDCl3, 126 MHz)
f1 (ppm)

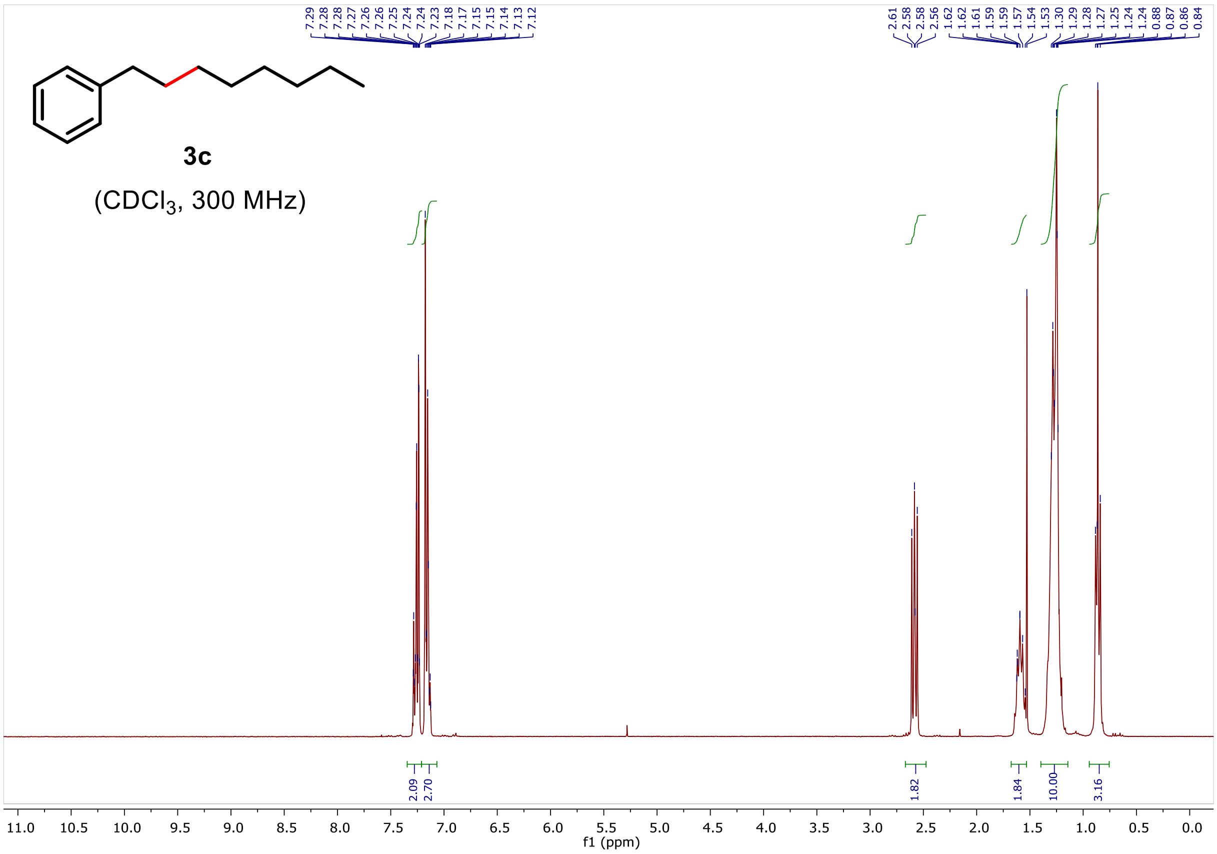
3c
(CDCl3, 300 MHz)
f1 (ppm)

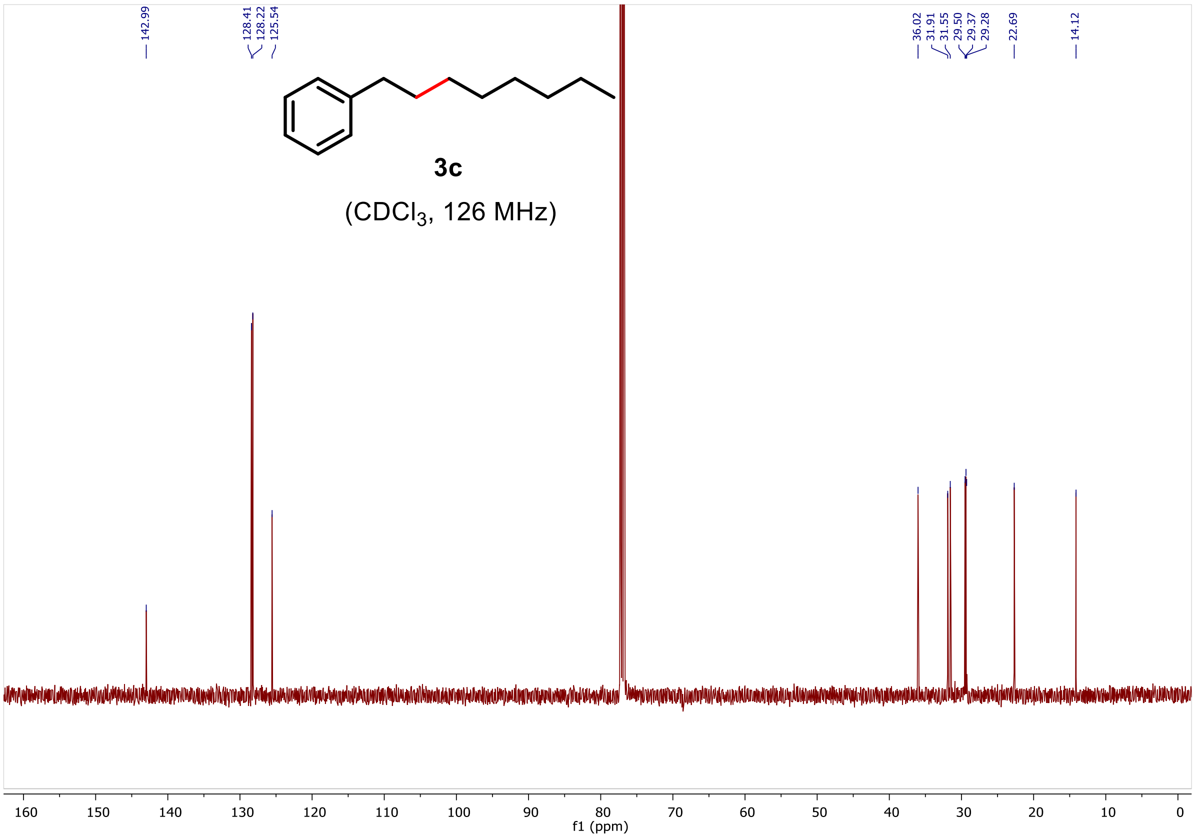
3c
(CDCl3, 126 MHz)
f1 (ppm)

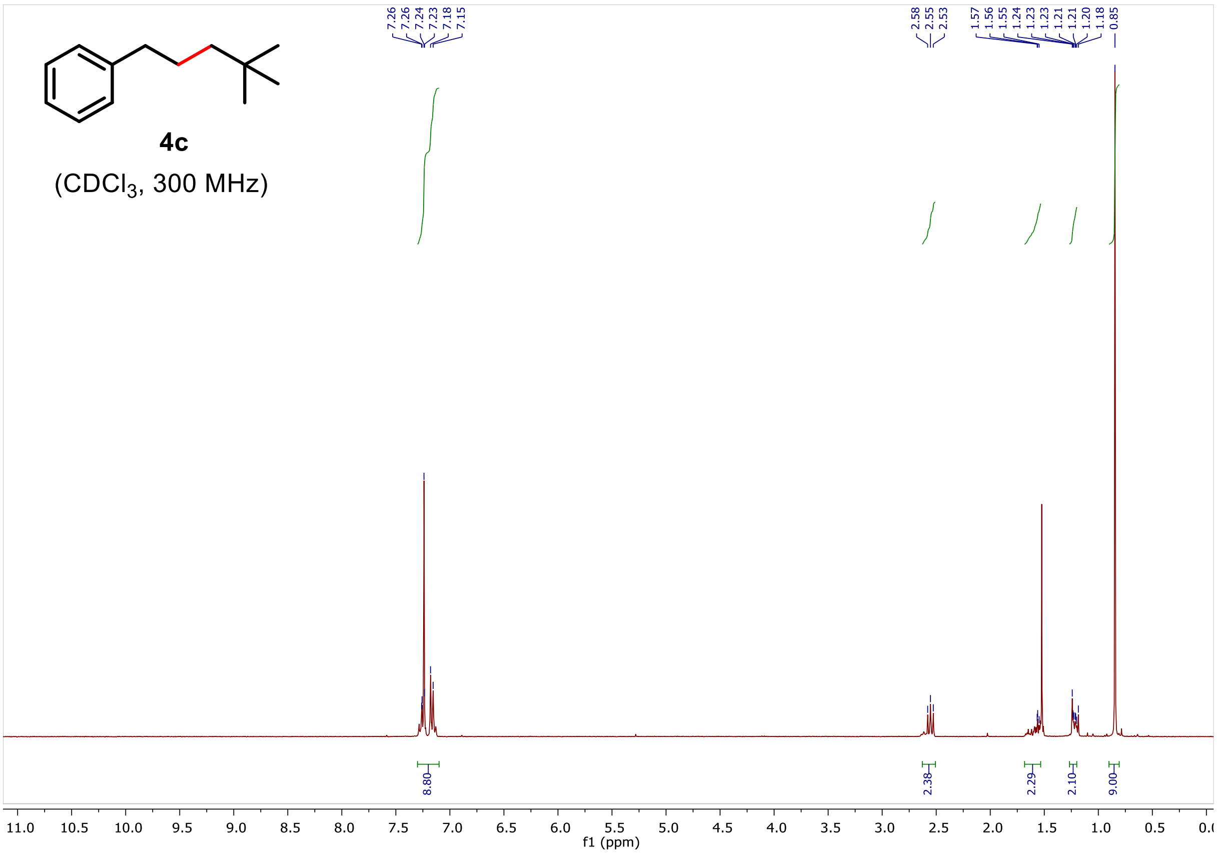
4c
(CDCl$_3$, 300 MHz)
7.26 7.26 7.24 7.23 7.18 7.15
2.58 2.55 2.53
1.57 1.56 1.55 1.24 1.23 1.23 1.21 1.21 1.20 1.18
0.85
8.80
2.38
2.29
2.10
9.00
11.0 10.5 10.0 9.5 9.0 8.5 8.0 7.5 7.0 6.5 6.0 5.5 5.0 4.5 4.0 3.5 3.0 2.5 2.0 1.5 1.0 0.5
f1 (ppm)

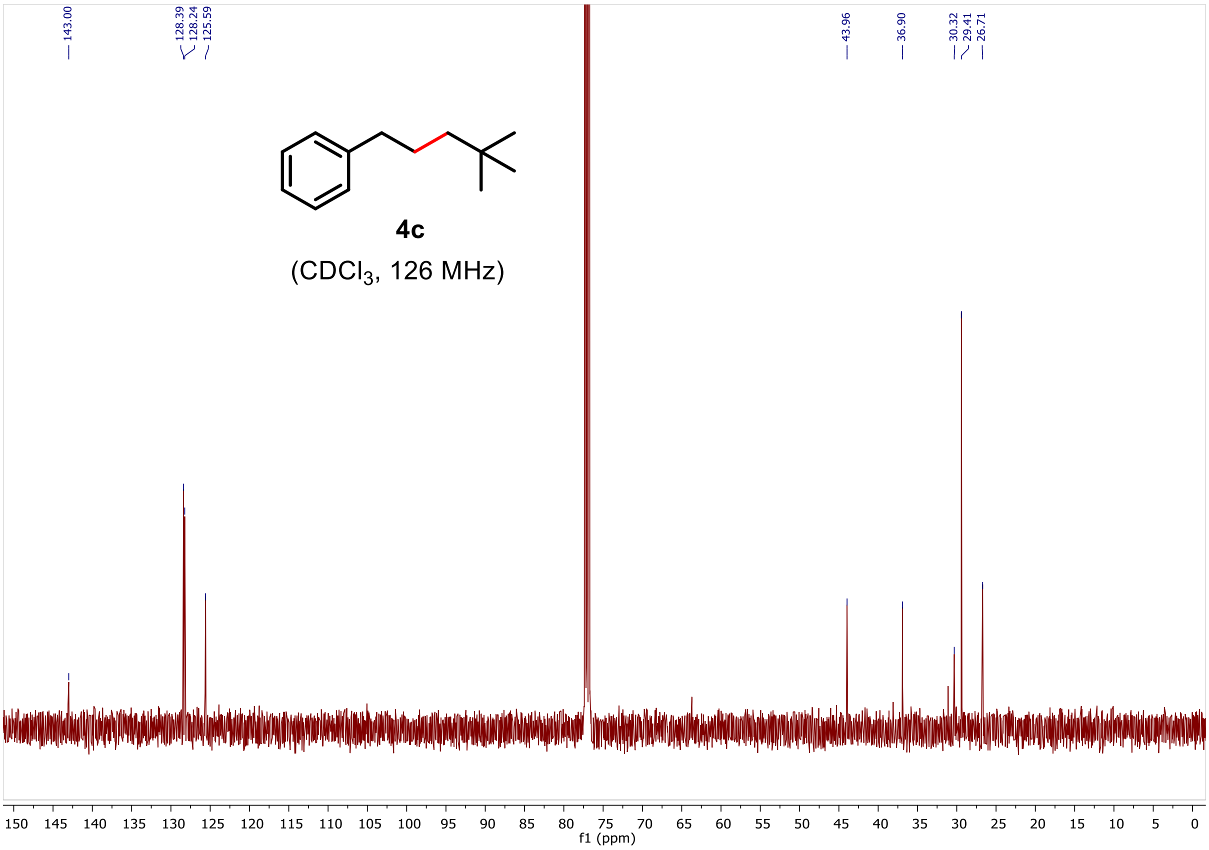
143.00
128.39 128.24 125.59
43.96
36.90
30.32 29.41 26.71
4c
(CDCl$_3$, 126 MHz)
150 145 140 135 130 125 120 115 110 105 100 95 90 85 80 75 70 65 60 55 50 45 40 35 30 25 20 15 10 5 0
f1 (ppm)

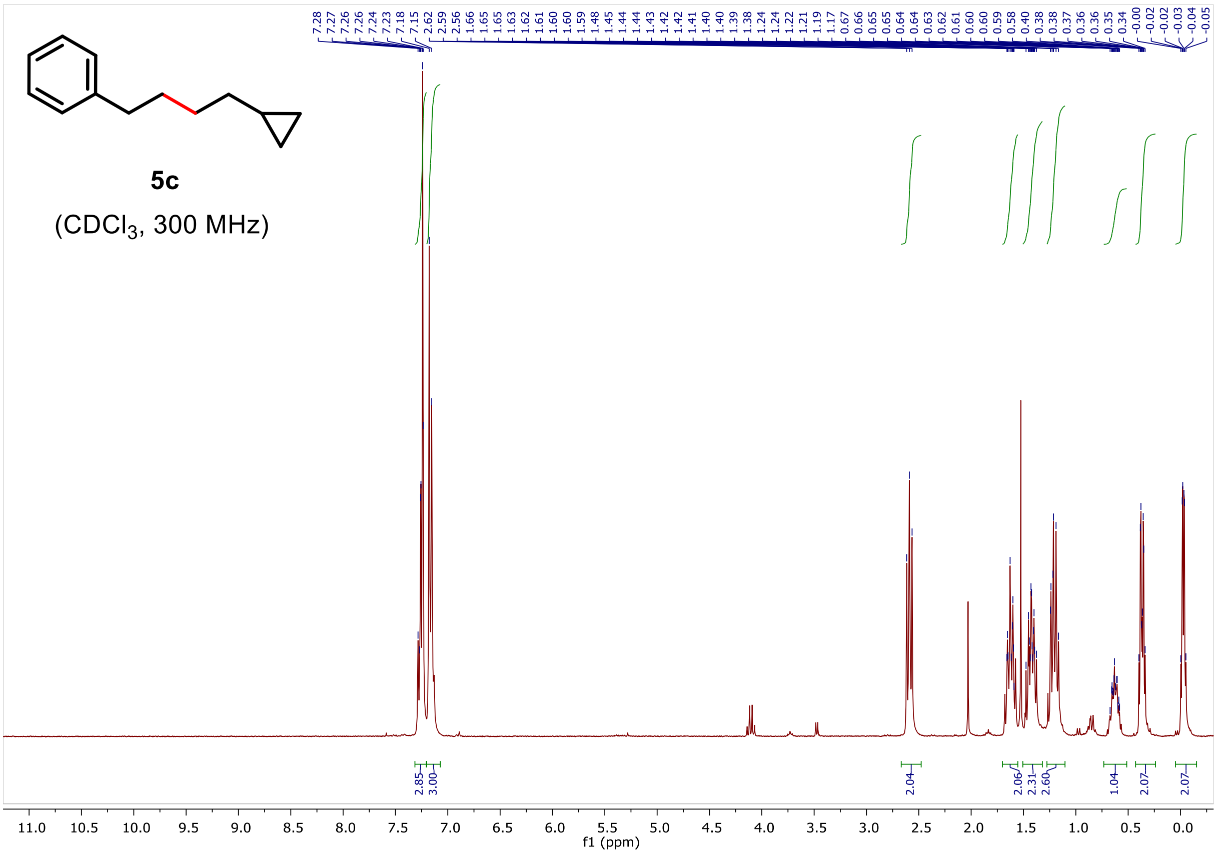
5c
(CDCl3, 300 MHz)
2.85
3.00
2.04
2.06
2.31
2.60
1.04
2.07
2.07
f1 (ppm)

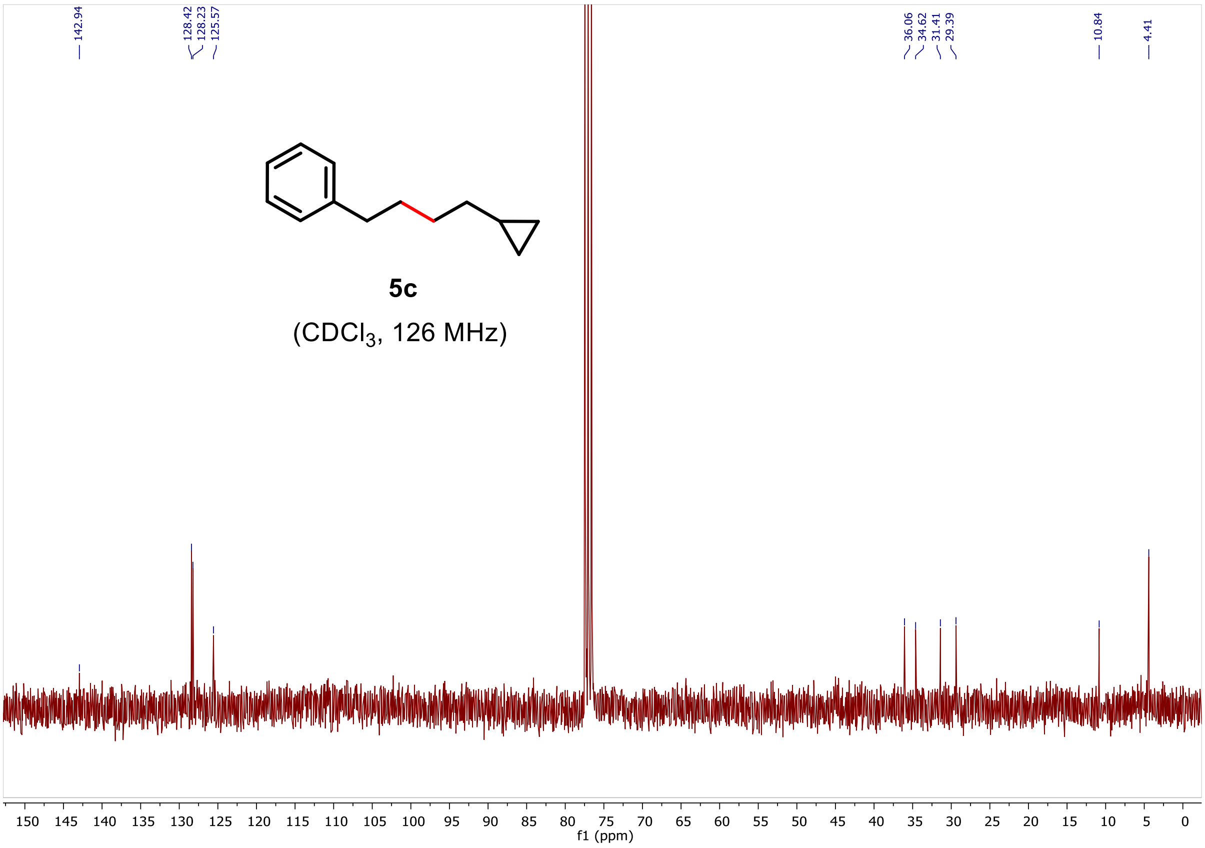
142.94
128.42
128.23
125.57
36.06
34.62
31.41
29.39
10.84
4.41
5c
(CDCl3, 126 MHz)
f1 (ppm)

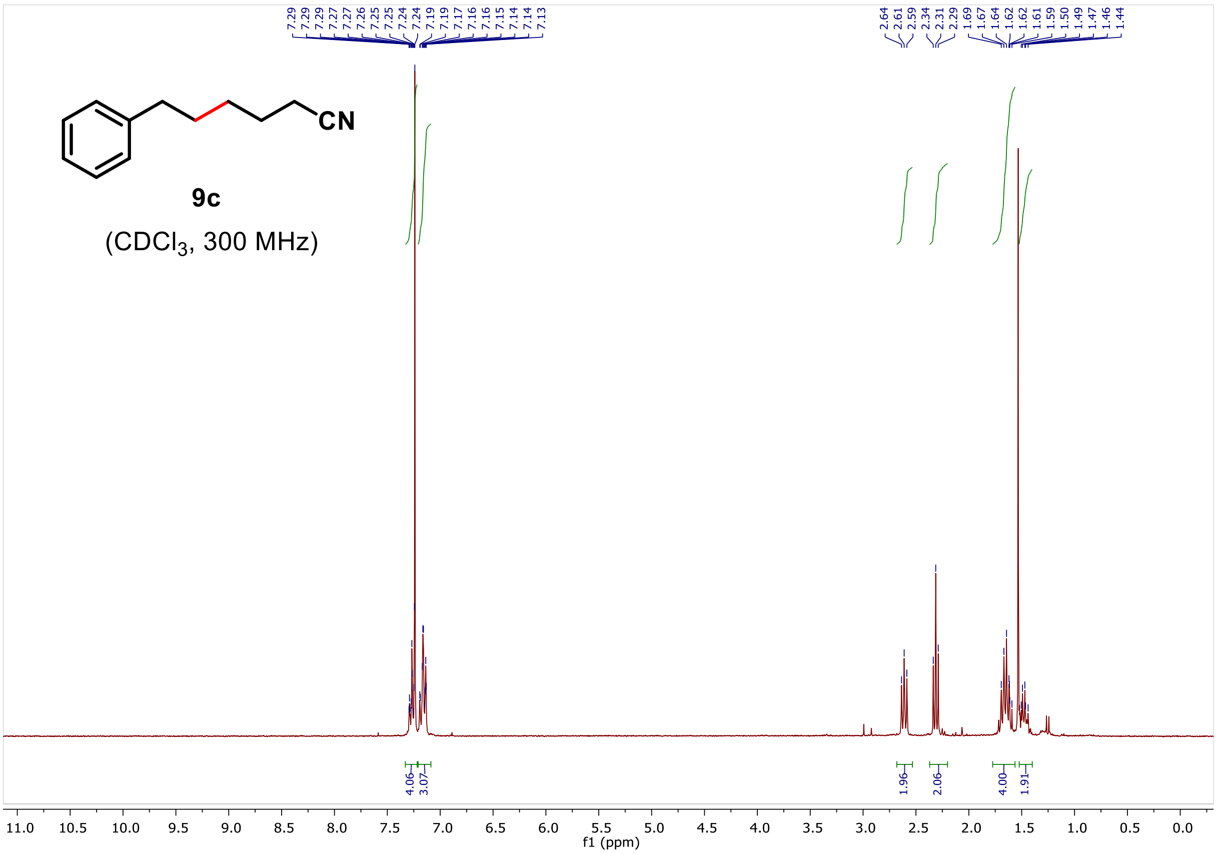
7.29
7.29
7.29
7.27
7.27
7.26
7.25
7.25
7.24
7.24
7.19
7.19
7.17
7.16
7.16
7.15
7.14
7.14
7.13
2.64
2.61
2.59
2.34
2.31
2.29
1.69
1.67
1.64
1.62
1.62
1.61
1.59
1.50
1.49
1.47
1.46
1.44
CN
9c
(CDCl3, 300 MHz)
4.06
3.07
1.96
2.06
4.00
1.91
f1 (ppm)

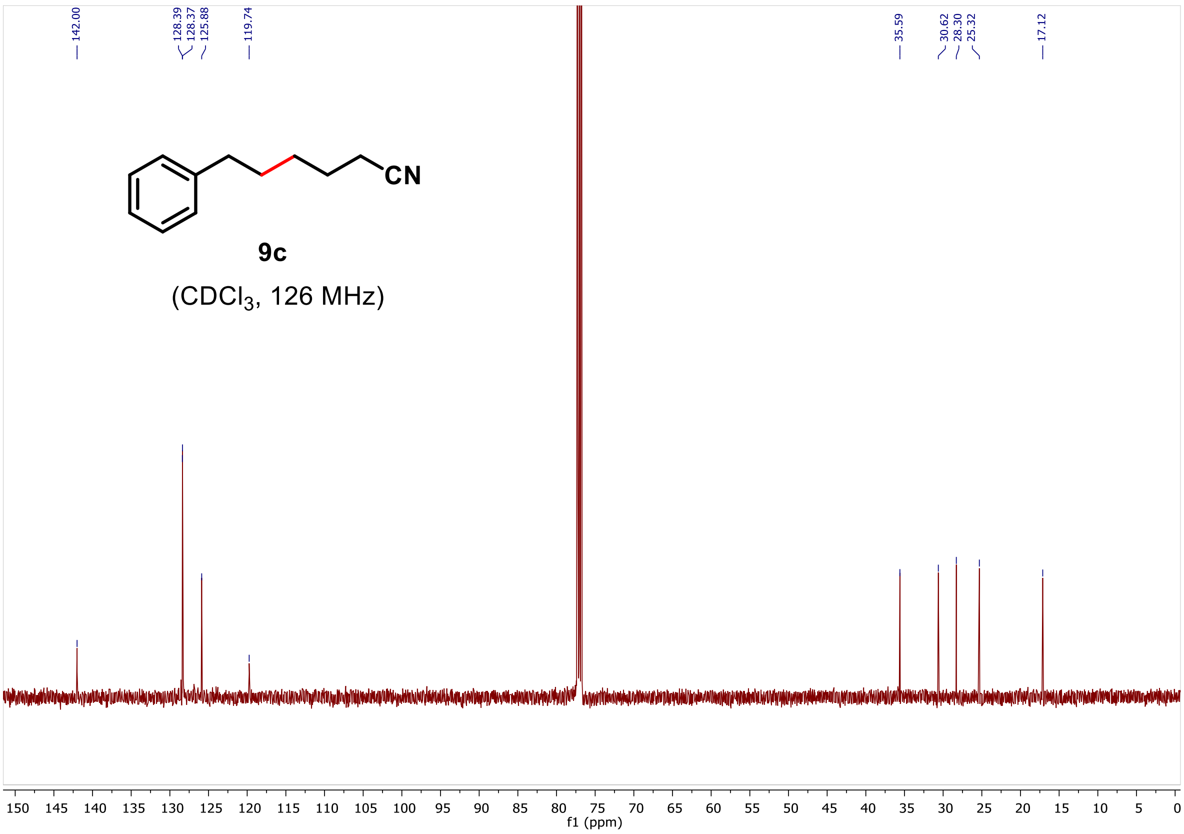
142.00
128.39
128.37
125.88
119.74
35.59
30.62
28.30
25.32
17.12
CN
9c
(CDCl3, 126 MHz)
f1 (ppm)

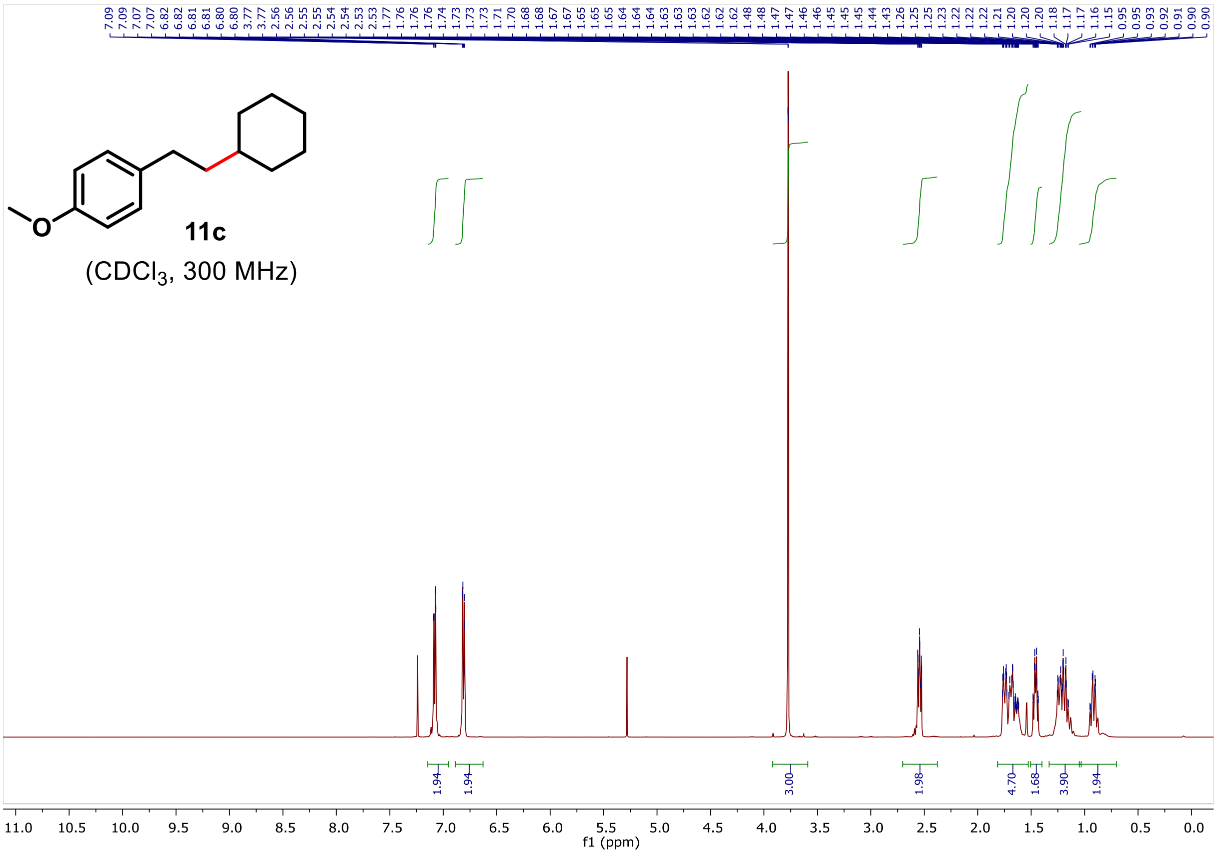
11c
(CDCl3, 300 MHz)
7.09 7.09 7.07 7.07 6.82 6.81 6.81 6.80 6.80 3.77 3.77 2.56 2.56 2.55 2.55 2.54 2.54 2.53 2.53 1.77 1.76 1.76 1.74 1.73 1.73 1.73 1.71 1.70 1.68 1.68 1.67 1.67 1.65 1.65 1.65 1.64 1.64 1.64 1.63 1.63 1.63 1.62 1.62 1.62 1.48 1.48 1.48 1.47 1.47 1.46 1.46 1.45 1.45 1.45 1.44 1.43 1.26 1.25 1.25 1.23 1.22 1.22 1.22 1.21 1.20 1.20 1.20 1.18 1.17 1.17 1.16 1.15 0.95 0.95 0.93 0.92 0.91 0.90 0.90
1.94
1.94
3.00
1.98
4.70
1.68
3.90
1.94
11.0 10.5 10.0 9.5 9.0 8.5 8.0 7.5 7.0 6.5 6.0 5.5 5.0 4.5 4.0 3.5 3.0 2.5 2.0 1.5 1.0 0.5 0.0
f1 (ppm)

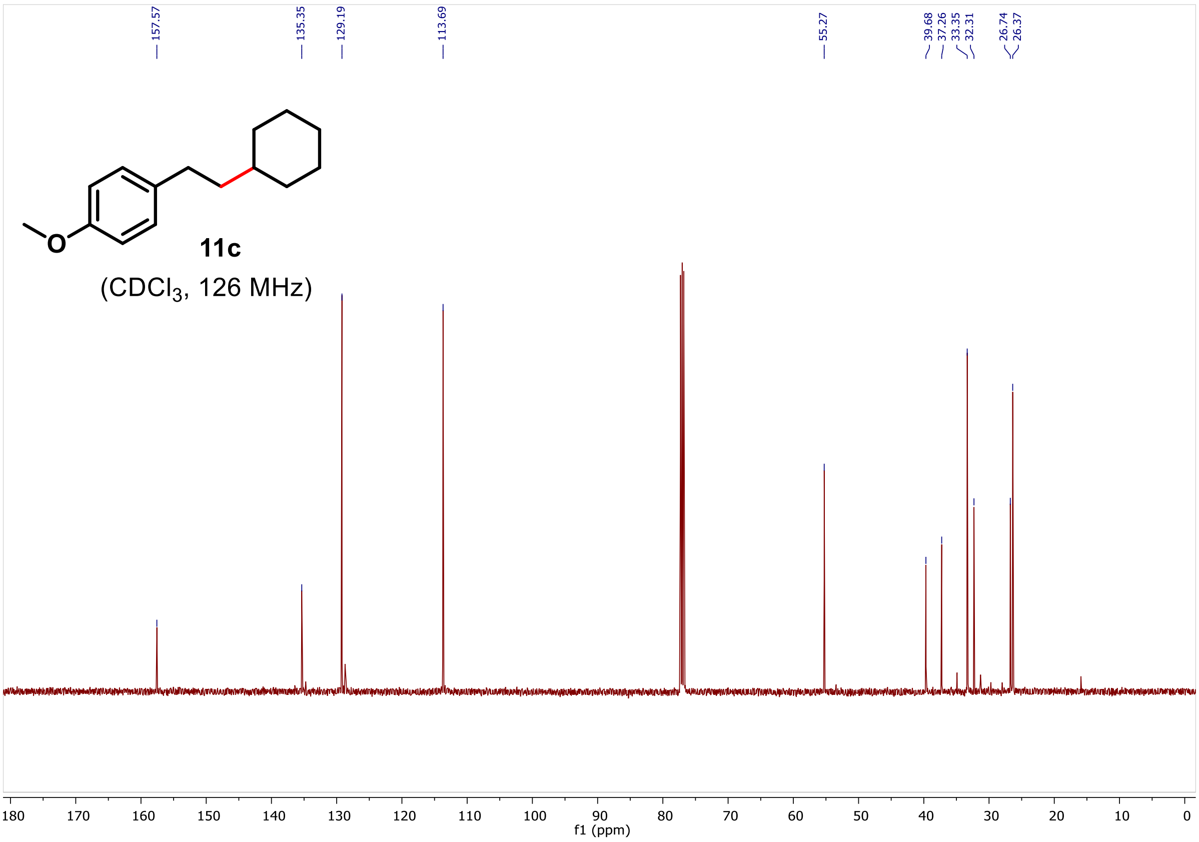
11c
(CDCl3, 126 MHz)
157.57
135.35
129.19
113.69
55.27
39.68
37.26
33.35
32.31
26.74
26.37
180 170 160 150 140 130 120 110 100 90 80 70 60 50 40 30 20 10 0
f1 (ppm)

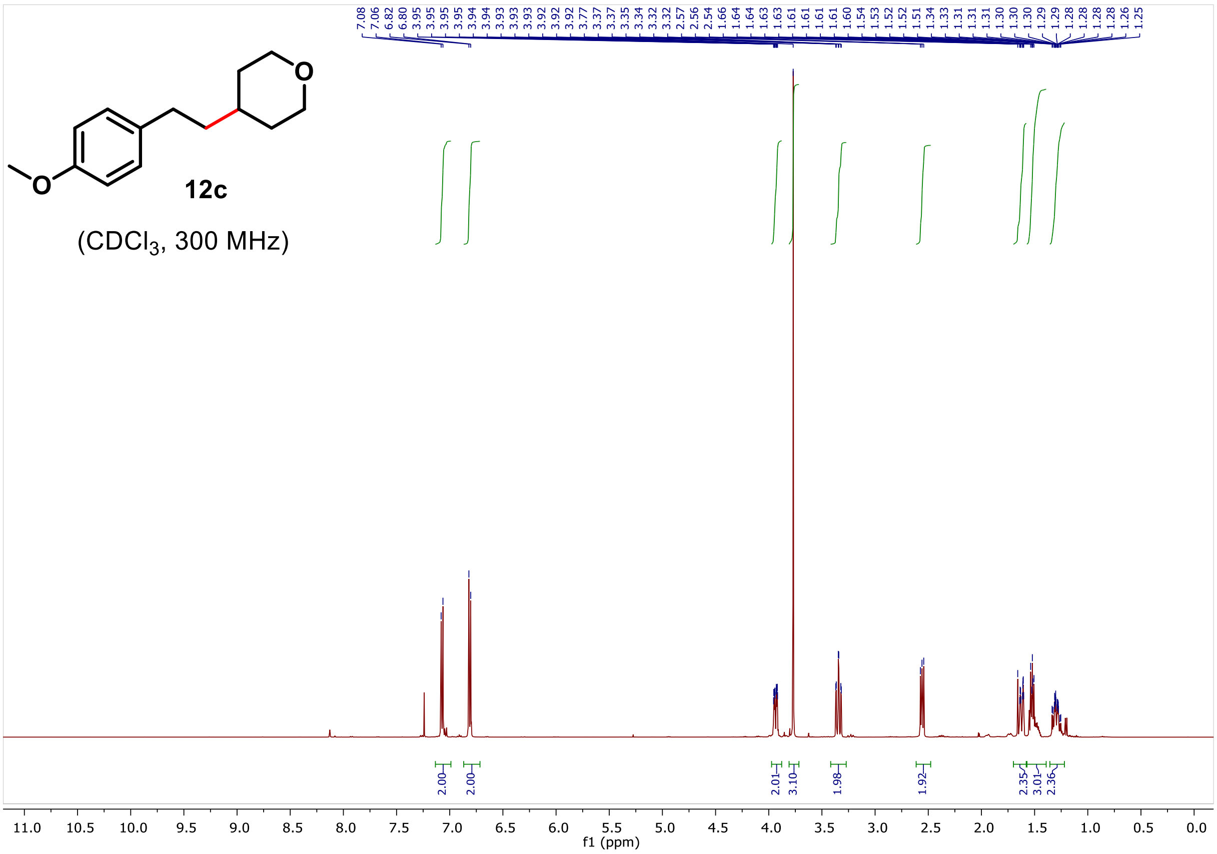
7.08 7.06 6.82 6.80 3.95 3.95 3.95 3.95 3.94 3.94 3.93 3.93 3.93 3.92 3.92 3.92 3.77 3.37 3.37 3.35 3.34 3.32 3.32 2.57 2.56 2.54 1.66 1.64 1.63 1.63 1.61 1.61 1.61 1.61 1.60 1.54 1.53 1.52 1.52 1.51 1.34 1.33 1.31 1.31 1.31 1.30 1.30 1.30 1.29 1.29 1.28 1.28 1.28 1.26 1.25
12c
(CDCl$_3$, 300 MHz)
2.00 2.00 2.01 3.10 1.98 1.92 2.35 3.01 2.36
11.0 10.5 10.0 9.5 9.0 8.5 8.0 7.5 7.0 6.5 6.0 5.5 5.0 4.5 4.0 3.5 3.0 2.5 2.0 1.5 1.0 0.5 0.0
f1 (ppm)

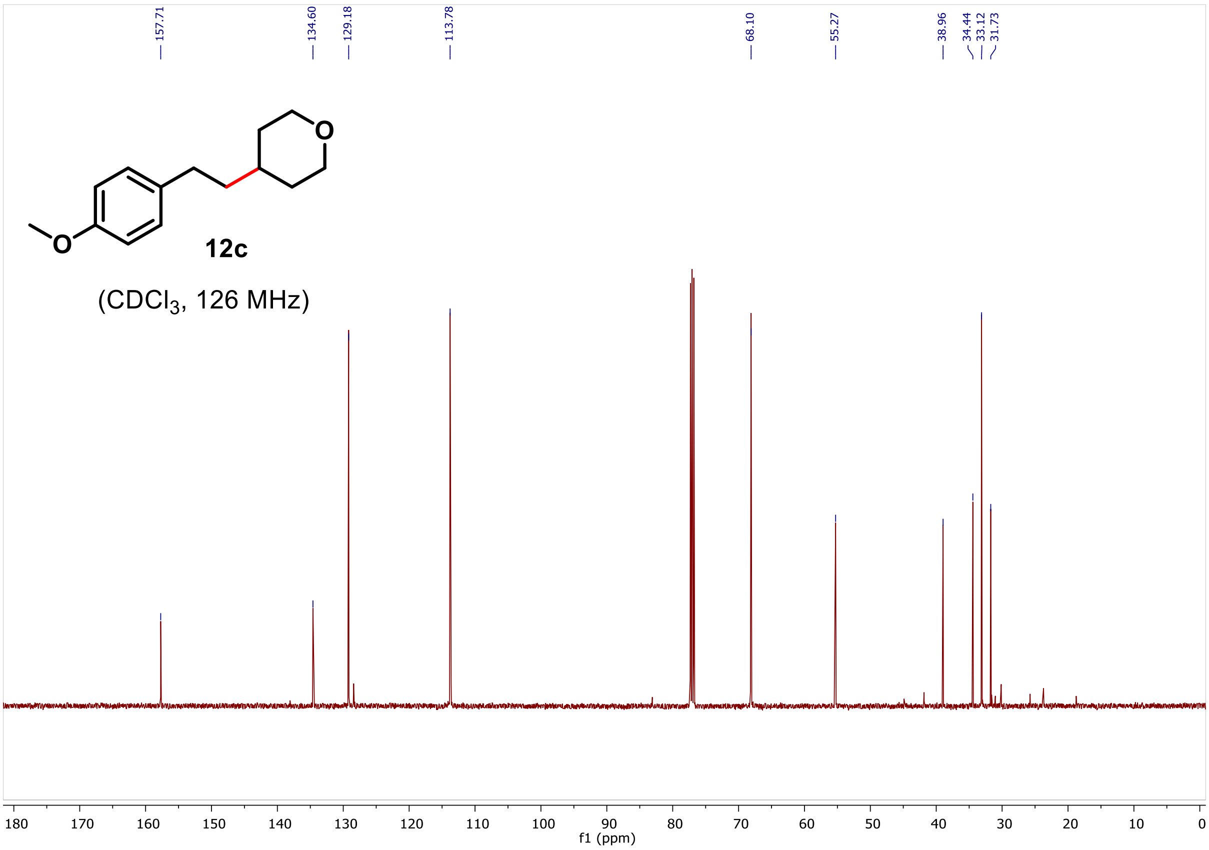
157.71 134.60 129.18 113.78 68.10 55.27 38.96 34.44 33.12 31.73
12c
(CDCl$_3$, 126 MHz)
180 170 160 150 140 130 120 110 100 90 80 70 60 50 40 30 20 10 0
f1 (ppm)

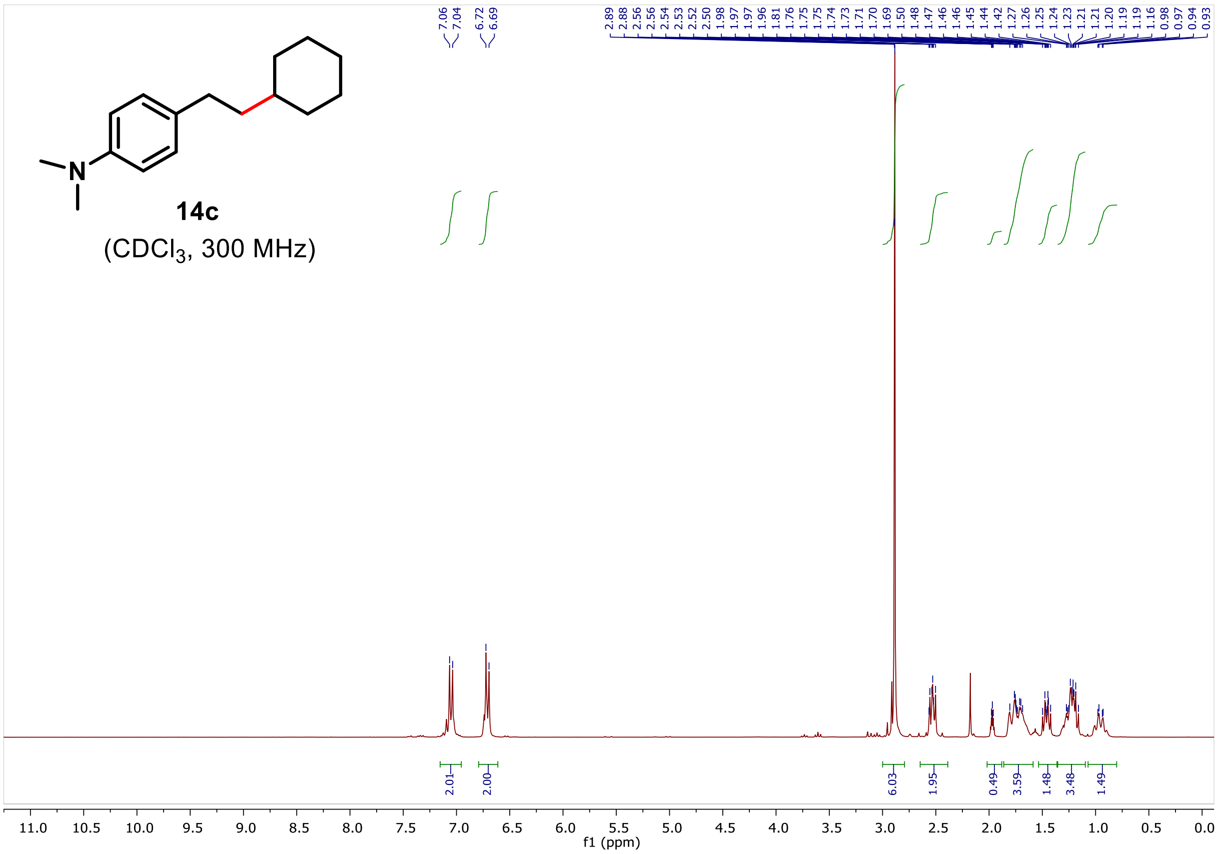
14c
(CDCl3, 300 MHz)
f1 (ppm)

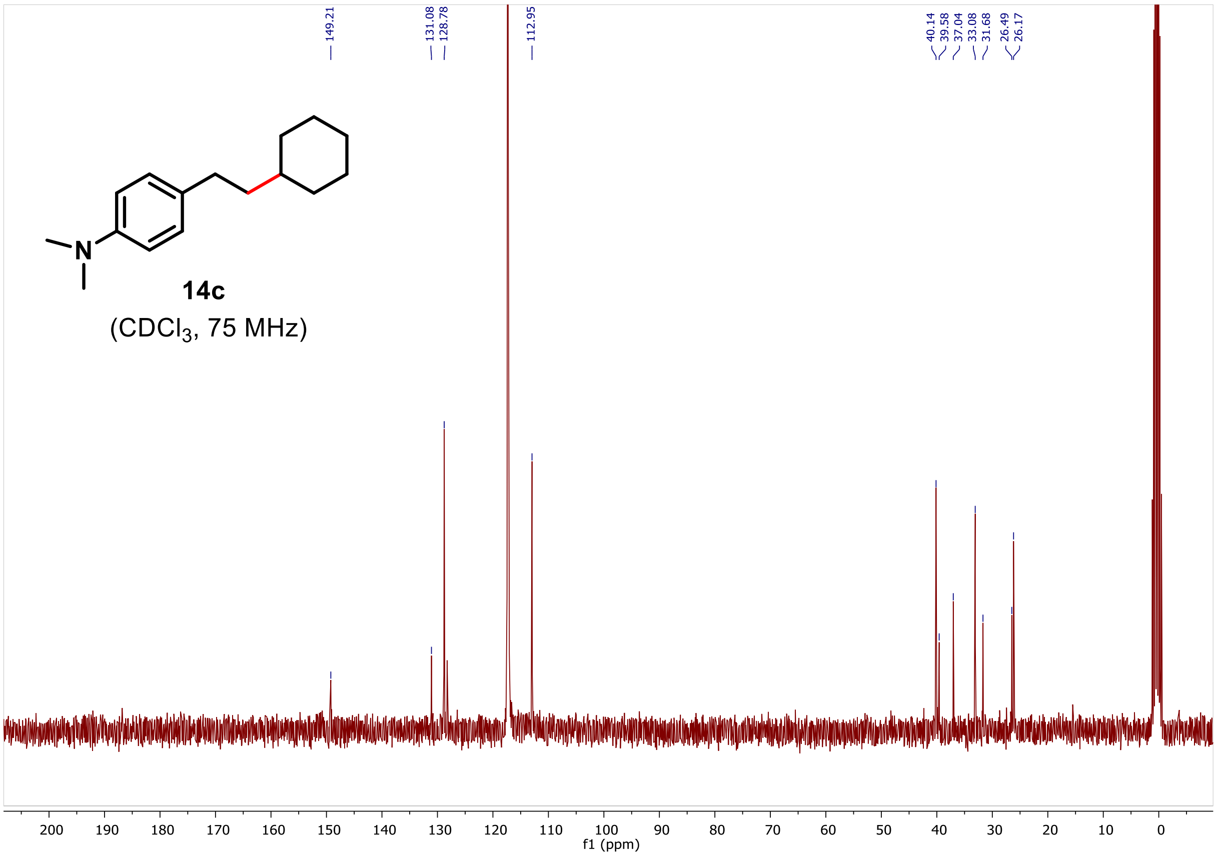
14c
(CDCl3, 75 MHz)
f1 (ppm)

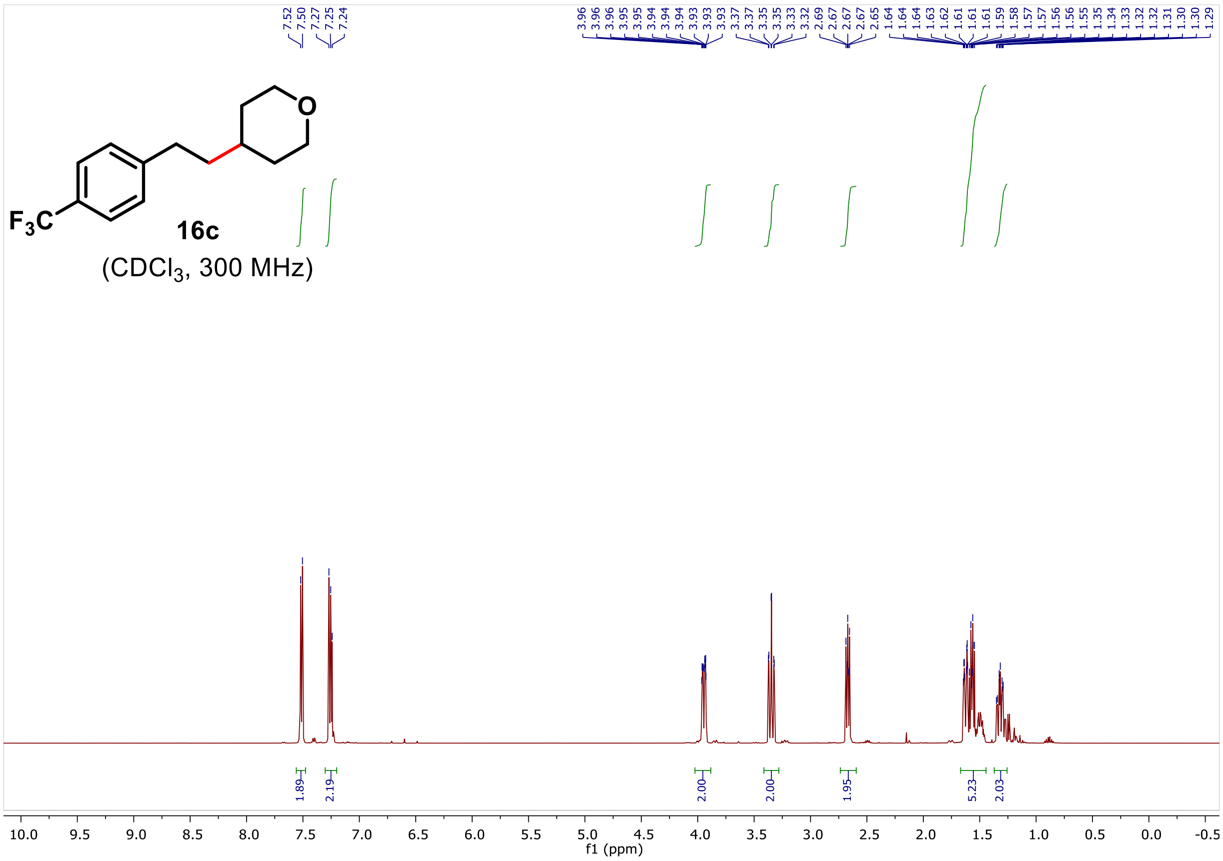
F3C
16c
(CDCl3, 300 MHz)
f1 (ppm)

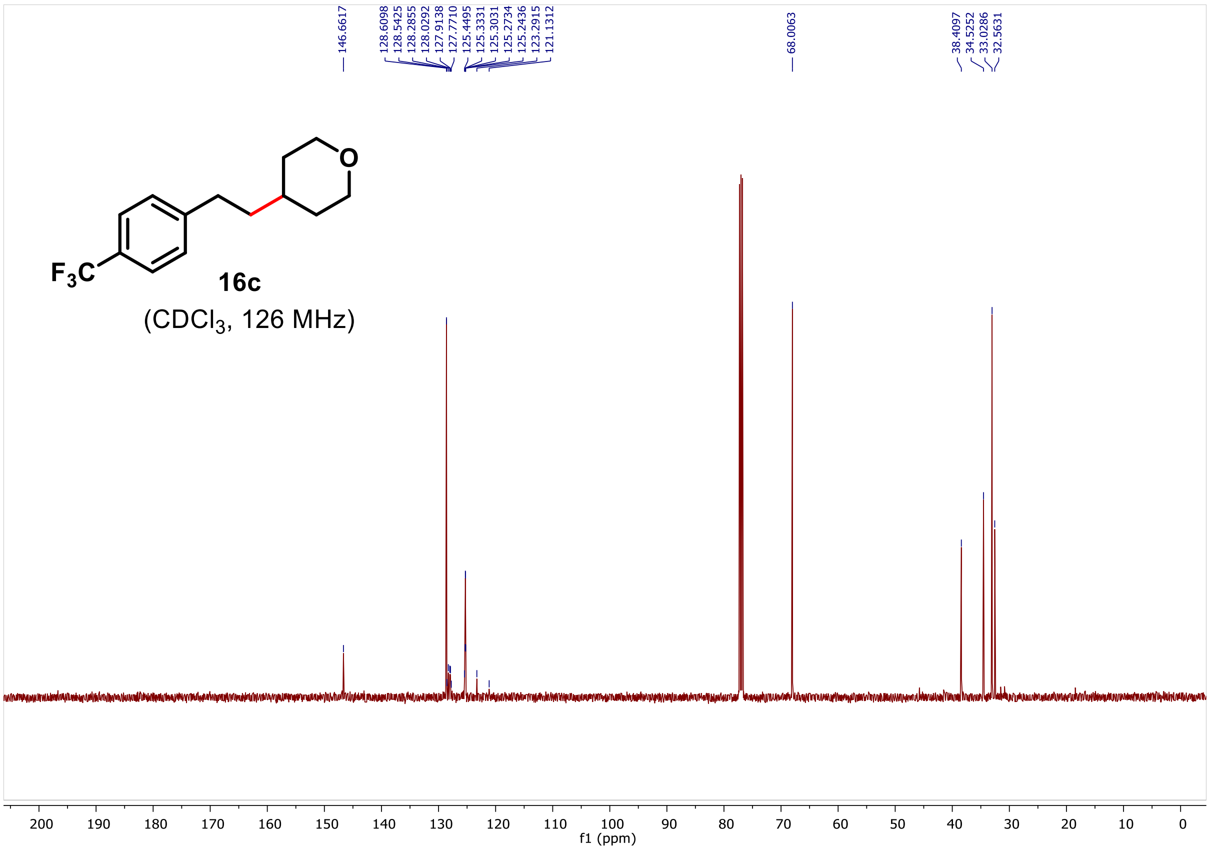
F3C
16c
(CDCl3, 126 MHz)
146.6617
68.0063
f1 (ppm)

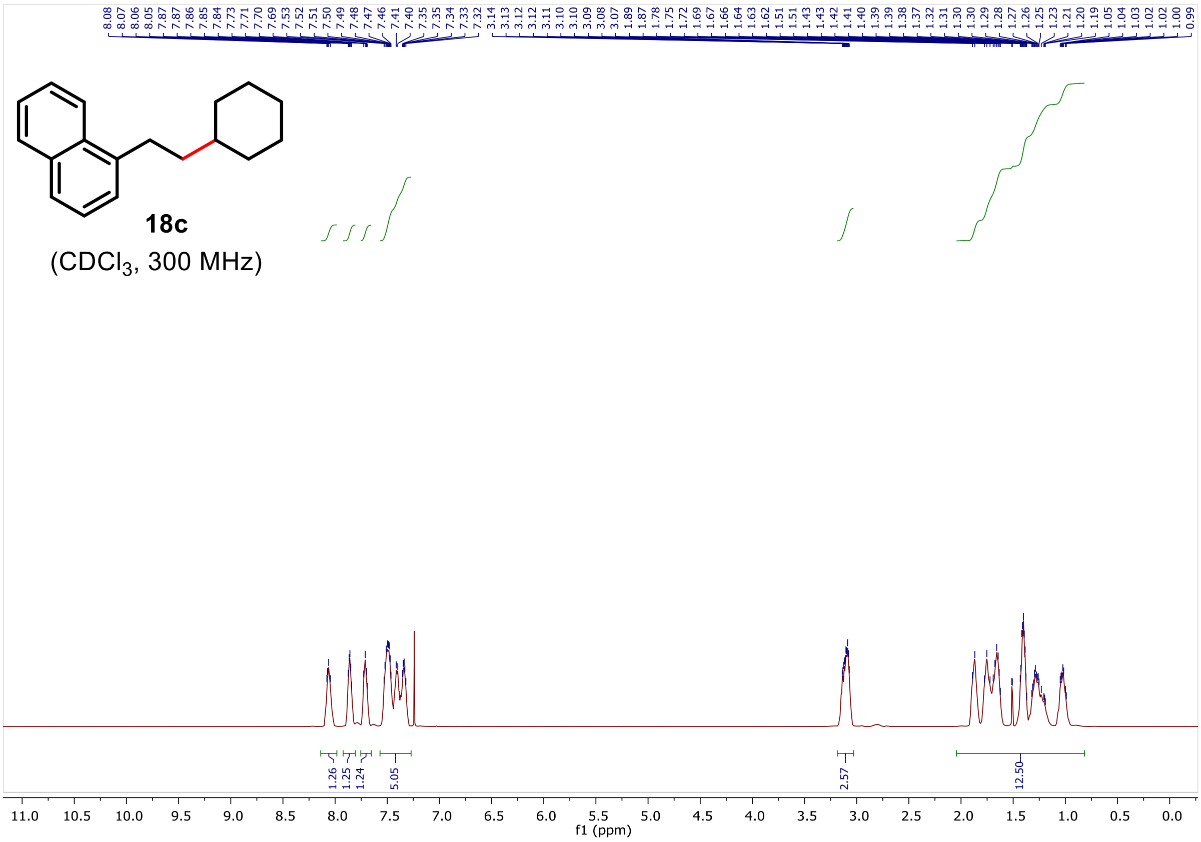
18c
(CDCl3, 300 MHz)
8.08 8.07 8.06 8.05 7.87 7.87 7.86 7.85 7.84 7.73 7.71 7.70 7.69 7.53 7.52 7.51 7.50 7.49 7.48 7.47 7.46 7.41 7.40 7.35 7.35 7.34 7.33 7.32 3.14 3.13 3.12 3.11 3.10 3.10 3.09 3.08 3.07 1.89 1.87 1.78 1.75 1.72 1.69 1.67 1.66 1.64 1.63 1.62 1.51 1.51 1.43 1.43 1.42 1.41 1.40 1.39 1.39 1.38 1.37 1.32 1.31 1.30 1.30 1.29 1.28 1.27 1.26 1.25 1.23 1.21 1.20 1.19 1.05 1.04 1.03 1.03 1.02 1.02 1.00 0.99
1.26 1.25 1.24 5.05 2.57 12.50
f1 (ppm)

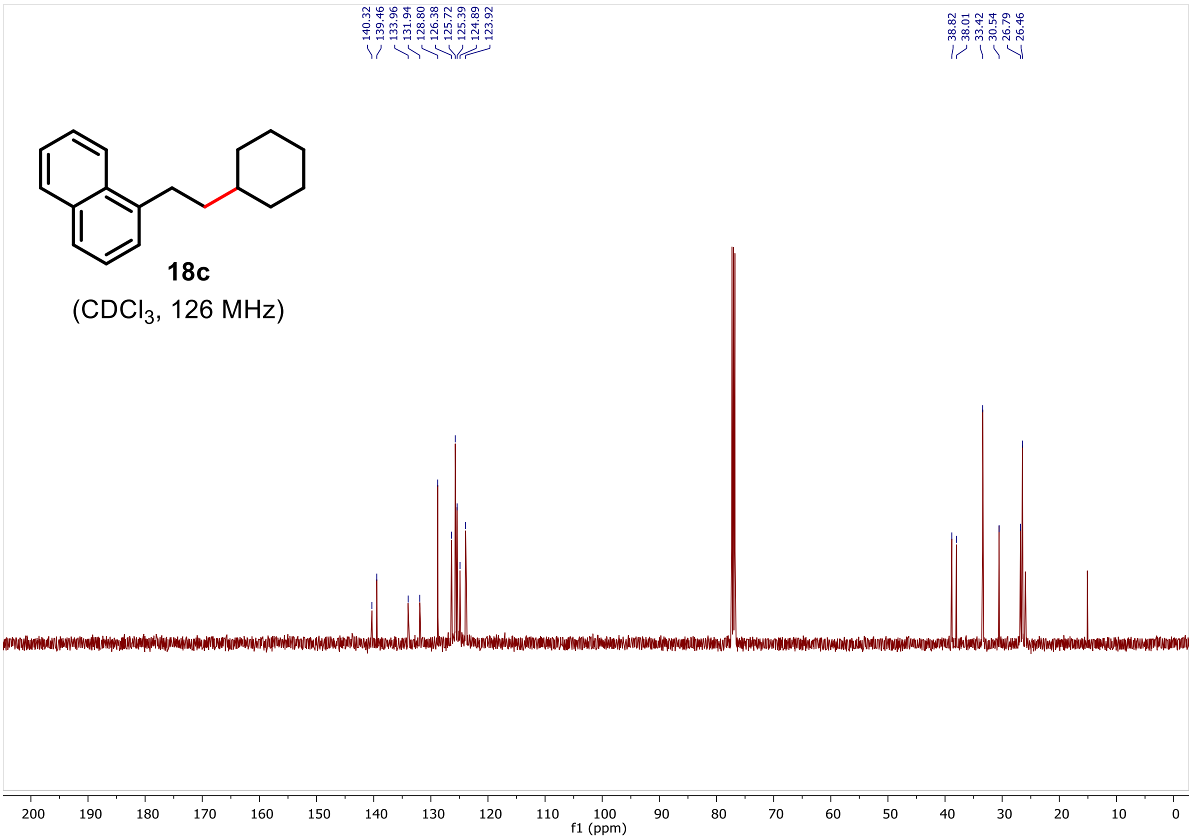
18c
(CDCl3, 126 MHz)
140.32 139.46 133.96 131.94 128.80 126.38 125.72 125.39 124.89 123.92
38.82 38.01 33.42 30.54 26.79 26.46
f1 (ppm)

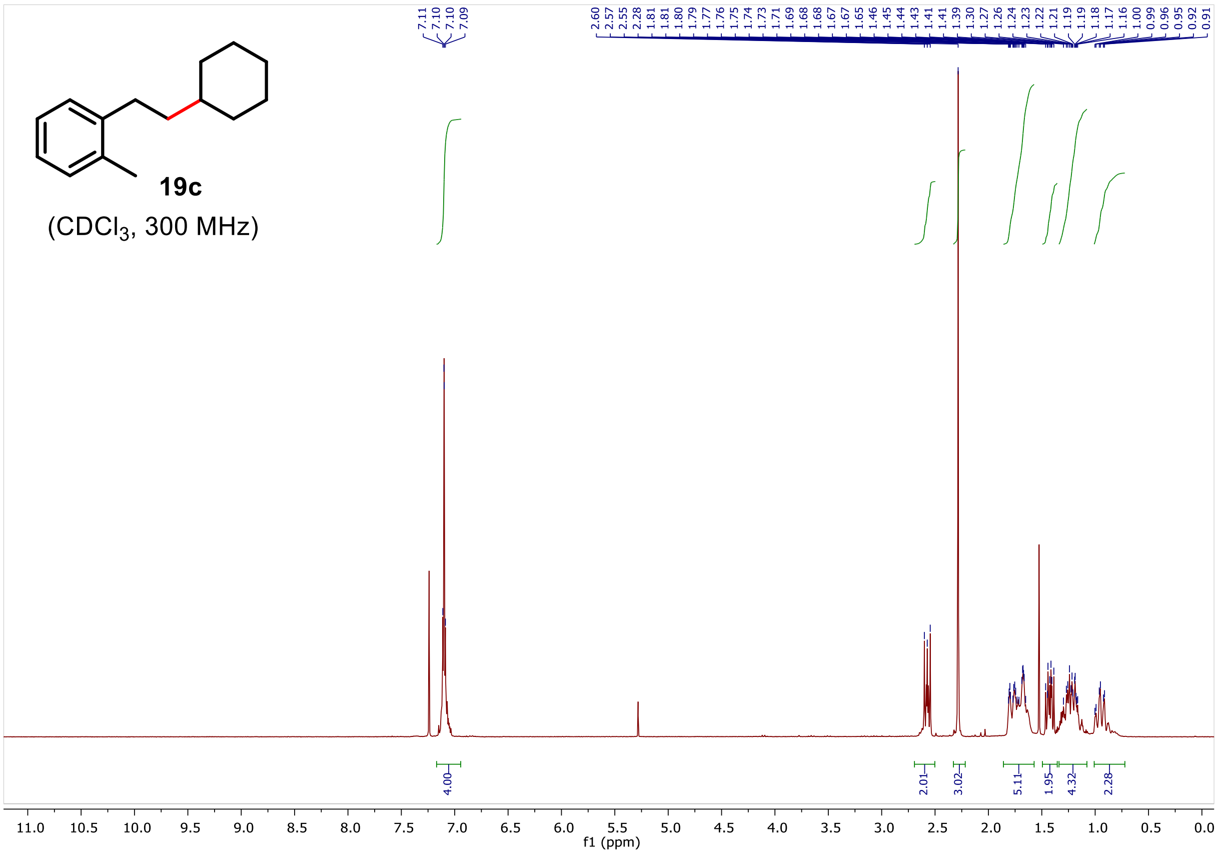
7.11
7.10
7.10
7.09
2.60
2.57
2.55
2.28
1.81
1.81
1.80
1.79
1.77
1.76
1.75
1.74
1.73
1.71
1.69
1.68
1.68
1.67
1.67
1.65
1.46
1.45
1.44
1.43
1.41
1.41
1.39
1.30
1.27
1.26
1.24
1.23
1.22
1.21
1.19
1.19
1.18
1.17
1.16
1.00
0.99
0.96
0.95
0.92
0.91
19c
(CDCl$_3$, 300 MHz)
4.00
2.01
3.02
5.11
1.95
4.32
2.28
11.0 10.5 10.0 9.5 9.0 8.5 8.0 7.5 7.0 6.5 6.0 5.5 5.0 4.5 4.0 3.5 3.0 2.5 2.0 1.5 1.0 0.5 0.0
f1 (ppm)

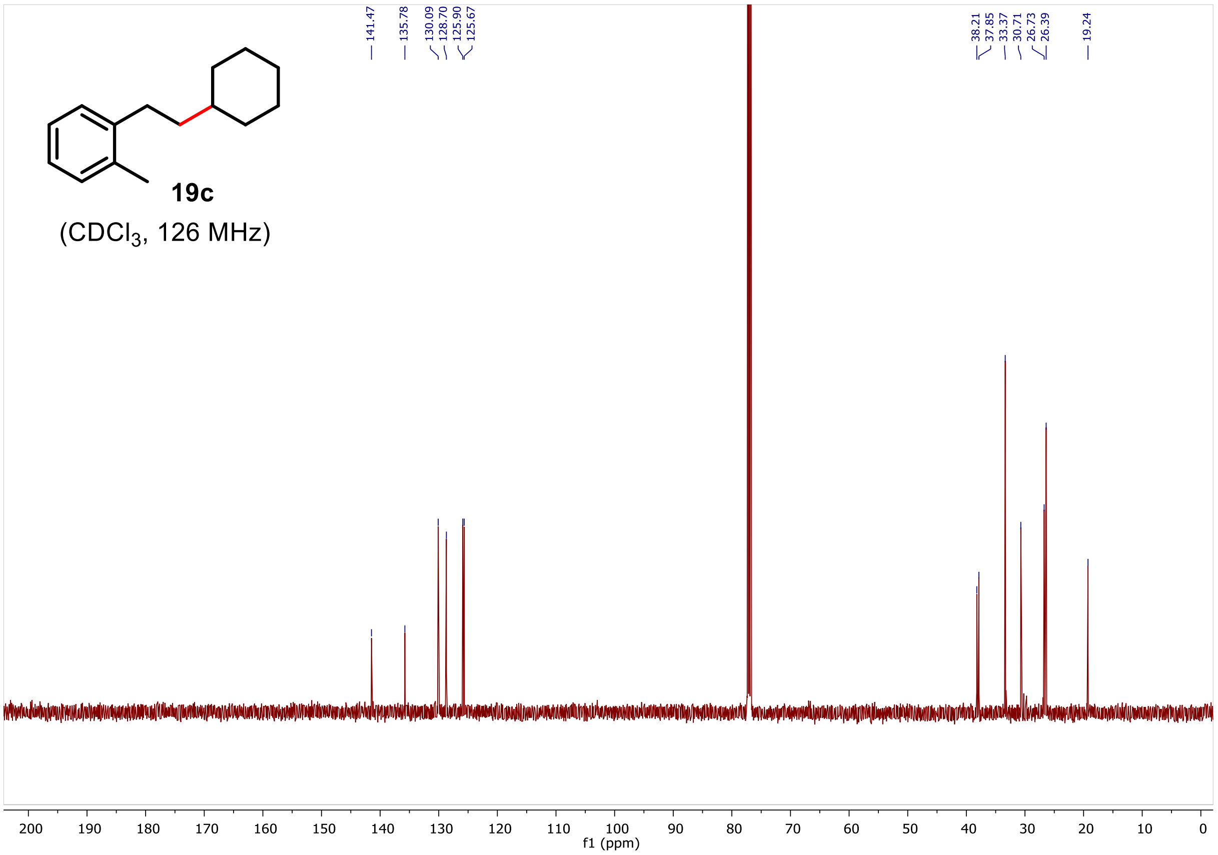
141.47
135.78
130.09
128.70
125.90
125.67
38.21
37.85
33.37
30.71
26.73
26.39
19.24
19c
(CDCl$_3$, 126 MHz)
200 190 180 170 160 150 140 130 120 110 100 90 80 70 60 50 40 30 20 10 0
f1 (ppm)

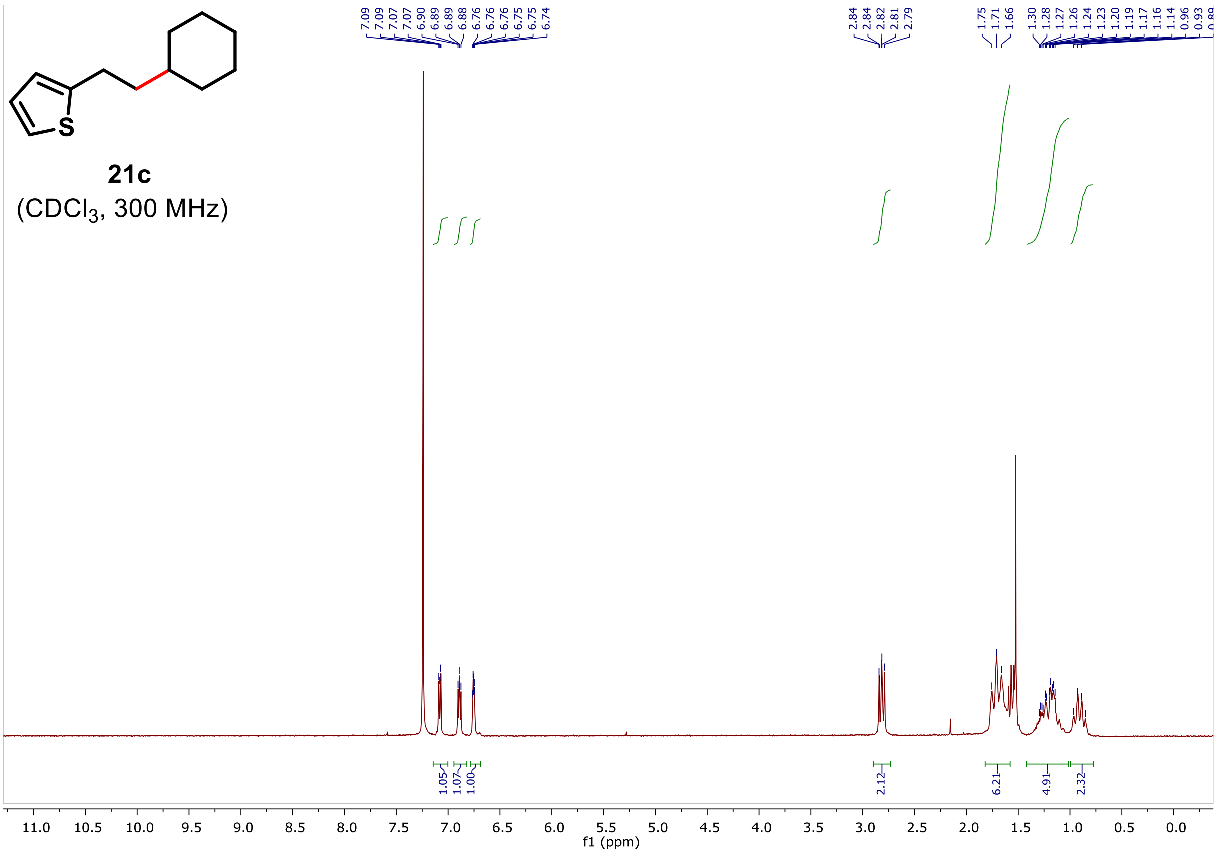
21c
(CDCl$_3$, 300 MHz)
7.09 7.09 7.07 7.07 6.90 6.89 6.89 6.88 6.76 6.76 6.75 6.75 6.75 6.74
2.84 2.84 2.82 2.81 2.79
1.75 1.71 1.66
1.30 1.28 1.27 1.26 1.24 1.23 1.20 1.19 1.17 1.16 1.14 0.96 0.93
1.05 1.07 1.00 2.12 6.21 4.91 2.32
f1 (ppm)

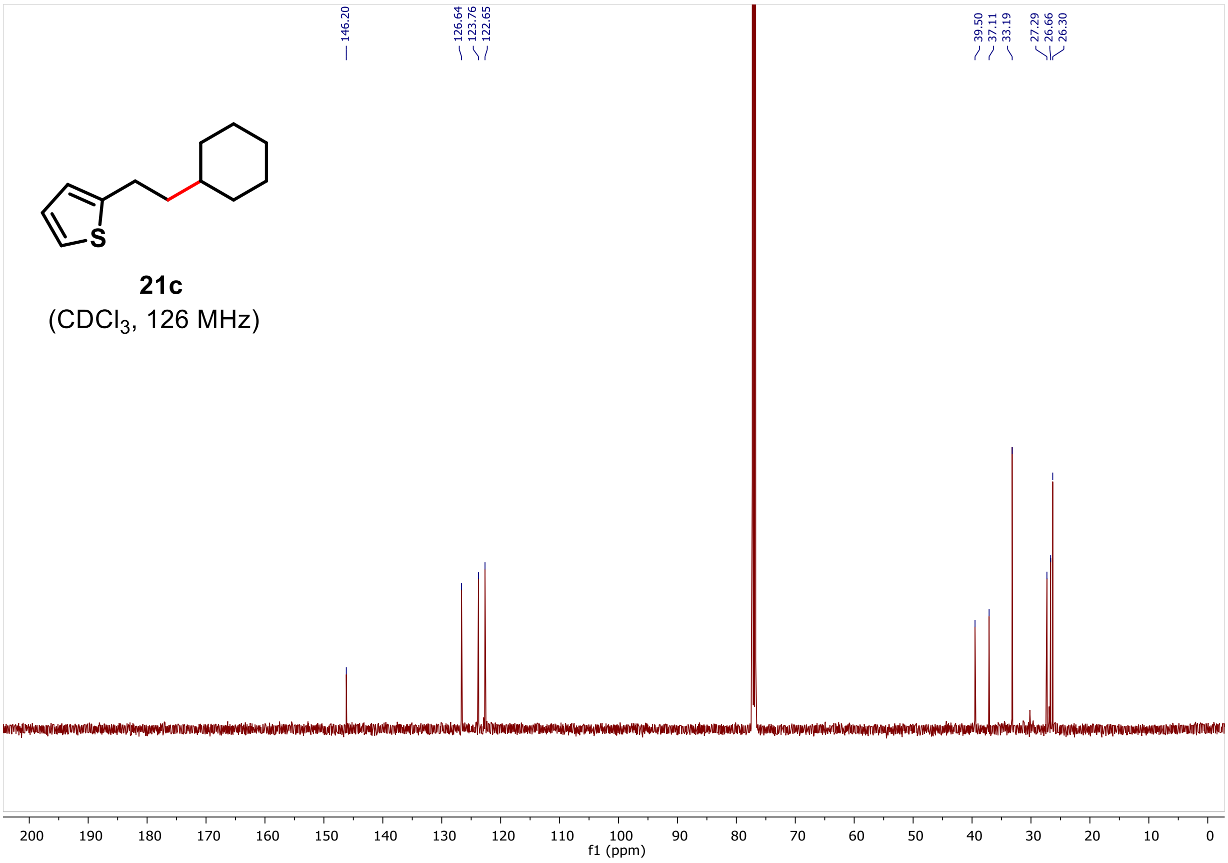
21c
(CDCl$_3$, 126 MHz)
146.20
126.64 123.76 122.65
39.50 37.11 33.19
27.29 26.66 26.30
f1 (ppm)

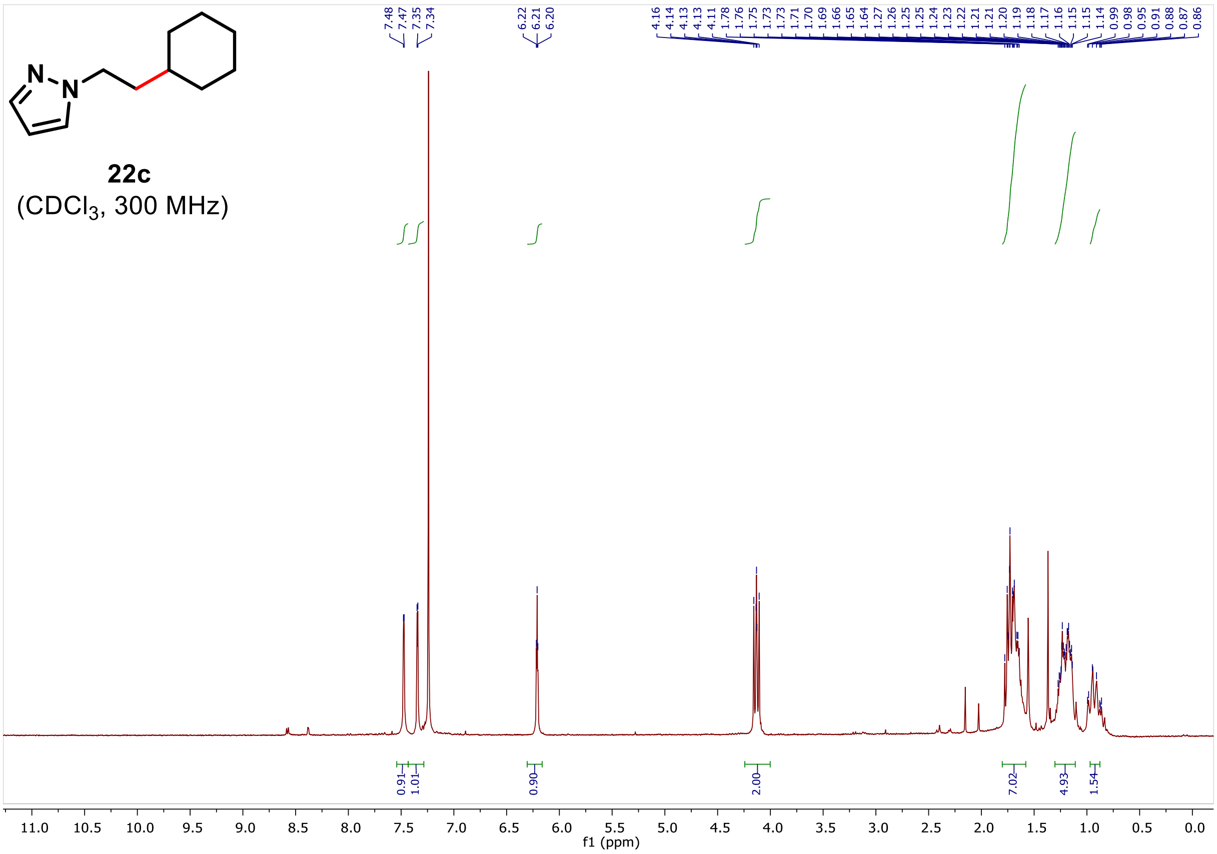

7.48
7.47
7.35
7.34
6.22
6.21
6.20
4.16
4.14
4.13
4.13
4.11
1.78
1.76
1.75
1.73
1.73
1.71
1.70
1.69
1.66
1.65
1.64
1.27
1.26
1.25
1.25
1.24
1.23
1.22
1.21
1.21
1.20
1.19
1.18
1.17
1.16
1.15
1.14
0.99
0.98
0.95
0.91
0.88
0.87
0.86
N
N
22c
(CDCl3, 300 MHz)
0.91
1.01
0.90
2.00
7.02
4.93
1.54
11.0 10.5 10.0 9.5 9.0 8.5 8.0 7.5 7.0 6.5 6.0 5.5 5.0 4.5 4.0 3.5 3.0 2.5 2.0 1.5 1.0 0.5 0.0
f1 (ppm)

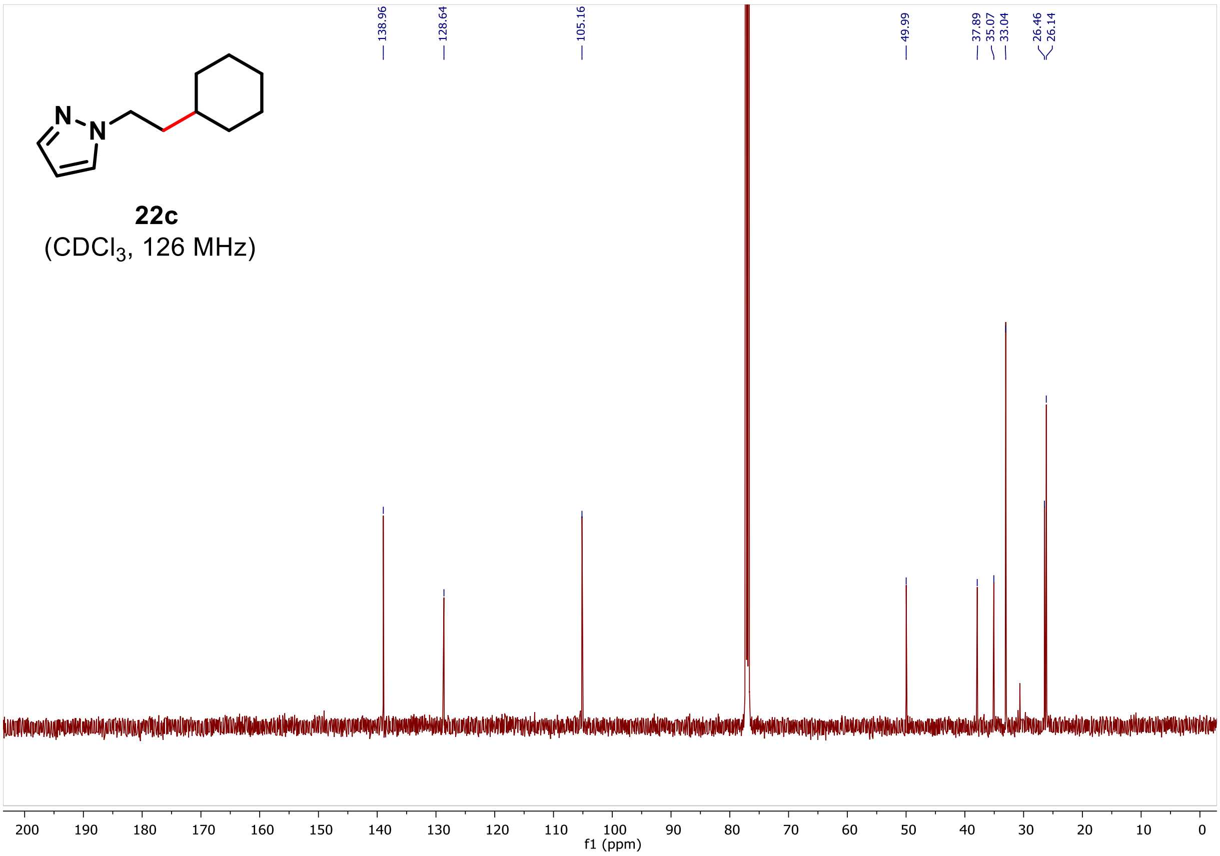

138.96
128.64
105.16
49.99
37.89
35.07
33.04
26.46
26.14
N
N
22c
(CDCl3, 126 MHz)
200 190 180 170 160 150 140 130 120 110 100 90 80 70 60 50 40 30 20 10 0
f1 (ppm)

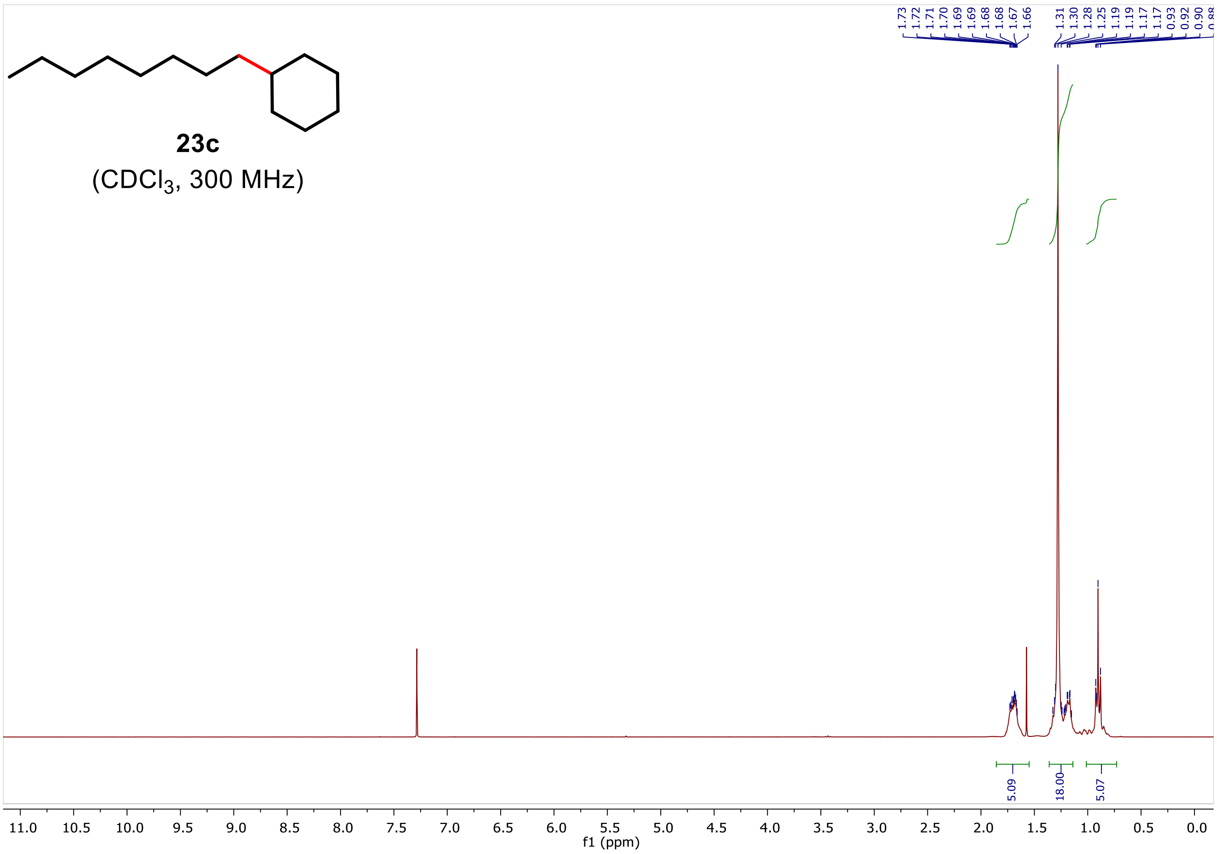
23c
(CDCl3, 300 MHz)
1.73
1.72
1.71
1.70
1.69
1.69
1.68
1.67
1.66
1.31
1.30
1.28
1.25
1.19
1.19
1.17
1.17
0.93
0.92
0.90
5.09
18.00
5.07
11.0 10.5 10.0 9.5 9.0 8.5 8.0 7.5 7.0 6.5 6.0 5.5 5.0 4.5 4.0 3.5 3.0 2.5 2.0 1.5 1.0 0.5 0.0
f1 (ppm)

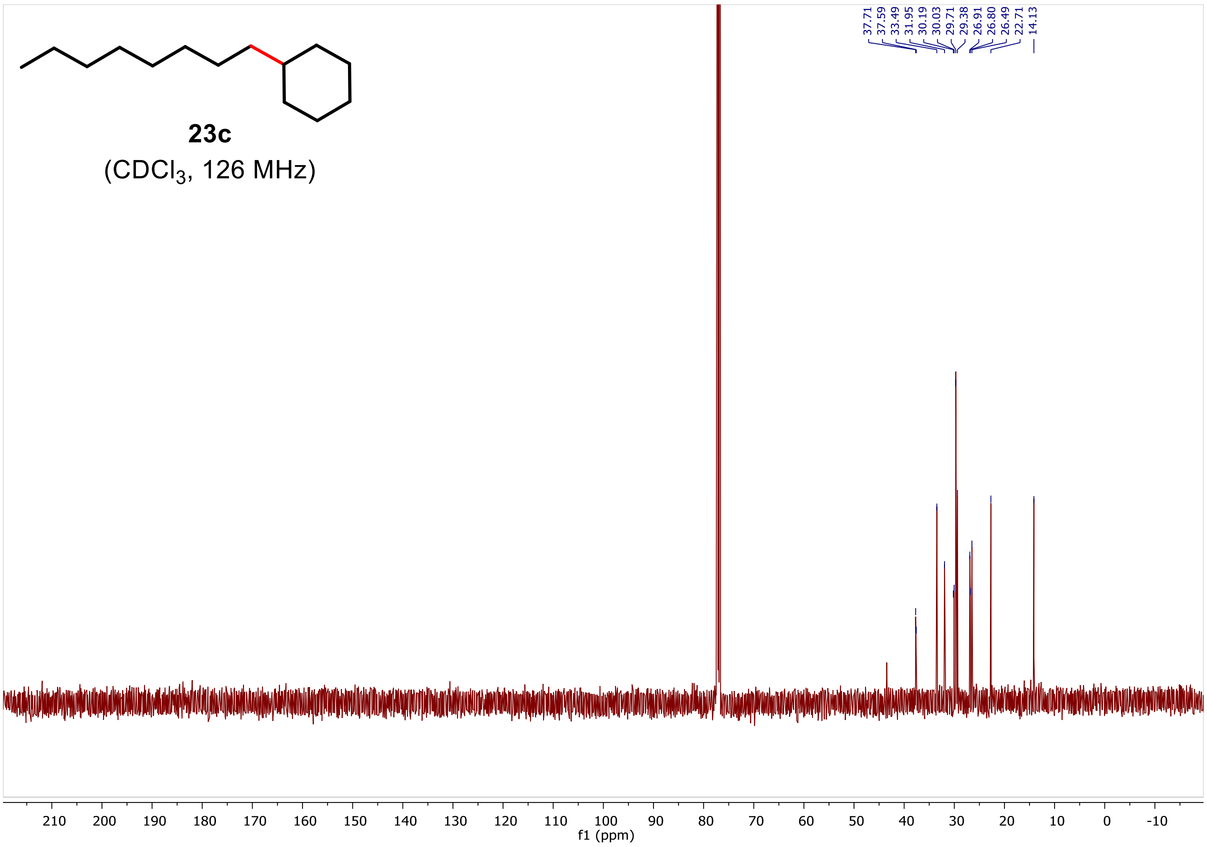
23c
(CDCl3, 126 MHz)
37.71
37.59
33.49
31.95
30.19
30.03
29.71
29.38
26.91
26.80
26.49
22.71
14.13
210 200 190 180 170 160 150 140 130 120 110 100 90 80 70 60 50 40 30 20 10 0 -10
f1 (ppm)

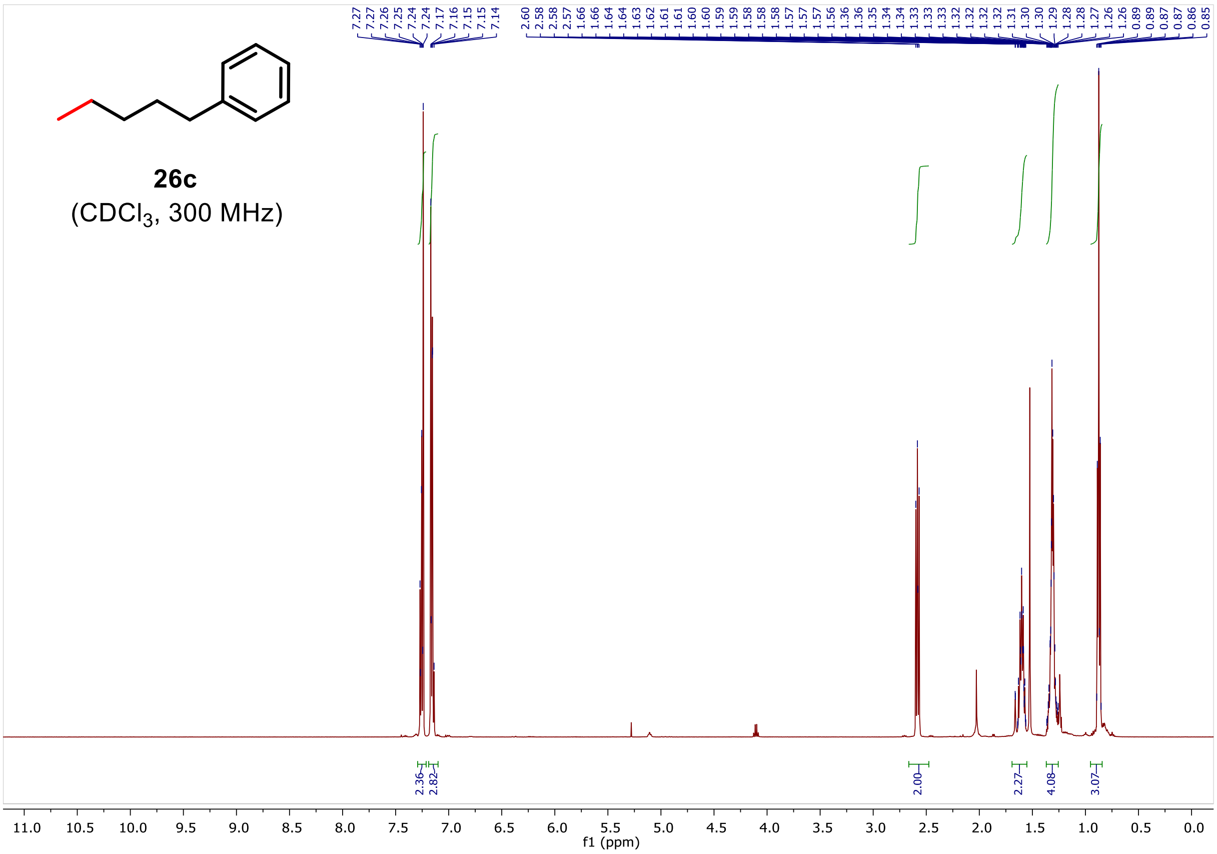
26c
(CDCl3, 300 MHz)
f1 (ppm)

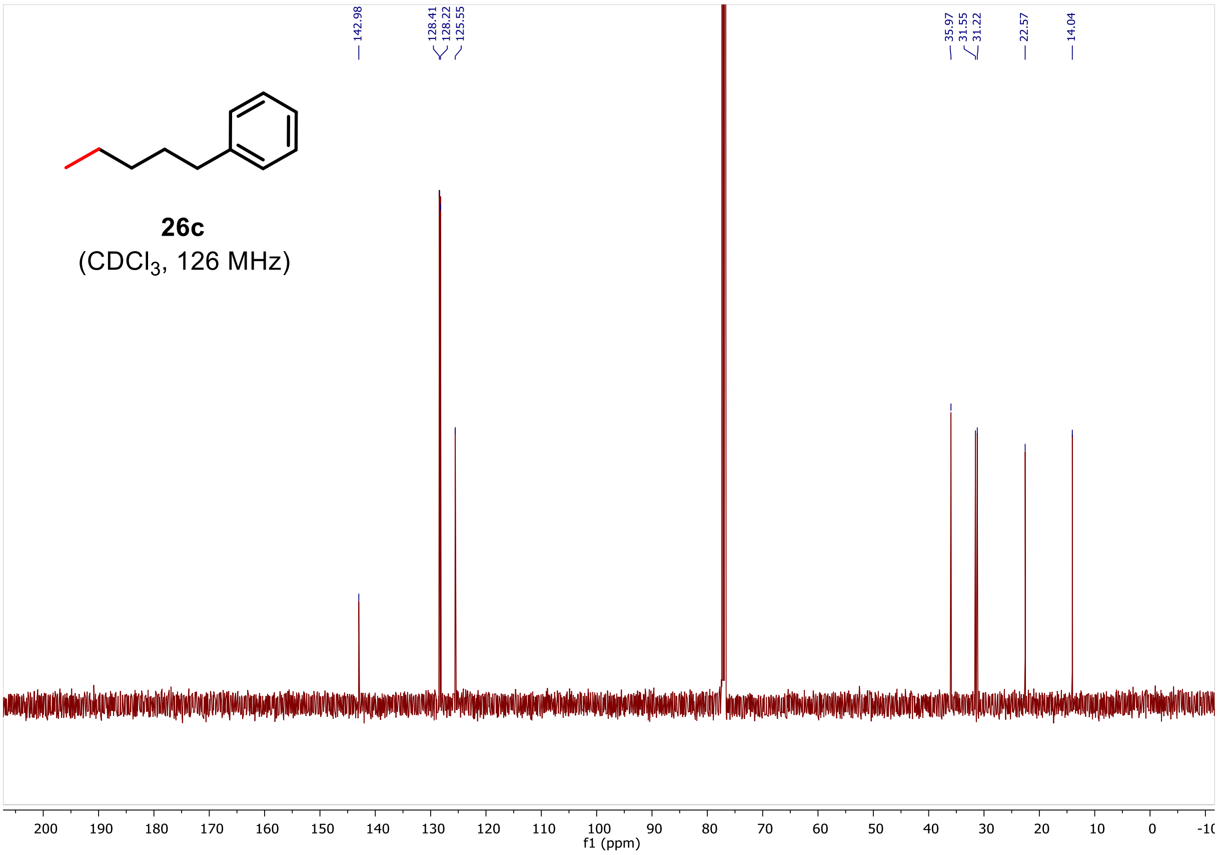
142.98
128.41
128.22
125.55
35.97
31.55
31.22
22.57
14.04
26c
(CDCl3, 126 MHz)
f1 (ppm)

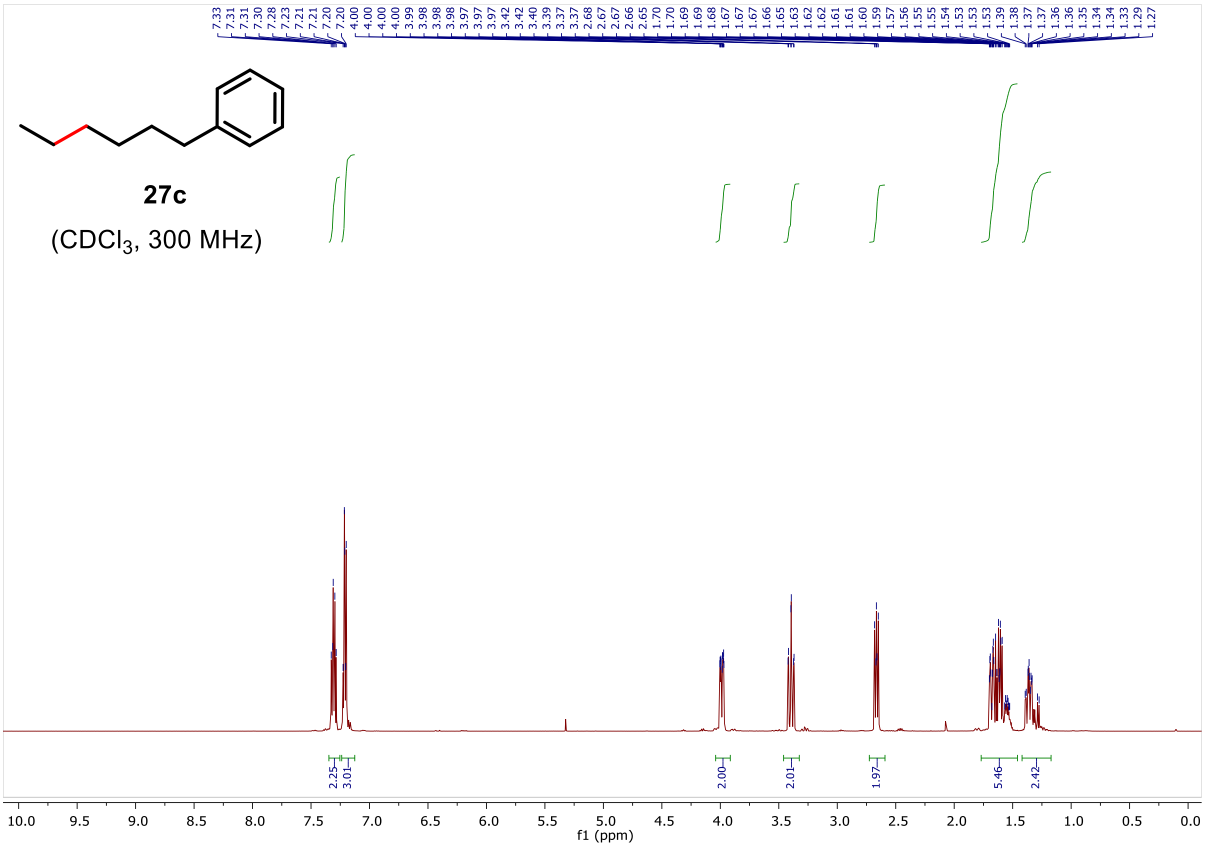
27c
(CDCl3, 300 MHz)
f1 (ppm)

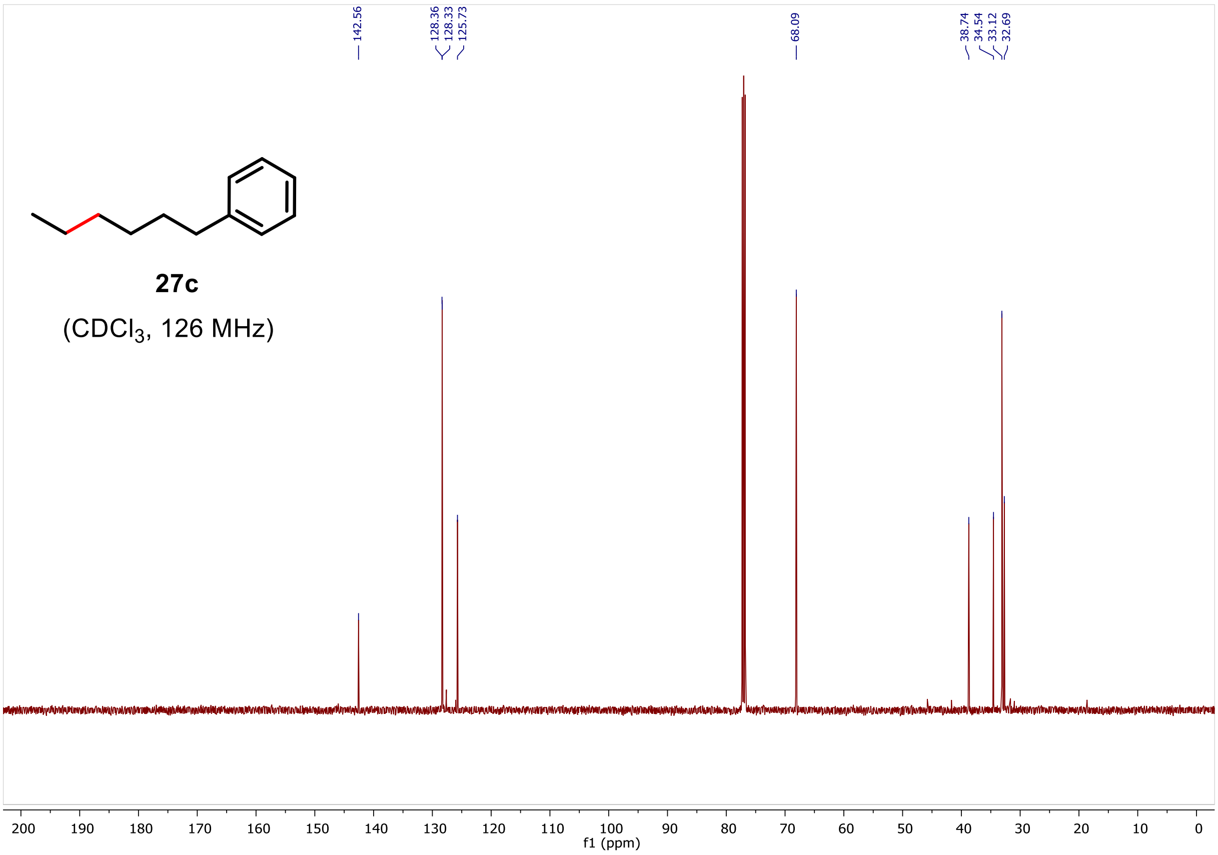
142.56
128.36
128.33
125.73
68.09
38.74
34.54
33.12
32.69
27c
(CDCl3, 126 MHz)
f1 (ppm)

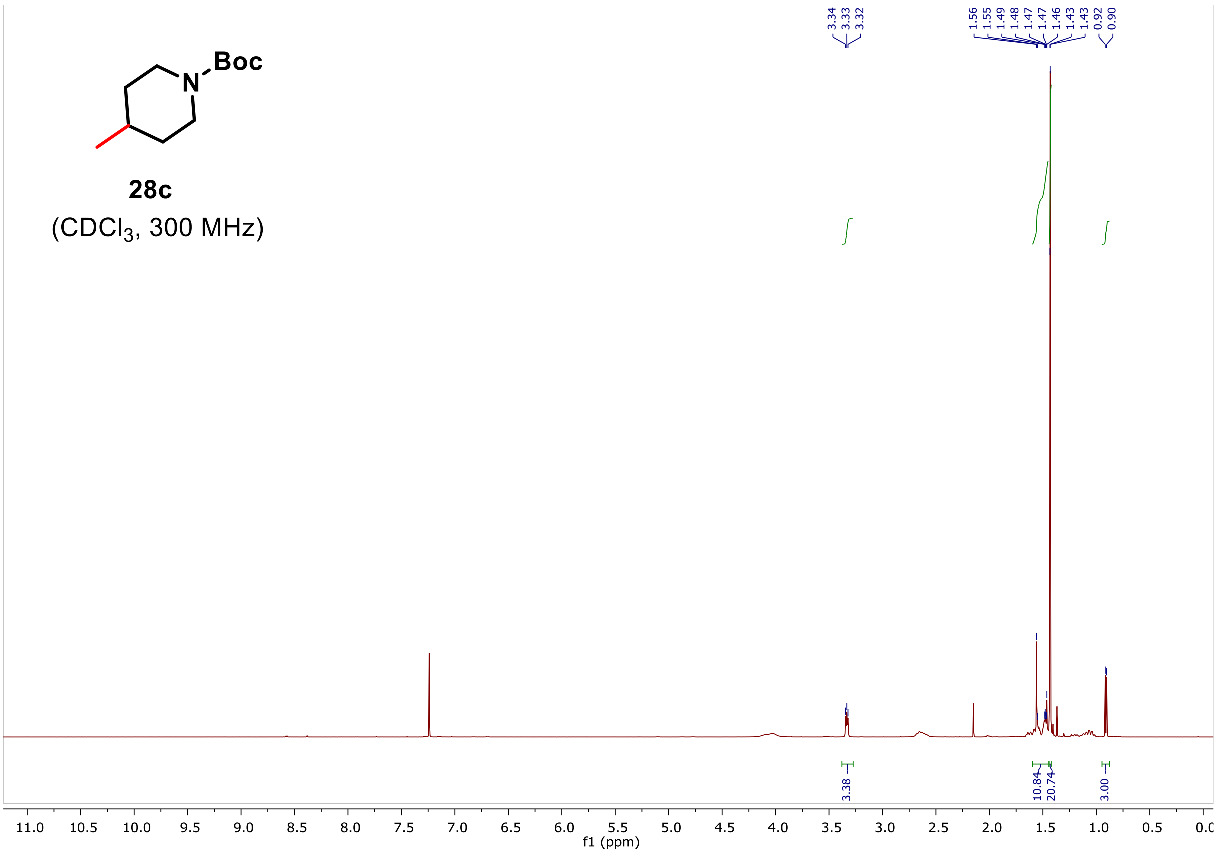
Boc
N
28c
(CDCl3, 300 MHz)
3.34
3.33
3.32
1.56
1.55
1.49
1.48
1.47
1.47
1.46
1.43
1.43
0.92
0.90
3.38
10.84
20.74
3.00
f1 (ppm)

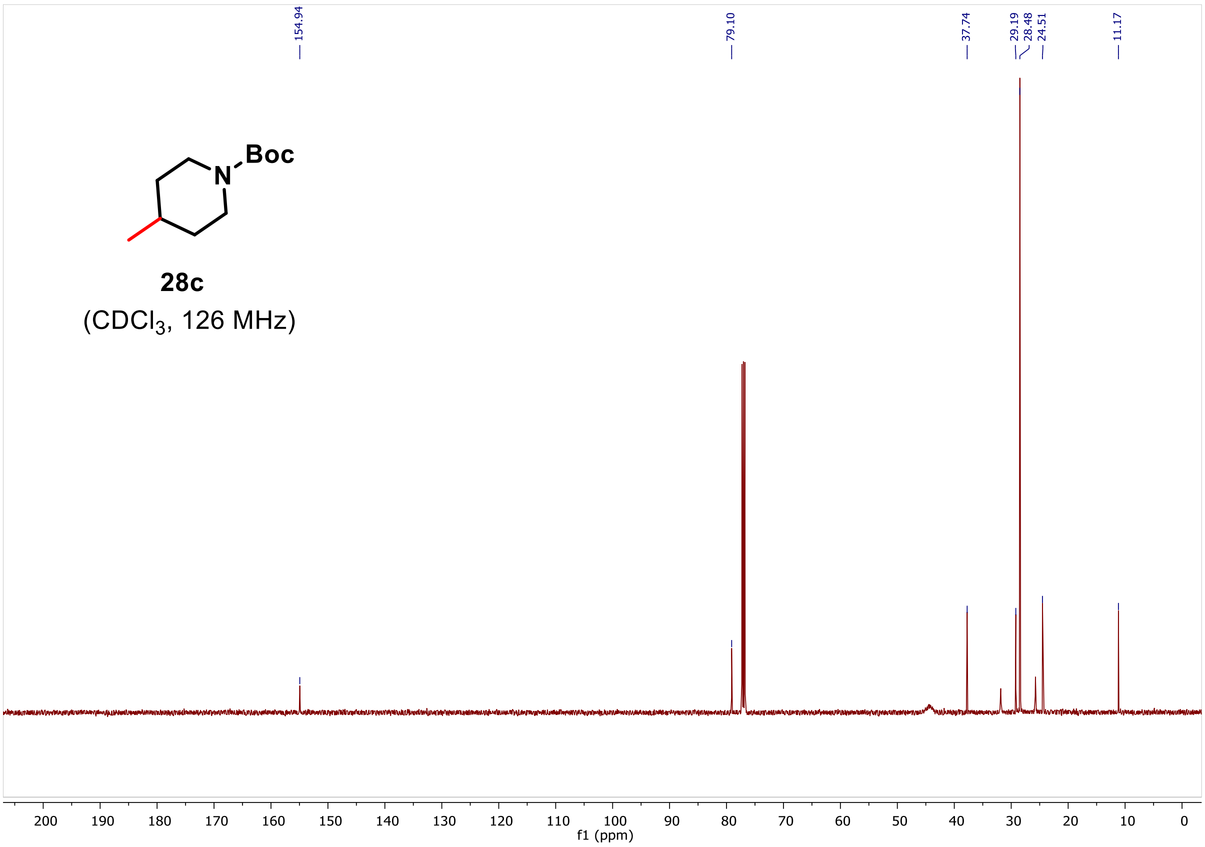
Boc
N
28c
(CDCl3, 126 MHz)
154.94
79.10
37.74
29.19
28.48
24.51
11.17
f1 (ppm)

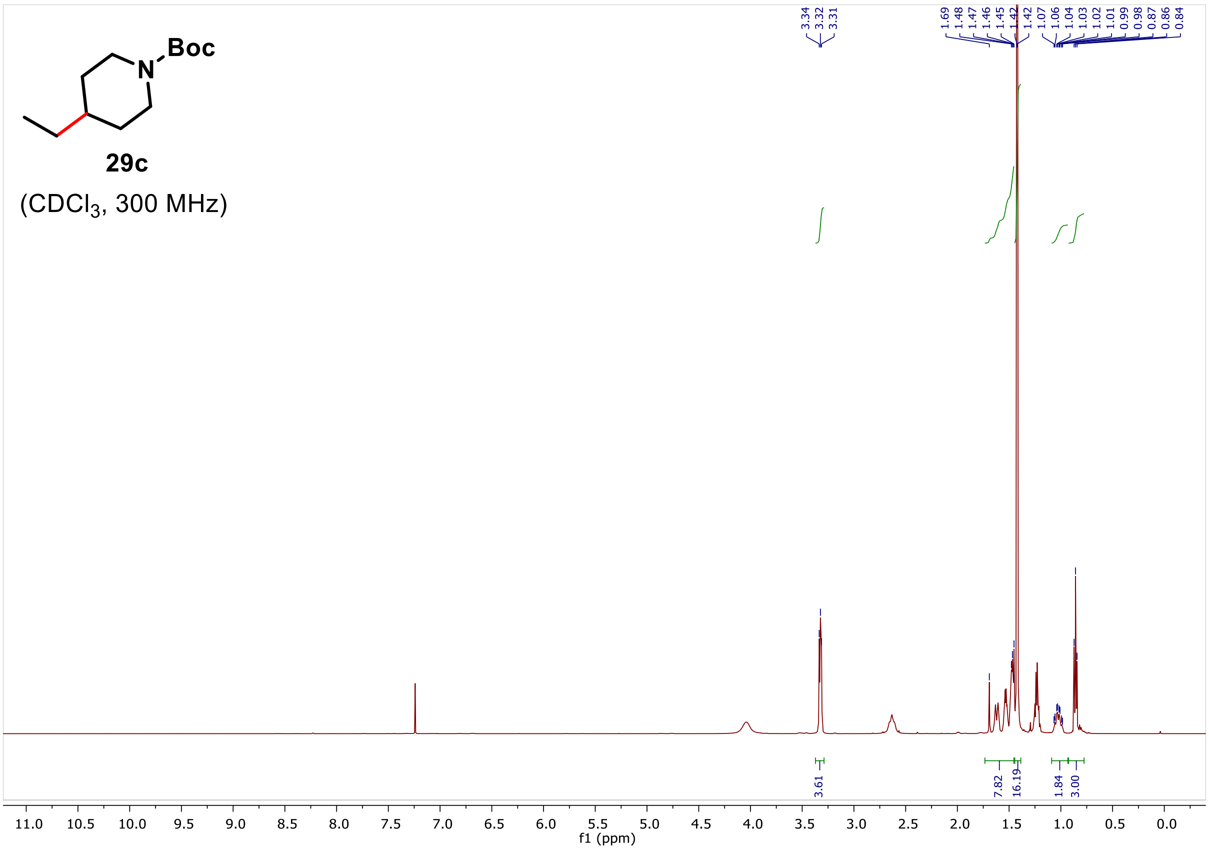
Boc
N
29c
(CDCl$_3$, 300 MHz)
3.34
3.32
3.31
1.69
1.48
1.47
1.46
1.45
1.42
1.42
1.07
1.06
1.04
1.03
1.02
1.01
0.99
0.98
0.87
0.86
0.84
3.61
7.82
16.19
1.84
3.00
11.0 10.5 10.0 9.5 9.0 8.5 8.0 7.5 7.0 6.5 6.0 5.5 5.0 4.5 4.0 3.5 3.0 2.5 2.0 1.5 1.0 0.5 0.0
f1 (ppm)

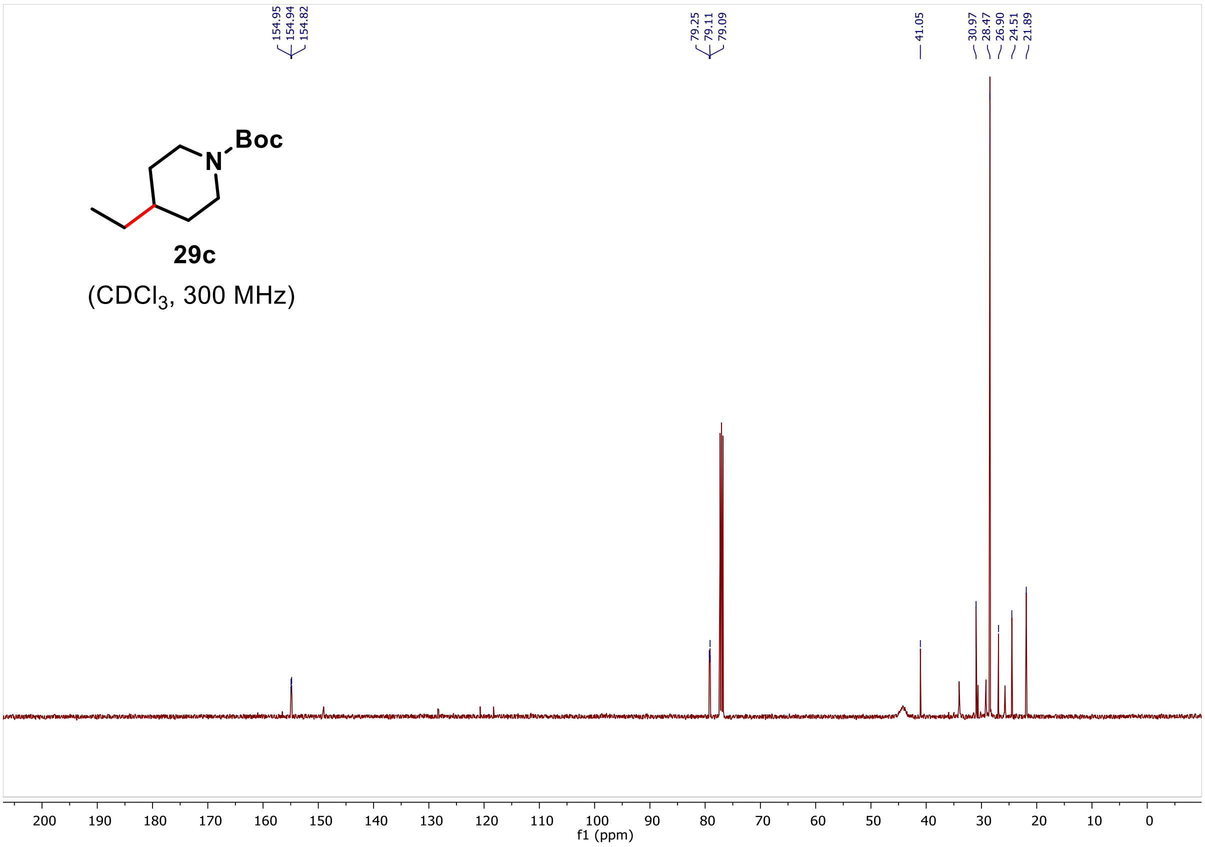
154.95
154.94
154.82
79.25
79.11
79.09
41.05
30.97
28.47
26.90
24.51
21.89
Boc
N
29c
(CDCl$_3$, 300 MHz)
200 190 180 170 160 150 140 130 120 110 100 90 80 70 60 50 40 30 20 10 0
f1 (ppm)

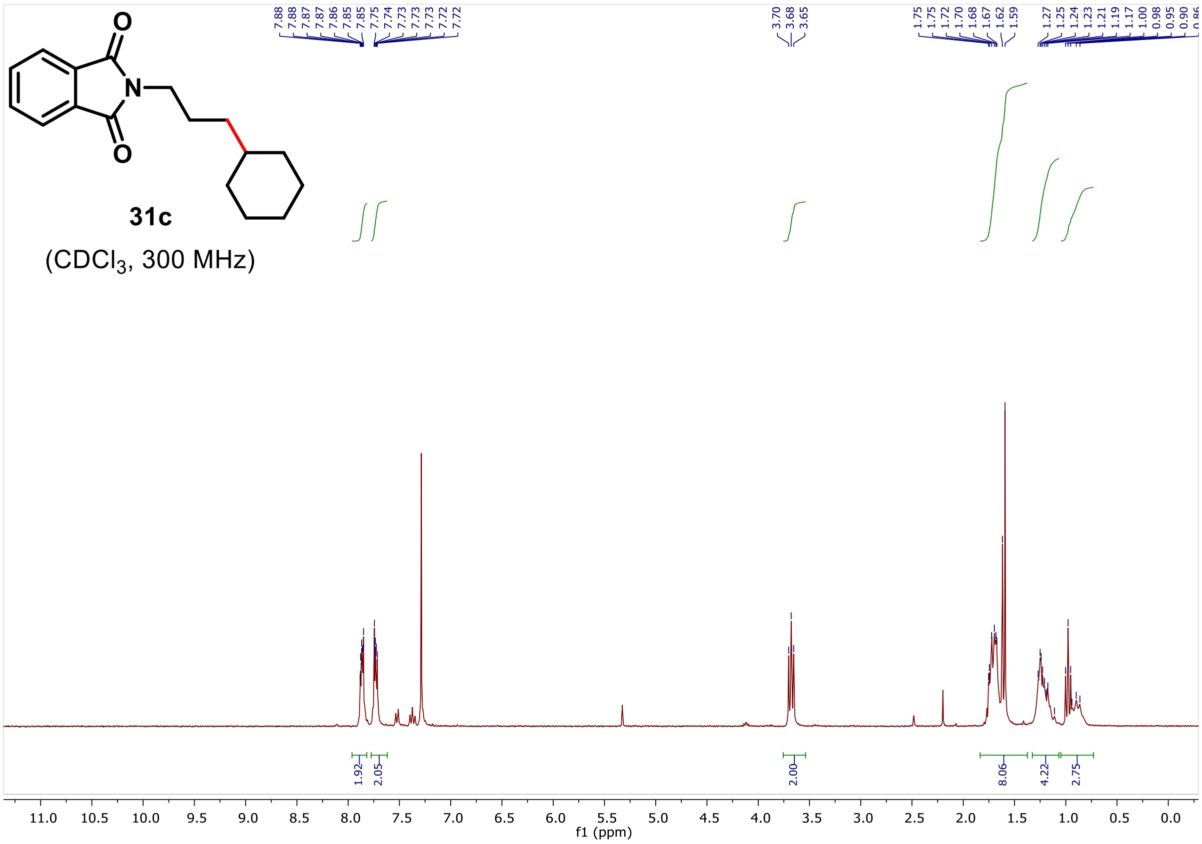
O
N
O
31c
(CDCl$_3$, 300 MHz)
7.88
7.88
7.87
7.87
7.86
7.85
7.85
7.75
7.74
7.73
7.73
7.73
7.72
7.72
3.70
3.68
3.65
1.75
1.75
1.72
1.70
1.68
1.67
1.62
1.59
1.27
1.25
1.24
1.23
1.21
1.19
1.17
1.00
0.98
0.95
0.90
1.92
2.05
2.00
8.06
4.22
2.75
11.0 10.5 10.0 9.5 9.0 8.5 8.0 7.5 7.0 6.5 6.0 5.5 5.0 4.5 4.0 3.5 3.0 2.5 2.0 1.5 1.0 0.5 0.0
f1 (ppm)

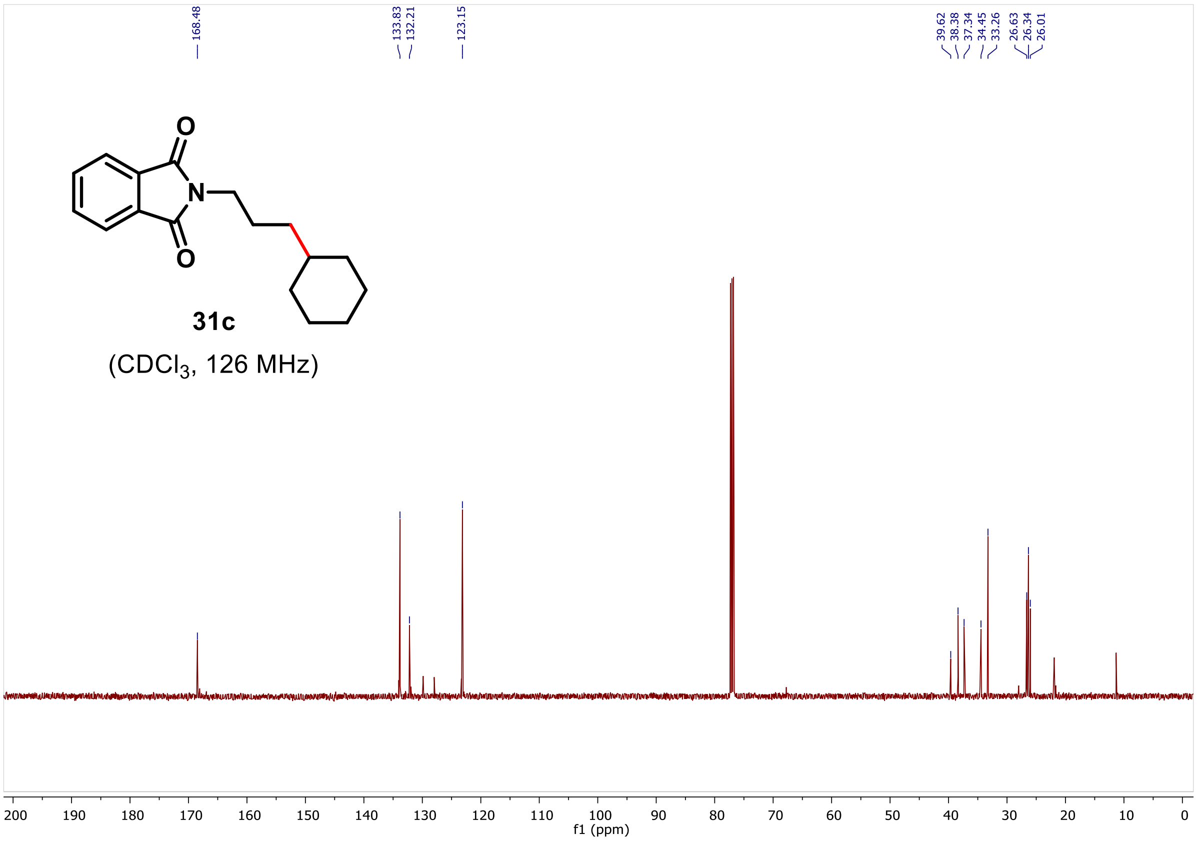
168.48
133.83
132.21
123.15
39.62
38.38
37.34
34.45
33.26
26.63
26.34
26.01
O
N
O
31c
(CDCl$_3$, 126 MHz)
200 190 180 170 160 150 140 130 120 110 100 90 80 70 60 50 40 30 20 10 0
f1 (ppm)
